# Predicting opioid consumption after surgical discharge: a multinational derivation and validation study using a foundation model

**DOI:** 10.1038/s41746-025-01798-6

**Published:** 2025-08-26

**Authors:** Chris Varghese, Luke Peters, Lorane Gaborit, William Xu, Kaviya Kalyanasundaram, Aya Basam, Melissa Park, Cameron Wells, Kenneth A. McLean, Gabriel Schamberg, Greg O’Grady, Deborah Wright, Jennifer Martin, Ewen Harrison, Peter Pockney, TASMAN Collaborative, Chris Varghese, Luke Peters, Lorane Gaborit, Kaviya Kalyanasundaram, Aya Basam, Deborah Wright, Jennifer Martin, Melissa Park, Peter Pockney, William Xu, Cameron Wells, Gabriel Schamberg, Greg O’Grady, Kenneth A. McLean, Ewen Harrison, Billy Wu, Sarah Goh, Jiting Li, Jainil Shah, Abdullah Waraich, Upasana Pathak, Amie Hilder, Muhammed Elhadi, Aiden Jabur, Christina Ohis, Chui Foong Ong, Venesa Siribaddana, Kyle Raubenheimer, Jennifer Vu, Gordon Liu, Liam Ferguson, Kristy Atherton, Amanda Dawson, Arnab Banerjee, Nagendra Dudi-Venkata, Nicholas Lightfoot, Isabella Ludbrook, Luke Peters, Rachel Sara, David Watson, Ademola Adeyeye, Luis Adrian Alvarez-Lozada, Semra Demirli Atici, Milos Buhavac, Giacomo Calini, Orestis Ioannidis, Mustafa Deniz Tepe, Upanmanyu Nath, Ahmad Uzair, Wah Yang, Faseeh Zaidi, Surya Singh, Bahiyah Abdullah, Diana Sofia Garces Palacios, Ahmed Ragab, Ahmed Ahmed, Kyle Raubenheimer, Davina Daudu, Simran Vinod Benyani, Nandini Karthikeyan, Laure Taher Mansour, Warren Seow, Zoya Tasi, Dhia Errahmane Abdelmelek, Ikram Fatima Zohra Boussahel, Oumelaz Kaabache, Naoual Lemdaoui, Oualid Nebbar, Mounira Rais, Meriem Abdoun, Aya Tinhinane Kouicem, Souad Bouaoud, Kamel Bouchenak, Hind Saada, Amel Ouyahia, Wassila Messai, Zhi Shyuan Choong, Clarissa Ting, Michelle Larkin, Pei Jun Fong, Isabel Soh, Alyssia De Grandi, Hareem Iftikhar, Akansha Sinha, Dhruv Kapoor, Tara Chlebicka, David Singer, Kim Goddard, Lisa Matthews, Rosalina Lin, Jessica Chambers, Juliet Chan, Brooke Macnab, John Barker, Morgan Mckenzie, Neil Ferguson, Ghanisht Juwaheer, Vijayaragavan Muralidharan, Sonia Gill, Nakjun Sung, Rohan Patel, Chris Walters, Kevin Nguyen, David Liu, Carlos Cabalag, Jennifer Lee, San-Hui Anita Leow, Suat Li Ng, Hamza Ashraf, Fraizer Mulder, Jonathan Loo, David Proud, Samantha Wong, Yida Zhou, Qi Rui Soh, David Chye, Sean Stevens, Patrick Tang, Stephen Kritharides, Jason Dong, Oscar Morice, Dora Huang, Andrew Hardidge, Mishka Amarasekara, Aleah Kink, Damien Bolton, Alisha Rawal, Jasraaj Singh, Matthew Heard, Yusuf Hassan, Ahmed Naqeeb, Andrew Cobden, Duron Prinsloo, Dwain Quadros, Emma Gunn, Ha Jin Kim, Jennifer Ekwebelam, James Shanahan, Mustafa Alkazali, Mariyah Hoosenally, Naveen Nara, Peter Nguyen, Sally Barker, Zacchary Tamsett, Naomi Rigby, Hinal Patel, Eloise Ferguson, Lauren Byrnes, Alexander Bravo, Amie Hilder, Ally Hui, Antara Karmakar, Bill Wang, Janindu Goonawardena, King Tung Cheung, Nicholas Chan, Ragul Natarajan, Richard Cade, Rong Jin, Shomik Sengupta, Ruth Snider, Harsha Morisetty, Lewis Weeda, Phoebe Sun, Lalitya Chilaka, Jacinta Cover, Aashrinee De Silva Abeweera Gunasekara, Rahavi Senthilrajan, Anas Alwahaib, Alexandra Limmer, Bushra Zamanbandhon, Kumail Jaffry, Yijia Shen, Alan Chua, Saifulla Syed, Sushanth Saha, John Glynatsis, Lori Aitchison, Bernard Lagana, Mason Crossman, David Watson, Abby Dawson, Bryan Fong, Ella Harrison, Eleanor Horsburgh, Michael Khoo, Kritika Mishra, Lewis Hewton, Alex Mesecke, Hien Tu, Than Tun, Jason Wong, Elynn Ong, Tara-Nyssa Law, Ashlee Landy, Alyssa Leano, Andrea Li, Akshay Soni, Benjamin Dowdle, Charles Pilgrim, Dewmi Abeysirigunawardana, Deepak Rajan Jeyarajan, Diya Patel, Jason Chung, Kyle Mckinnon, Madeline Gould, Paul Gilmore, Ruxi Geng, Rachael Loughnan, Sarahjane Norton-Smith, Solomon Nyame, Sarah Tan, Sewni Samarawickrama, Si Woo Yoon, Yantong Wang, Yichi Zhang, Zixuan Wang, Hans Mare, Indrajith Withanage, Mitali Khattar, Alexandra Toft, Goutham Sivasuthan, Hailin Zhao, Jordan Addley, Lucinda O’brien, Muhammad Raza, Randipsingh Bindra, Sonakshi Sharma, Charlotte Cornwell, Aditya Patil, Aiden Cheung, Ashleigh Lown, Amanda Dawson, Aneel Blassey, Benjamin Ochigbo, Felicity Cheng, Aleeza Fatima, Edward Zhang, Henry Kocatekin, Charles Roth, Dani Brewster, Kelvin Kwok, Paul Chen, Sharon Laura, Dominic Tynan, Edward Latif, Elizabeth Lun, Elodie Honore, Felix Ziergiebel, Jessica Blake, Karan Chandiok, Katie Bird, Lynette Ngothanh, Melissa Lee, Mariam El-Masry, Peter Hamer, Ramanathan Rm Palaniappan, Richard Mcgee, Sarah Huang, Shane Zhang, Shubhang Hariharan, Yannick De Silva, Celeste Lee, Penelope Fotheringham, Ian Incoll, Timothy Cordingley, Matthew Brown, Leannedra Kang, Rivindu Wijayaratne, Parisse Moore, Gemma Qian, Yara Elgindy, Emma Carnuccio, Hamish Rae, Mena Shehata, Mingchun Liu, Brodee Lockwood, John Van Bockxmeer, Ali Alsoudani, Daniel Swan, Justin Hsieh, Francesca Orchard-Hall, Kai Yun Jodene Tay, Raagini Mehra, Alpha Gebeh, Ashley Bailey, Georgia Brown, Ashley Colaco, Hemashree Gopal, Jessica Boyley, Varun Changati, Joseph Fletcher, Tanishq Khandelwal, Colin House, Chris O’neil, Emily Jaarsma, Victor Ly, Zsolt Balogh, Amanda Shui, Vinogi Sathasivam, Hannah Legge-Wilkinson, King Ho Wong, Andrew Chen, Anthony Tran, Peter Rehfisch, Grace Wang, Jonathan Nguyen, Joshua Peker, Kayla Gallert, Mia Komesaroff, Manideep Namburi, Elisabeth Goldfinch, Ropafadzo Muchabaiwa, Aishwarya Jangam, Isobel Taylor, Iulian Nusem, Jin Hyuk (David) Park, Justin Gundara, Rachael Heigan, Tam Tran, Thomas Mackay, Yasmine Butterworth, Tomas Sadauskas, Melody Tung, Hasthika Ellepola, Christine Gan, Hakim Fong, Ankita Das, Leshya Naicker, Samantha Hauptman, Aditi Kamath, Anthea Yew, Anupam Parange, Katie Kim, Sahil Kharwadkar, Tharushi Gamage, Lucille Vance, Alexandra Seldon, Moheb Ghaly, Jainam Shah, Victoria Phan, Karanjeet Chauhan, Ahmad Bassam, Beverley Vollenhoven, Kumail Jaffry, Kajal Mandhan, Mithra Sritharan, Mahesh Sakthivel, Natalie Evans, Samuel Robinson, Seiyon Sivakumar, Liberty Marrison, David Jollow, Krishma Joshi, Steve Tao, Pallavi Shrestha, Sai Keerthana Nukala, Russell Hodgson, Anna Crotty, Adriana Esho, Alasdair Harris, Amy Surkitt, Laura Bland, Blake Mcleod, Chonghao Yin, Cambo Keng, Emily Greenwood, Grace Yuan, Emma Haege, Hongyi Wu, Haotian Xiao, Isabella Pozzi, Jeff Fu, Jessica Stott Ross, Juliette Gentle, Kathy Gan, Kelvin Chang, Kexin Sun, Madhavi Singh, Maria Xie, Nicholas Mccabe, Mark Slavec, Nick Clarnette, Behzad Niknami, Peishan Zou, Sean Flintoft, Shenuka Jayatilleke, Rumnea Sok, Suqi Tan, Sanya Wadhwa, Will Swansson, Daniel Abulafia, Jian Blundell, Amie Sweetapple, Caitlin Del Solar, Cameron Martin, David Bell, Isuru Fernando, Jared Chang, Katie Vanzuylekom, Katie Van Zuylekom, Kate Van Zuylekom, Katie Hobbs, Richard Liang, Aiden Jabur, Jazmina Tarmidi, Mahmoud Ugool, Nicholas Beatson, Sarah Bowman, Sophie Moin, Wen Po Jonathan Tan, Seevakan Chidambaram, Siang Wei Gan, Pengnan Wang, Leshya Naicker, Katie Kim, Nicole Qiwen Wang, Yi Xin Kwan, Chinmai Patil, Divyanshu Joshi, Aditi Kamath, Aishath Hanan, Arfaan Sheriff, Jaime Duffield, Leshya Naiker, Peter Smitham, Eu Ling Neo, Matthew Chua, Shalvin Prasad, Armitesh Nagaratnam, Tarik Sammour, Yuxin Lin, Christine Lee, Eve Hopping, Muskan Jangra, Ankita Das, Ken Lin, Zachary Bunjo, Kyle Raubenheimer, Mohamed Haseef Mohamed Yunos, Kar Long Yeung, Rachel Phu, Aisling Betts, Benjamin Just, Sahil Gera, Hilary Leeson, Jodie Jamieson, Katie Wang, Emily Luu, Michael Innes, Jennifer Vu, Jonathan Hong, Stephen Dzator, Aki Flame, Vincent Jiang, Jianing Kwok, Aaron Lawrence, Kate Meads, Liam Pearce, Pavatharane Sarangadasa, Haylee Shaw, Victor Yu, Elizabeth Crostella, James Wong, Sriya Bobba, Maddison Muller, Yin Chi Hebe Hau, Thomas Wilson, Aleksandra Markovic, Jemma Green, Clara Forbes, Emalee Burrows, Lachlan Hou, Clare O’sullivan, Jonathon Foo, Hannah Greig, A. -J Collins, Callum Chandler, Emily Heaney, Hannah Gross, Monica Morgan, Rebecca Loder, Krishnankutty Rajesh, Shravankrishna Ananthapadmanabhan, Akeedh Razmi, Crystal Vong, Prasanna Pothukuchi, Mary Theophilus, Roshni Sriranjan, Sharon Kaur, Marcelo Kanczuk, Julia De Groot, Angela Corrigan, Damon Li, Danniel Badri, Dominico Ciranni, Elangovan Thaya Needi, Matthew Clanfield, Nicolas Copertino, William Rumble, Maria Kristina Vanguardia, Chen Lew, Rami Dennaoui, Jainil Shah, Joseph Kong, Imogen Koh, Raymond Zeng, Kristian Baziotis-Kalfas, Hannah Denby, Andy Li, Will Tran, Abhinav Singh, Olivia Lin, Michelle Chau, Olivia Donaldson, Christina (Seojung) Min, Shirahn Ballah, Sonia Ching Ting Tsui, Nathania Yong, Lucy Standish, Sarah Tan, Asuka Fujihara, Lily Davies, Ramin Odisho, Anjana Ravi, Josh Collins, Pooja Chandra, Rana Abdelmeguid, Gopal Singh, Xireaili Feierdaiweisi, Dharani Seneviratne, Shambhavi Srivastava, Michelle Yao, Cherilyn Teng, Nebula Chowdhury, Sasini Vidanagama, Charles Lin, Tharushi Sampatha-Waduge, Erica Wang, Chatnapa Yodkitydomying, Julia Silverii, Aaron Lam, Krisha Solanki, Angus Franks, Liam Edwards, Ridvan Atilhan, Rohan Nandurkar, Oliver Wells, Kristina Vanguardia, Dennis King, Elton Edwards, Quang Tran, Seojung Min, Abdul Rauf, Yangzirui Fu, Hodo Haximolla, Mengge Shang, Sharrada Segaran, Shelley Wang, Gananadha Sivakumar, Jaspreet Kaur Sandhu, Neel Mishra, Samantha Hauptman, Alyssa Chua, Danielle Chene, Guy Maddern, Henry Shaw, Qiwen Wang, Siyuan Pang, Christine Lu, James Fung, Kathryn Cyr, Karen Lu, Ming Zhou How, Nelson Hu, Paul Anderson, Philip Jakanovski, Arkan Youssef, Howard Tang, Rory Keenan, Alex Chan, Mitch Canny, Farah Tahir, James Egerton, Justin Yeung, Justin Chan, Lea Tiffany, Michael Bei, Mariolyn Raj, Peter Williams, Sakshar Nagpal, Tim Outhred, Russel Krawitz, Colin Chan-Min Choi, Khadijah Younus, Mary Giurgius, Rosemary Kirk, Amanda Gonzalez Pegorer, Pattarapan Tang-Ieam, Jack Ward, Asanka Wijetunga, Caitlin Zhang, Chris Nahm, Christine Wang, Damian Golja, Gregory Jenkins, Helena Qian, Jason Luong, Kim Nguyen, Sean Suttor, Sherman Lai, Vanessa Ma, Yan Chen, Hoi Hang Yu, Amos Lee, Antonio Barbaro, Cameron Mcguinness, Guy Maddern, Stevie Young, Ye Fang Lim, Georgina Trotta, Phoebe Chao, George Ding, Carol Fang, Andi Lu, Prabhath Wagaarachchi, Charlotte Cornwell, Amy Gojnich, Peter Stewart, Isabella Dong, Kenneth Wong, Luca Burruso, Lucinda Hogan, Nathan Mcorist, Ramnik Singh, Ragavi Jeyamohan, Zhen Hou, William Lai, Emily Taylor, Maria Alejandra Nanez Pantoja, Daniel Mauricio Bolanos Nanez, Gilmer Omar Perez Hernandez, Lia Jasmin Jimenez Ramirez, Mohamed Mohamed, Ahmed Kamal El-Taher, Ahmed Elewa, Mahmoud Ayman Soliman, Menna Diab, Radwa Ali, Ahmed Ahmed, Adham Galal, Ahmed Elkhodary, Ali Alaa, Arwa Faisal, Asmaa Badawy, Donia Eldomiaty, Mohamed Al Sayed, Esraa Rasslan, Mohamed Ramadan, Gamal Elsayed Fares, Hashem Altabbaa, Humam Emad, Muneera Alboridy, Mahmoud Mongy, Osama Albarhomy, Osama Selim, Rawan Rafaei, Raneem Atta, Ahmad Altaweel, Yara Sherif, Youssef Elghoul, Yousef Tarek, Ahmed Abdelfatah Sabry, Ahmad Moustafa, Osama AbouHiekal, Osama Al Shaqran, Zeyad Haggag, Dina Atef, Ahmed Mahmoud, Mahmoud Saad, Mohamed Ragab, Aya Hussien, Mostafa Abdelbaky, Ismail Muhammad, Afnan Morad, Ahmed Ali, Ahmed Hussien, Ahmed Shipa, Ahmed Aboulfotouh, Ahmed Mohamed Hashem, Ahmed Morsi, Alshymaa Ebrahim, Ahmed Mohamed Sayed, Amira Abdelrahman, Aml Ali, Samah Abdelnaeam, Asmaa Emam, Aya Shaban, Fady Barsoum, Esraa Mostafa, Doaa Abdelbaset, Dina Othman, Safaa Othman, Nour Salah Khairallah, Salma Morsi, Armia Azer, Enas Abdelbaset Abdelsamed, Islam Ibrahim, Esraa Abdelbaset, Esraa Hamoda, Fatma Monib, Fatma Harb, Hager Maher, Haitham Mohammed, Kerollos Henes, Kerollos Shamshoon, Mahmoud Hassanein, Magdy Mahdy, Mahmoud Khalil, Manal Ali, Mansour Khalifa, Marwa Amary, Merna Ezz Suliman, Mohammed Saif Al Nasr, Michael Elia, Michael Adly, men Roshdy, Mohammed Al-Quossi, Mohammed Fargaly, Mona Saber, Mostafa Abbas, Ola Haroon, Omima Khalil, Omnia Talaat, Rahma Elnagar, Randa Soliman, Reham Aboelela, Salem Salah, Samia Abdelgawad, Tarek Hussien, George Sobhy, Yasmeen Sayed, Yousra Othman, Reham Silem, Ali Dawood, Tarek Hemaida, Reem Ahmed, Aya Kamal, Mohamed Salah, Ahmed Zaharia, Ebrahim Salem, Osama Fathy Ali Ali Rashed, Mohamed Halawa, Hossam Elfeki, Abdelrahman Mosaad, Abdelrahman Shaaban, Hebatalla Abdelsalam, Ahmed Sakr, Aly Sanad, Amr Elsawy, Bassant Maged Maged, Dana Hegazy, Mohamed Abdelmaksoud, Mahmoud Laymon, Mohamed Taman, Esraa R. Moawad, Hadeer Elsaeed Aboelfarh, Karim Elkenawi, Manar Osama, Mirna Sadek, Mohamed Abdelaziz Elghazy, Mohammed Attiah, Mohamed Nader, Mostafa Shalaby, Omar Attiya, Osama Samir Gaarour, Ahmed Zaghloul, Pola Mikhail, Karim Badr, Hatem Soltan, Mohamed Donia, Mohammed Gaafar, Khaled Abdelwahab, Abdelaziz Sallam, Ahmed Eid, Mohamed Yousri, Omar Hamdy, Aiman Al-Touny, Abdelrhman Alshawadfy, Ahmed Hamdy, Ahmed Ellilly, Ahmed Mahdy, Ahmed El-Sakka, Hamdy Hendawy, Asmaa Salah, Bassma Raslan, Eman Teema, Eslam Albayadi, Esraa Nasser, Hanaa Mohamed, Mohamed Mahmoud, Mostafa Elsaied, Omima Taha, Shaimaa Dahshan, Shimaa Al-Touny, Ahmed Karrar, Ahmed Khairy, Abdelrahman Farag, Asmaa Deafallah, Alaa Mohamed Ads, Rabiaa Alomar, Issa AbuShawareb, Abdallah Saeed, Abdelhafeez Mashaal, Adel Mohamed Ads, Sohila Ghanem, Ahmed Elghamry, Eman Ayman Nada, Youssef Ali Noureldin, Mohamed Fayez Fouda, Nourhan Shaheen, Shereen Allam, Ibrahim Mazrou, Ali Fahmy Shehab, Wesam Kussaili, Dimitrios Korkolis, Evangelos Fradelos, Aikaterini Sarafi, Nikolaos Machairas, Konstantinos S. Giannakopoulos, Fotios Stavratis, Georgios Korovesis, Gerasimos Tsourouflis, Myrto D. Keramida, Nikolaos Kydonakis, Stylianos Kykalos, Athanasios Syllaios, Panagiotis Dorovinis, Dimitrios Schizas, Orestis Ioannidis, Anastasia Malliora, Elissavet Anestiadou, Konstantinos Zapsalis, Fotios Kontidis, Lydia Loutzidou, Nikolaos Ouzounidis, Stefanos Bitsianis, Savvas Symeonidis, Smaragda Skalidou, Olga Maria Valaroutsou, Themistoklis Dagklis, Alexandra Arvanitaki, Apostolos Mamopoulos, Apostolos Athanasiadis, Stergios Kopatsaris, Ioannis Kalogiannidis, Ioannis Tsakiridis, Georgios Kapetanios, Evangelos Papanikolaou, Nikolaos Tsakiridis, Fotios Zachomitros, Andreas Larentzakis, Argyrios Gyftopoulos, Konstantinos Albanopoulos, Apostolos Champipis, Christos Yiannakopoulos, Gavriella Zoi Vrakopoulou, Konstantinos Saliaris, Konstantinos Lathouras, Spyridon Skoufias, Georgia Doulami, Metaxia Bareka, Eleni Arnaoutoglou, Fragkiskos Angelis, Fragkiskos Angeslis, Michael Hantes, Maria Ntalouka, Maytham A. Al-Juaifari, Mohammed Alwash, Rasool Maala, Yasir Adnan Zwain, Sara Ahmed Saleh, Mohammed Khorsheed, Antonio Pesce, Carlo V. Feo, Massimiliano Bernabei, Francesca Petrarulo, Nicolò Fabbri, Raffaele Labriola, Silvia Jasmine Barbara, Simone Bosi, Angela Romano, Anna Canavese, Caterina Catalioto, Claudio Isopi, Cristina Larotonda, Gerti Dajti, Matteo Rottoli, Iris Shari Russo, Stefano Cardelli, Francesco Castagnini, Francesco Traina, Giulia Guizzardi, Giulia Giuzzardi, Mara Gorgone, Marco Maestri, Pasquale Cianci, Ivana Conversano, Enrico Restini, Domenico Gattulli, Giorgia Grillea, Marco Varesano, Giacomo Calini, Adelaide Andriani, Davide Gattesco, Giovanni Terrosu, Mattia Zambon, Pietro Matucci Cerinic, Luisa Moretti, Davide Muschitiello, Samantha Polo, Vittorio Bresadola, Salah Abu Wardeh, Mahmoud Al-Baw, Saif Alhaleeq, Subhi Al-Issawi, Abdalqader Al Smadi, Esmat Alsaify, Farah Banihani, Noor Massadeh, Nada Massadeh, Dima Al-issawi, Basel Elyan, Qotadah Al-Shami, Yazan Alomari, Abed Alazeez Alkhatib, Bader Alzghoul, Ahmad Saleh, Jamal Yaghmour, Mahmoud Shahin, Mohammed Maali, Dawood Alatefi, Heba Al-Smirat, Abdulhakim Hezam, Nassar Alathameen, Amr Al Hammoud, Abdulrahim Al Kaddah, Salem Ayasrah, Hamza Abuuqteish, Tesneem Al-Mwajeh, Reena Makableh, Saad Bataineh, Amin Shabaneh, Wesam Alnatsheh, Marwan Aldeges, Huda Hamad, Sireen Shehahda, Dima Khassawneh, Osama Alzyoud, Risan Alrosan, Hasan Awad, Tariq Khaldoon, Rabab Shannaq, Mohammad Al hamoud, Bader Abo fadalah, ath Al-Hazaimeh, Wail Khraise, Lara Alnajjar, Majjd Alnajjar, Sohaib Al-Omary, Adnan Ababneh, Alaa Albashaireh, Mohammad Khadrawi, Mohammad Aljamal, Tayseer Athamneh, Ro-a Muqbel, Maryam Al-jammal, Ahmad Masarrat, Alia Al-zawaydeh, Ibrahim Taha, Taima’ Qattawi, Rayyan Smadi, Ayah Alhaleem, Mosab Alboon, Omar Hazaymeh, Leen Karasneh, Safa’ Al-Haek, Marin Almahroush, Tamam Alfrijat, Aya Elporgay, Hadeel Shanag, Hamza Agilla, Hind Alameen, Marya Bensalem, Mawadda Altair, Malak Ghemmied, Rehab Alarabi, Sara Alhudhairy, Rima Gweder, Amal Alzarroug, Ebtihal Alabed, Fadwa Elreaid, Omar A. Elkharaz, Fatma Fathi Elreaid, Safa Sasi Albatni, Haitham Elmehdawi, Milad Gahwagi, Ayman Mohamed, Tariq Alfrjani, Khaled Khafifi, Ayat Rasheed, Ayoub Akwaisah, Hassan Bushaala, Mustafa Elfadli, Mohamed Moftah, Salima Algabbasi, Salma Esaiti, Sara Elfallah, Abtisam Alharam, Fatima Alariby, Mohamed Isweesi, Tarik Ahmed Eldarat, Ayman Arhuma Dabas, Akram Alkaseek, Ahmed Mohammed Abodina, Aya Alqaarh, Hibah Bileid Bakeer, Hoda Salem Alhaddad, Husein Aboudlal, Sawsan Alsaih, Noora Abubaker, Najwa Abdelrahim, Ali Alzarga, Basma Omar, Farah Faris, Qamrah Alhadad, Asma Abufanas, Hussameddin Badi, Israa Benismai, Hawa Obeid, Abdulwahab Abdalei, Ahmed Abdulrahman, Aisha Swalem, Ebtisam Alzarouq, Amna Safar, Esra Shagroun, Boshra Hashem, Fatheia Elrishi, Fatima Abdulali, Habeeba Ahmed, Ibrahim Eltaib, Joma Elzoubia, Aisha Albarki, Hoda El Mugassabi, Fatima Abushaala, Amany Abuzaho, Nida Juha, Raneem Egzait, Sundes Shetwan, Alzahra Lemhaishi, Faisel Matoug, Eman Abdulwahed, Aamal Askar, Abir Ben Ashur, Adel Bezweek, Bushra Altughar, David Emhimmed, Donia Elferis, Laila Elgherwi, Enas Soula, Doaa Gidiem, Maren Grada, Khawla Derwish, Maram Alameen, Nassib Algatanesh, Ahlam Elkheshebi, Reem Ghmagh, Sharf Barka, Sultan Ahmeed, Sarah Aljamal, Zahra Alragig, Mohamed Addalla, Ahmed Atia, Atab Kharim, Fathia Mahmoud, Muhannud Binnawara, Entisar Alshareea, Mohamed Alsori, Aisha Alshawesh, Ghaliya Mohamed H. Alrifae, Amira Ashour, Anwaar Abozid, Asil Omar Saleh Alflite, Anwar Mohamed, Jaber Arebi, Fatma Alagelli, Hana Yousef Gineeb, Rawia Ghmagh, Rihab Mohammed Bin Omar, Retaj Alaqoubi, Sara Mohammed, Serien Hossain Bensalem, Tahani Elgadi, Wesam Sami, Yara Bariun, Abdulhadi Mohammed Alhadi Alhashimi, Dheba Almukhtar Abdulla, Heba Rhuma, Husam Enaami, Asraa Ali Alboueishi, Hayat Ben Hasan, Mohamd A. A. Alkchr, Bashir Albakosh, Najah Alsari, Mahammed Aldreawi, Najat Ben Hasan, Khaled Abushanab, Rawad Yahya, Narimantas Samalavicius, Vitalijus Eismontas, Jonas Jurgaitis, Oleg Aliosin, Vitalija Nutautiene, Andee Dzulkarnaen Zakaria, Anil Kumar Sree Kumar Pillai, Dinesh Kumar Vadioaloo, Mohamed Ashraf Mohamed Daud, Jien Yen Soh, Mohd Zaim Zakaria, Siti Mayuha Rusli, Nur Ayuni Khirul Ashar, Zatul Akmar Ahmad, Afiq Aizat Ramlee, Sharifah Nor Amirah Syed Abdul Latiff Alsagoff, Ahmad Anuar Sofian, Muhammad Badrul Hisyam Mohamad Jamil, Bahiyah Abdullah, Mohamad Faiz Noorman, Muhammad Fihmi Zainal Abidin, Mohamed Izzad Isahak, Siti Nasyirah Nisya Adnan, Zaidatul Husna Mohamad Noor, Luis Adrian Alvarez-Lozada, Alejandro Quiroga Garza, Andrea Aguilar Leal, Bernardo Alfonso Fernández Reyes, Ethel Valeria Orta Guerra, Francisco Javier Arrambide Garza, Héctor Erasmo Alcocer Mey, Jorge Arath Rosales Isais, Juventino Tadeo Guerrero Zertuche, Patricia Ludivina González García, Luis Antonio Heredia Sánchez, Marcela Patricia Flores Mercado, Oscar Alonso Verduzco Sierra, Pedro Emiliano Ramos Morales, Stephie Oyervides Fuentes, Víctor Manuel Peña Martínez, Yesika Alejandra Guerra-Juárez, Ana Karina Flores-González, Surya Singh, Arwa Hadi, Christian Woodbridge, David Thornton-Hume, Jack Forsythe, Isini Dharmaratne, Vivian Pai, John Windsor, Kamran Zargar, Lucy Waldin, Lily Winthrop, Matias Alvarez, Meileen Huang, Matt Kumove, Marta Simonetti, Namisha Chand, Oliver Goldsmith, Oscar Guo, Paul Monk, Karen Zhou, Sai Harshitha Penneru, Shaamnil Prasad, Seifei Ren, Terrence Hill, Vyoma Mistry, Selena Sun, Ashley Pereira, Scott Mclaughlin, Andrew Stokes, Avinash Sathiyaseelan, Jeremy Rossaak, Janice Lim, Kenya Brooke, Liam Quinlan, Mark Pottier, Nayanika Podder, Puja Jinu, Shanay Ramphal, Wikus Vermeulen, Flavio Ordones, Fraser Jeffery, Ibrahim S. Al Busaidi, Janelle Divinagracia, William Ju, Yizhuo Liu, Tamara Glyn, Nasya Thompson, Vivien Graziadei, Joshua Canton, Joseph Furey, Horim Choi, Grace Coomber, Tanya Divekar, Tessa English, Erin Gernhoefer, Tom Healy, Justin Chou, Dikshya Parajuli, Catherine Reed, Rod Studd, Anthony Lin, Cameron Wells, Cindy Xu, Arwa Hadi, Andrew Maccormick, Heejun Park, Athulya Rathnayake, Brittany Williams, Ashley Chan, Corinne Smith, Francesca Casciola, Jainey Bhikha, Jonathan Luo, Kevin Yi, Megan Singhal, Ria George, Rosie Luo, Taylor Frost, Fatima Hakak, Akhita George, Angela Carlos, Annie Ho, Connor Mcrae, Jonathan Lescheid, Jenny Soek, Andrew Pham, Sophie St Clair, Su-Ann Yee, Jennifer Lim, Chun-Yen Wu, Taehoon Kim, Anne Qi Chua, Christopher Harmston, Hamish Boyes, Holly Cook, Jamie Struthers, Jess Radovanovich, Nicholas Quek, Chekodi Fearnley-Fitzgerald, Deborah Wright, Kushan Ghandi, Natalie Matheson, Matthew James McGuinness, Brian Chen, Rebecca Indiana Douglas, Konrad Richter, Nisha Bianca Soliman, Scott Matthew Bolam, Vineeth Vimalan, William Currie, Mitchell Cuthbert, Poppy Ross, Amy Nicholson, Briar Garton, Emilie Agnew, Niamh Conlon, Nicholas Waaka, Ritwik Kejriwal, Sean Nguyen, Edmund Leung, Milidu Ratnayake, Quintin Smith, Nejo Joseph, Bosco Yue, Calvin Fraser, Charles Lam, Ethan Figgitt, Gordon Liu, Kevin Tan, Ha Seong You, Helen Zheng, Jenny Luo, James Sharp, Kabir Khanna, Levi Simiona, Michel Luo, Patrick Wong, Rebecca Luu, Rohit Paul, Shiva Nair, Shadie Asadyari-Lupo, Wing Hung, Geoffrey Ying, Jess Ho, Alan Wu, Eamon Walsh, Jouyee Lee, Jessie Liu, Sunny Yao, Omar Nosseir, Jennifer Dang, Simon Young, Sof’ya Zyul’korneeva, Theresa Boyd, Jess Ho, Alan Wu, Sunny Yao, Abdullahi Musa Kirfi, Adamu Bala Ningi, Mohammad Albuhari Garba, Makama Baje Salihu, Ohia Ernest Ukwuoma, Abdullahi Ibrahim, Isa Mienda Sajo, Muhammad Baffah Aminu, Liman Haruna Usman, Oloko Nasirudeen Lanre, Ibrahim Shaphat Shuaibu, Stephen Yusuf, Tiamiyu Ismail, Gabi Ibrahim Umar, Ademola Adeyeye, Ehis Afeikhena, Favour Chinenye Nnaji, Joy Onyekachi Agu, Temiloluwa Peace Maxwell, Oluwatosin Olakunle Motajo, Oghenekaro Ifoto, Seubong-Abasi Imoh Okon, Jerry Godfrey Makama, Amina Abosede Mohammed-Durosinlorun, Bashiru Aminu, Polite Iwedike Onwuhafua, Caleb Mohammed, Lubabatu Abdulrasheed, Joel Amwe Adze, Khadijah Richifa Suleiman, Lydia Regina Airede, Mathew Chum Taingson, Stephen Bodam Bature, Stephen Akau Kache, Uchechukwu Ohijie Ogbonna, Mohammed Bello Fufore, Abdulkarim Iya, Adeshina A. Ajulo, Ahmad Mahmud, Bilal Shuaibu Yahya, Farida Onimisi-Yusuf, Hope Isaac, Timothy Jawa, Fashe Joseph, Bemi Kala, Maisaratu A. Bakari, David Wujika Ngwan, Abubakar umar, Abraham L. Filikus, Daniel Wycliff, Abiodun Okunlola, Olukayode Abiola, Adebayo Adeniyi, Olabisi Adeyemo, Babatunde Awoyinka, Olakunle Babalola, Adewumi Bakare, Taiwo Buari, Cecilia Okunlola, Gbadebo Adeleye, Adedayo Salawu, Henry Abiyere, Adetolu Ogidi, Tesleem Orewole, Habiba Ibrahim Abdullahi, Godwin Akaba, Arome Achem, Asi-oqua Bassey, Emeka Ayogu, Bilal Sulaiman, Dennis Anthony Isah, Chukwunonso Nnamdi Akpamgbo, Felicia Asudo, Nathaniel Adewole, Omachoko Oguche, Peter Ejembi, Samuel Ali Sani, Paul Chimezie Andrew, AliyuYabagi Isah, Bolarinwa Eniola, Zumnan Songden, Teddy Agida, Terkaa Atim, Taofiq Olayinka Mohammed, Hadijat Olaide Raji, Femi Ibiyemi, Hafeez Salawu, Olushola Fasiku, Remi Sanyaolu Solagbade, Mariam Motunrayo Shiru, Gbadebo Hakeem Ibraheem, Justina Oruade, Grace Ezeoke, Tabish Chawla, Aliya Begum Aziz, Anoosha Marium, Ayesha Akbar Waheed, Faiqa Binte Aamir, Faiza Qureshi, M. Hammad Ather, Iqra Fatima Munawar Ali, Izza Tahir, Maha Ghulam Akbar, Ronika Devi Ukrani, Sajjan Raja, Sehar Salim Virani, Shahryar Noordin, Saif Ur Rehman, Shalni Golani, Syed Roohan Aamir, Syed Musa Mufarrih, Usama Waqar, Maliha Taufiq, Ahmed Siddique Ammar, Adya Ejaz, Albash Sarwar, Ahmed Usman Khalid, Shehrbano Khattak, Aliza Imran, Omer Bin Khalid, Urauba Kaleem, Urwah Muneer, Yumna Kashaf, Fatima Zafar, Adil Zaheer, Muhammad Ali, Amna Shafaat, Arisha Qazi, Asjad Imran, Mahnoor Tariq, Muhammad Nadeem Aslam, Shehroz Ali, Tabish Atiq, Tayyiba Wasim, Daniyal Babar, Ahmad Zain, Muhammad Ibtisam, Uzair Ahmed, Syed Talha Bin Aqeel, Muhammad Muhib, Muhammad Anas Abbal, Nasar Ahmad Khan, Imran Javed, Layth Alkaraja, Dana Amro, Ghaida Manasrah, Ibraheem Hammouri, Ihab Abu Hilail, Jihad Zalloum, Laith Alamlih, Mahmoud Nasereddin, Munia Rajabi, Sa’ed Shalalfeh, Zeinab Natsheh, Khamis Elessi, Mustafa Abu Jayyab, Mohammed Astal, Mosheer Al-Dahdouh, Alaa Eddin Salameh, Alaa Ayyad, Nimatee Dawod, Hamza Alsaid, Iyas Matar, Majd Hassan, Mohammed Bakeer, Mohammad Malasah, Shehab Abuhashem, Mohammed Salem, Sorinel Lunca, Mihail Gabriel Dimofte, Stefan Morarasu, Ana Maria Musina, Cristian Ene Roata, Natalia Velenciuc, Aleksandr Butyrskii, Maxim Bozhko, Amet Ametov, Sharfuddin Chowdhury, Doaa Bagazi, Julio Domenech, Alejandro Rosello-Añon, Ana Monis, Caterina Chiappe, Beatriz Cuneo, Pablo Clemente-Navarro, Jorge Febre, Jorge Sanz-Romera, Marcos Lopez-Vega, Ignacio Miranda, Rocio Valverde-Vazquez, Sara Garcia, Maria Jose Sanguesa, Zutoia Balciscueta, Enrique Ruiz, Eduardo Marco, Elena Talavera, Joan Farre, Loreto Bacariza, Mireia Duart, Violeta Ureña, Xenia Carre, Hytham K. S. Hamid, Montasir A. Abd-Albain, Sami Galal-Eldin, Monira Sarih, Eithar Adam, Samir Ismail, Malaz Azhari, Tawfieg Hassan, Mohamed Salaheldein, Zainab Abdalla, Wahiba Ahmed, Monzer Abdulatif Mohamed Alhassan, Hozifa Mohamed Abdalla Suliman, Hozifa Mohamed Bdalla Suliman, Rogia Ahmed Abdalla Ahmed, Enas Mohammedtom Abdulhameed Babekir, Munya Ali Talab Khairy, Maha Mukhtar Ahmed Mukhtar, Rzan Ali Hamedelneel Ali, Yasir Babkir Ali Al-Shambaty, Fatima Imad Yousif, Hawa Mohammed Hassan Mohammed, Lana Osher, Menhag Abdelbast, Mohamed Yassin, Noon Moawia, Rowa Abdalsadeg, Abrar Husein, Baraa Elhassan, Alnazeer Y. Abdelbagi, Mohammed A. Adam, Eithar M. Ali, Ibrahim A. B. Mohammed, Maab Mohamed, Mohamed Abdulaziz, Mazin Akasha, Muaz Hassan, Nadir Hilal, Noon Abdalla Abdelrahman Mohamed, Noora Abubaker, Omeralfarouk Mohammed, Shakir Mohamed, Walaa Osman, Fatima Mustafa, Alaa A. Salih, Doua Ali, Doha Mohammed Ahmed Almakki, Hanan Elnour Mohamed, Abdelhadi Elmubark, Mohamed Hassan, Ammar Alnour, Amna Elaagib, Ayman Abdelrahman, Mubarak Abdelkhalig, Khalid Nour Eldaim, Afra Babiker, Entisar Ahmed, Maab Ali, Eman Hussain, Mansour Wedatalla, Alaaaldeen Ahmed, Alla Aldeen Hamza, Mohab Mohammed, Omer Osman, Reham Ibrahim, Rihab Ahmed, Ruaa Ahmed, Ruaa Yasir, Safaa Awadallah, Sara Mohmmed, Suhaib Hassan, Walid Shaban, Aisha Hussein, Reem Rafea, Ahmed Abdalla, Abdalla Ahmed, Khalid Mohamed, Mansour Mohammed, Mohamed Altahir, Mohammed Adam, Omer Mohamed, Walaa Abdullah, Hammad Fadlalmola, Ahmed Yassir Abdalla, Ahmed Ali Omer, Ahmed Alfatih Mustafa, Rawan Elnoman Elhadi, Essam Eldien Abuobaida Banaga, Fatima Osman, Mohamed Galal Ali Abdalla, Hala Abdelhalim Mohamed Taha, Noon Ezzeldien Abdalmahmoud, Rofuida Hussien Nafie, Sami Jamal, Sharwany Ahmed, Doha Amir AtaAlmanan, Rawan Alsheikh Ali, Abdallah Aladna, Abdullah Aljoumaa, Hamdi Nawfal, Salma Jamali, Fatima Khouja, Ammar Niazi, Al Rawashdeh, Nahla Kechiche, Mouna Gara, Mouna Nasr, Marwen Baccar, Oumayma Benamor, Sawssen Chakroun, Ahmet Necati Sanli, Ahmet Yildiz, Mehmet Ali Demirkiran, Yildiz Buyukdereli Atadag, Yusuf Iskender Tandogan, Esin Ozkan, Yıldırım Ozer, Muhammed Miran Oncel, Senad Kalkan, Tolga Gover, Berke Manoglu, Ilayda Oksak, Ipek Kurt, Kerem Rifaioglu, Selman Sokmen, Tayfun Bisgin, Yasemin Yildirim, Abdil Yetkin Keskin, Tugce Dogan, Berfin İlgaz Sahin, Cemil Aydin, Duygu Ece Benek, Hale Nur Tiras, Mert Arslangilay, Mert Aslangilay, Muhammet Yaytokgil, Mehmet Ali Capar, Yasemin Yazgan, Sebnem Bektas, Ahmet Can Alagoz, Alara Ece Dagsali, Aylin Izgis, Kadir Uzel, Mustafa Soytas, Niyazi Cakir, Abdullah Emre Askin, Ibrahim Azboy, Kubilay Sabuncu, Merve Aslan, Melek Sahin, Mustafa Oncel, Nuri Okkabaz, Ramazan Kemal Sivrikaya, Alparslan Saylar, Dr. Alparslan Saylar, Meltem Yasar, Ergin Erginoz, Haktan Ovul Bozkir, Kagan Zengin, Mehmet Faik Ozcelik, Server Sezgin Uludag, Zeynep Ozdemir, Osman Sibic, Hatice Telci, Mehmet Abdussamet Bozkurt, Yasin Kara, Mustafa Deniz Tepe, Adnan Gündoğdu, Bilge Akın, Dilan Pehlivan, Ali Guner, Duygu Baysallar, Berkay Yıldız, Hale Cepe, Murat Emre Reis, Ayse Nilufer Yuzgec, Nurtac Kıralı, Taha Anıl Kodalak, Mehmet Ulusahin, Kamar Selim, Ahmet Kale, Mehmet Emre Gecici, Melis Ozbilen, Zeynep Düzyol, Aylin Gemici, Elzem Korkmaz, Eminenur Şen, Muhammed Enes Taşcı, Elifsu Camkıran, Güşta Elieyioğlu, İkbal Kayabaş, Tevfik Kıvılcım Uprak, Canan Aral, Ayten Saraçoğlu, Mustafa Ümit Uğurlu, Zeynep Hazal Baltacı, Ege Nur Akkaya, Cem Fergar, Elif Zeynep Tabak, Guldane Zehra Kocyigit, Ilgaz Kayilioglu, Süleyman Polat, Elif Çolak, Mehmet Emin Kara, Mert Candan, Mustafa Safa Uyanık, Ahmet Can Sarı, Attila Ulkucu, Alperen Taha Certel, Arzu Dindar, Beyza Durdu, Cigdem Bayram, Eslem Kaya, Hakan Akdere, Ibrahim Ethem Cakcak, Ikranur Yavuz, Mert Omur, Mirac Ajredini, Erhan Onur Aydoğdu, Eylül Şenödeyici, Ulku Ceren Koksoy, Baturay Kansu Kazbek, Deniz Serim Korkmaz, Dogancan Yavuz, Hakan Yilmaz, Zeynep Sahan Cetınkaya, Elif Durmus, Filiz Tuzuner, Furkan Hokelekli, Mucahid Mutlu, Seyma Orcan Akbuz, Ziya Can Kus, Michael Farrell, Alayna Craig-Lucas, Matthew Painter, Ashley Titan, Aditya Narayan, Bunmi Fariyike, Lisa Knowlton, Tiffany Yue, Emily Benham, Abdelrahman Nimeri, Hope Werenski, Nicole Kaiser, Caroline Reinke

**Affiliations:** 1 Department of Surgery, University of Auckland, Auckland, New Zealand; 2 Department of Surgery, Mayo Clinic, Rochester, MN USA; 3 Department of Surgery, University of Newcastle, Newcastle, NSW Australia; 4 Australian National University, Canberra, ACT Australia; 5 University of Adelaide, Adelaide, SA Australia; 6 Monash University, Melbourne, VIC Australia; 7 Northern Sydney Local Health District, Sydney, NSW Australia; 8 Department of Clinical Surgery, University of Edinburgh, Edinburgh, Scotland; 9 Centre for Medical Informatics, Usher Institute, University of Edinburgh, Edinburgh, Scotland; 10 Department of Surgical Sciences, University of Otago, Dunedin, New Zealand; 11 Dokuz Eylul University Hospital, Izmir, Turkey; 12 Istanbul University Cerrahpasa—Cerrahpasa School of Medicine, Istanbul, Turkey; 13 Karadeniz Technical University, Trabzon, Turkey; 14 Faculty of Medicine, Bezmialem Vakif University, Istanbul, Turkey; 15 King Abdullah University Hospital, Irbid, Jordan; 16 Kanuni Sultan Suleyman Training and Research Hospital, Istanbul, Turkey; 17 Faculty of Medicine, Çorum Research and Training Hospital, Hitit University, Çorum, Turkey; 18 Bunbury Hospital, Bunbury, WA Australia; 19 Kartal Dr. Lutfi Kirdar Training and Research Hospital, Istanbul, Turkey; 20 Northern Beaches Hospital, Sydney, NSW Australia; 21 Samsun Training and Research Hospital, Samsun, Turkey; 22 Hospital Lorenzo Bonomo, Andria, Italy; 23 New Plymouth Hospital, New Plymouth, New Zealand; 24 Palestine Medical Complex, Ramallah, Palestine; 25 Afe Babalola University Multisystem Hospital, Ado Ekiti, Nigeria; 26 The Alfred Hospital, Melbourne, VIC Australia; 27 Barau Dikko Teaching Hospital, Kaduna, Nigeria; 28 Ballarat Base Hospital, Ballarat, VIC Australia; 29 Gosford Hospital, Gosford, NSW Australia; 30 Nilratan Sircar Medical College and Hospital, Kolkata, India; 31 Istanbul Medipol University Hospital, Istanbul, Turkey; 32 Emergency Municipal Hospital No. 6, Moscow, Russia; 33 Aleppo University Hospital, Aleppo, Syria; 34 Latrobe Regional Hospital, Melbourne, VIC Australia; 35 El Tadamon Specialised Hospital, Port Said, Egypt; 36 Wellington Hospital, Wellington, New Zealand; 37 Suez Canal University Hospital, Ismailia, Egypt; 38 Hospital Sant Joan Reus, Tarragona, Spain; 39 St John of God Midland Public and Private Hospital, Perth, WA Australia; 40 New South Wales Local Health District Site Bega Hospital, Bega, NSW Australia; 41 Royal Australasian College of Surgeons, Sydney, NSW Australia; 42 Federal Teaching Hospital, Ido Ekiti, Nigeria; 43 School of Medicine, Marmara University, Istanbul, Turkey; 44 Karadeniz Technical University Farabi Hospital, Trabzon, Turkey; 45 Armidale Rural Referral Hospital, Armidale, NSW Australia; 46 CHU Saadna Abdennour Hospital, Sétif, Algeria; 47 Sydney University, Sydney, NSW Australia; 48 Afe Babalola University Multisystem Hospital, Ado-Ekiti, Nigeria; 49 Autonomous University of Nuevo León, Monterrey, Mexico; 50 George Papanikolaou Hospital, Thessaloniki, Greece; 51 Hospital Universiti Teknologi MARA (HUiTM), Puncak Alam, Selangor Malaysia; 52 University of Tasmania, Hobart, TAS Australia; 53 Center Anti Cancer, Sétif, Algeria; 54 Albury Wodonga Health, Albury, NSW Australia; 55 Armadale Health Service, Perth, WA Australia; 56 Austin Hospital, Melbourne, VIC Australia; 57 Gold Coast University Hospital, Gold Coast, QLD Australia; 58 Goulburn Base Hospital, Goulburn, NSW Australia; 59 Logan Hospital, Logan, QLD Australia; 60 Manning Base Hospital, Taree, NSW Australia; 61 Maroondah Hospital, Melbourne, VIC Australia; 62 Monash Medical Centre, Melbourne, VIC Australia; 63 Orange Base Hospital, Orange, NSW Australia; 64 Women’s and Children’s Hospital, Adelaide, SA Australia; 65 Alexandria Main University Hospital, Alexandria, Egypt; 66 General Hospital of Thessaloniki “George Papanikolaou”, Thessaloniki, Greece; 67 Iaso, Athens, Greece; 68 Larissa University Hospital, Larissa, Greece; 69 Al-Najaf Al-Ashraf Teaching Hospital, Najaf, Iraq; 70 IRCCS Istituto Ortopedico Rizzoli, Bologna, Italy; 71 Alkarak Governmental Hospital, Al-Karak, Jordan; 72 Middlemore Hospital, Auckland, New Zealand; 73 Rotorua Hospital, Rotorua, New Zealand; 74 Dunedin Hospital, Dunedin, New Zealand; 75 North Shore Hospital, Auckland, New Zealand; 76 Abubakar Tafawa Balewa University Teaching Hospital Bauchi, Bauchi, Nigeria; 77 Modibbo Adama University Teaching Hospital, Yola, Nigeria; 78 University of Abuja Teaching Hospital, Gwagwalada, Nigeria; 79 Creek General Hospital, Karachi, Pakistan; 80 Christchurch Hospital, Christchurch, New Zealand; 81 Services Hospital Lahore, Lahore, Pakistan; 82 Nasser Medical Complex, Gaza, Palestine; 83 King Saud Medical City, Riyadh, Saudi Arabia; 84 Hospital Arnau De Vilanova, Valencia, Spain; 85 Al-Moalem Medical City, Khartoum, Sudan; 86 Alandalus Clinic, Elduiem, Sudan; 87 Mugla Training and Research Hospital, Mugla, Turkey; 88 Faculty of Medicine, Trakya University, Edirne, Turkey; 89 Ufuk Üni̇versi̇tesi̇ Tip Fakültesi̇ Dr.Ri̇dvan Ege Sağlik Araştirma Uygulama Merkezi̇ Hastanesi̇, Ankara, Turkey; 90 Lehigh Valley Health Network, Allentown, PA USA; 91 Stanford Health Care, Palo Alto, CA USA; 92 Griffith University, Gold Coast, QLD Australia; 93 Hunter New England Network, Newcastle, NSW Australia; 94 University of Health Sciences, Tepecik Training and Research Hospital, Izmir, Turkey; 95 Texas Tech University Health Sciences Center, Lubbock, TX USA; 96 Central Gippsland Health, Sale, VIC Australia; 97 Azienda Unità Sanitaria Locale di Ferrara-University of Ferrara, Ferrara, Italy; 98 University Hospital of Udine, Udine, Italy; 99 Whangarei Base Hospital, Whangarei, New Zealand; 100 Hebron Government Hospital, Hebron, Palestine; 101 Regional Institute of Oncology Iasi, Iasi, Romania; 102 University of Edinburgh, Edinburgh, UK; 103 Ipswich Hospital, Ipswich, QLD Australia; 104 John Hunter Hospital, Newcastle, NSW Australia; 105 Queen Elizabeth Ii Jubilee Hospital, Brisbane, QLD Australia; 106 The Royal Melbourne Hospital, Melbourne, VIC Australia; 107 General Hospital of Athens “Laiko”, Athens, Greece; 108 Melbourne Training Circuit, Melbourne, VIC Australia; 109 King Edward Medical University Hospital, Lahore, Pakistan; 110 The Canberra Hospital, Canberra, ACT Australia; 111 The Queen Elizabeth Hospital, Adelaide, SA Australia; 112 Royal Prince Alfred Hospital, Sydney, NSW Australia; 113 National Ribat University Hospital, Khartoum, Sudan; 114 Deakin University, Melbourne, VIC Australia; 115 University of Tripoli, Tripoli, Libya; 116 Counties Manukau Health, Auckland, New Zealand; 117 Oncology Center Mansoura University, Mansoura, Egypt; 118 Waikato Hospital, Hamilton, New Zealand; 119 University of Newcastle, Newcastle, NSW Australia; 120 Flinders University, Adelaide, SA Australia; 121 Royal Perth Hospital, Perth, WA Australia; 122 Lyell Mcewin Hospital, Adelaide, SA Australia; 123 Western Health, Melbourne, VIC Australia; 124 Al Basheer Hospital, Amman, Jordan; 125 Education Karima Hospital, Merowe, Sudan; 126 Elduiem Teaching Hospital, Elduiem, Sudan; 127 Atrium Health, Charlotte, NC USA; 128 Agios Savvas Anticancer Hospital of Athens, Athens, Greece; 129 Aga Khan University Hospital, Karachi, Pakistan; 130 Sunshine Coast University Hospital, Birtinya, QLD Australia; 131 Ibrahim Malik Teaching Hospital, Khartoum, Sudan; 132 Abu Saleem Trauma Hospital, Tripoli, Libya; 133 Alkhadra Hospital, Tripoli, Libya; 134 Benghazi Medical Center, Benghazi, Libya; 135 Gharyan Central Hospital, Gharyan, Libya; 136 Ibn Sina Teaching Hospital, Sirte, Libya; 137 Misurata Central Hospital, Misurata, Libya; 138 Tripoli Central Hospital, Tripoli, Libya; 139 Tripoli Medical Center/Tripoli University Hospital, Tripoli, Libya; 140 Zliten Medical Centre, Zliten, Libya; 141 Klaipeda University Hospital, Klaipeda City Municipality, Lithuania; 142 School of Medical Sciences & Hospital USM, Universiti Sains Malaysia, Kota Bharu, Malaysia; 143 University Hospital Dr. Jose Eleuterio Gonzalez, Monterrey, Nuevo Leon Mexico; 144 Hospital Susana Lopez De Velencia, Popayán, Cauca Colombia; 145 Al Istiklal Hospital, Amman, Jordan; 146 Geraldton Regional Hospital, Geraldton, WA Australia; 147 University Hospital Fattouma Bourguiba, University of Monastir, Monastir, Tunisia; 148 Abdulkadir Yuksel State Hospital, Gaziantep, Turkey; 149 University of Otago, Dunedin, New Zealand; 150 The First Affiliated Hospital of Jinan University, Guangzhou, China; 151 Box Hill Hospital, Melbourne, VIC Australia; 152 Bahria International Hospital Lahore, Lahore, Pakistan; 153 Ibn-Sina Hospital, Khartoum, Sudan; 154 Northern Hospital, Melbourne, VIC Australia; 155 Auckland City Hospital, Auckland, New Zealand; 156 Invercargill (Kew) Hospital, Invercargill, New Zealand; 157 Waitakere Hospital, Auckland, New Zealand; 158 Frankston Hospital, Melbourne, VIC Australia; 159 Tanta University Hospital, Tanta, Egypt; 160 Sir Charles Gairdner Hospital, Perth, WA Australia; 161 Western Sydney University, Sydney, NSW Australia; 162 Perth Metro Training Circuit, Perth, WA Australia; 163 Alexandria University, Alexandria, Egypt; 164 Casey Hospital, Melbourne, VIC Australia; 165 Flinders Medical Centre, Adelaide, SA Australia; 166 Hedland Health Campus, Port Hedland, WA Australia; 167 Westmead Hospital, Sydney, NSW Australia; 168 Whyalla Hospital & Health Services, Whyalla, SA Australia; 169 Menofia University Hospital, Shibin el Kom, Egypt; 170 Tauranga Hospital, Tauranga, New Zealand; 171 Royal Australasian College of Surgeons, Adelaide, SA Australia; 172 Khartoum North Teaching Hospital (Bahri Hospital), Khartoum, Sudan; 173 University of Auckland, Auckland, New Zealand; 174 University of Western Australia, Perth, WA Australia; 175 Campbelltown Hospital, Sydney, NSW Australia; 176 Royal Adelaide Hospital, Adelaide, SA Australia; 177 Wyong Public Hospital, Wyong, NSW Australia; 178 Al Tayseer Hospital, Zagazig, Egypt; 179 Assiut University Hospital, Assiut, Egypt; 180 Aswan University Hospital, Aswan, Egypt; 181 Mansoura University Hospital, Mansoura, Egypt; 182 IRCCS Azienda Ospedaliero-Universitaria di Bologna, Bologna, Italy; 183 Princess Basma Teaching Hospital, Irbid, Jordan; 184 University of Ilorin Teaching Hospital, Ilorin, Nigeria; 185 Bashair Teaching Hospital, Khartoum, Sudan; 186 Gadarif Teaching Hospital, Gadarif City, Sudan; 187 Hippokratio General Hospital of Thessaloniki, Thessaloniki, Greece; 188 United Hospital, Karachi, Pakistan

**Keywords:** Pain management, Predictive markers

## Abstract

Opioids are frequently overprescribed after surgery. We applied a tabular foundation model to predict the risk of post-discharge opioid consumption. The model was trained and internally validated on an 80:20 training/test split of the ‘Opioid PrEscRiptions and usage After Surgery’ (ACTRN12621001451897p) study cohort, including adult patients undergoing general, orthopaedic, gynaecological and urological operations (*n* = 4267), with external validation in a distinct cohort of patients discharged after general surgical procedures (*n* = 826). The area under the receiver operator curve was 0.84 (95% confidence interval [CI] 0.81–0.88) at internal testing and 0.77 (95% CI 0.74–0.80) at external validation. Brier scores were 0.13 (95% CI 0.12–0.14) and 0.19 (95% CI 0.17–0.2). Patients with a <50% predicted risk of opioid consumption consumed a median of 0 oral morphine equivalents in the first week after surgery. Applying this model would reduce opioid prescriptions by 4.5% globally, and counterfactual modelling suggests without increasing time in severe pain (−4.3%, 95% CI −17.7 to 8.6).

## Introduction

Excess opioid prescribing at surgical discharge is a significant contributor to the global opioid crisis^1^. Inappropriate opioid prescribing is associated with higher rates of persistent opioid use and opioid-related deaths^2^. Whilst opioids are prescribed with the aim of reducing postoperative pain and discomfort to optimise recovery, accumulating evidence shows that the harms frequently outweigh the benefits^3–5^. Even among those who do not consume their prescribed opioids, diversion of unused opioids into the community poses serious risks of exacerbating the opioid epidemic^6^.

Opioids are frequently overprescribed at hospital discharge following surgery, with quantities larger than typically consumed by patients^5,7,8^. The ‘Opioid PrEscRiptions and usage After Surgery’ (OPERAS) study found that patients are prescribed more than double the quantity of opioids they would consume in the first week after surgery. It is also acknowledged that analgesic prescribing is complex, with multifactorial contributors driving clinician and patient decision making. Therefore, personalised, data-driven strategies are needed to guide opioid prescribing in the post-discharge setting, tailored to patients’ needs. However, accurately identifying these needs requires reliable measures of patient-reported opioid consumption after discharge^8^.

The OPERAS study enroled a large, prospective cohort of surgical patients across a range of specialties with patient-reported, post-discharge opioid consumption data^9^. This offers an opportunity to reduce the quantity of unused opioid prescriptions based on robust, patient-reported opioid consumption data. We therefore aimed to develop and validate a model to predict opioid consumption within the first week after surgical discharge and assess the potential clinical impact of applying such a model in this context on patient-reported outcomes.

## Results

Between 4 April 2022 and 4 September 2022, data from 4267 patients across 144 hospitals in 25 countries were collected and analysed (2268 women, 53.2%; median age 50 years; median length of stay 2 [IQR 1–3] days). Patients were followed up at a median of 7 days (IQR 7–8). Overall, 1308 (31%) were discharged with an opioid prescription, but only 1014 (23.8%) consumed an opioid within 7 days post-discharge; 297 (22.7% of those prescribed an opioid) did not consume any opioid. Only 3 (0.1%) not prescribed an opioid consumed an opioid during this time.

### Predictors of opioid consumption

Consumption of opioids after discharge occurred more frequently after discharge in patients undergoing orthopaedic surgery or emergency surgery; when patients consumed higher quantities of opioids 24 h prior to discharge; when patients used opioids in the 6 months prior to admission; or were from a high-income country (Table 1). Age, gender, smoking status, urgency of surgery, the surgery type, discharge day opioid use, and 6-month opioid use prior to admission, were all considered and were available in both datasets, known to plausibly influence opioid consumption, and were included in our a priori protocol. Feature importance was then compared, including all candidate variables (Supplementary Figs. 1 and 2 and Supplementary Table 3) to guide feature selection. The variables alcohol use and BMI category had >10% missing data and were excluded^10^. Based on the mean dropout loss of the RMSE, ASA category and indication for surgery were excluded. Our final predictors used for model training were therefore age, gender, smoking status, urgency of surgery, procedure mapped to BUPA severity categories, discharge day opioid use, and prior 6-month opioid use (Supplementary Table S3).

**Table 1:** Demographics and candidate variables across datasets, by consumption of opioids post-discharge from surgery

		Overall OPERAS Cohort (*n* = 4267)				Newcastle External Validation Cohort (*n* = 826)			
		Did not consume opioid	Consumed opioid	Total	*p*	Did not consume opioid	Consumed opioid	Total	*p*
Total *N* (%)		3253 (76.2)	1014 (23.8)	4267		542 (65.6)	284 (34.4)	826	
Age	Median (IQR)	49.0 (34.0 to 64.0)	52.0 (36.0 to 65.0)	50.0 (34.0 to 64.0)	0.029	57.0 (37.2 to 70.0)	44.0 (30.8 to 58.2)	52.5 (34.0 to 67.0)	<0.001
Gender	Female	1728 (53.1)	540 (53.3)	2268 (53.2)	0.969	242 (44.6)	172 (60.6)	414 (50.1)	<0.001
	Male	1525 (46.9)	474 (46.7)	1999 (46.8)		300 (55.4)	112 (39.4)	412 (49.9)	
	(Missing)	0 (0.0)	0 (0.0)	0 (0.0)		0 (0.0)	0 (0.0)	0 (0.0)	
Alcohol	Heavy (11+)	30 (0.9)	42 (4.1)	72 (1.7)	<0.001				
	Light (1–5)	472 (14.5)	272 (26.8)	744 (17.4)					
	Moderate (6–10)	84 (2.6)	57 (5.6)	141 (3.3)					
	Non-drinker (0)	2281 (70.1)	436 (43.0)	2717 (63.7)					
	(Missing)	386 (11.9)	207 (20.4)	593 (13.9)					
BMI	Normal (18.5 –4.9)	908 (27.9)	253 (25.0)	1161 (27.2)	<0.001				
	Obese (31–40)	677 (20.8)	256 (25.2)	933 (21.9)					
	Overweight (25–30)	1160 (35.7)	302 (29.8)	1462 (34.3)					
	Severely obese (>40)	128 (3.9)	77 (7.6)	205 (4.8)					
	Underweight (<18.5)	61 (1.9)	8 (0.8)	69 (1.6)					
	(Missing)	319 (9.8)	118 (11.6)	437 (10.2)					
Smoking	No	2425 (74.5)	797 (78.6)	3222 (75.5)	0.01	484 (89.3)	229 (80.6)	713 (86.3)	0.001
	Yes	828 (25.5)	217 (21.4)	1045 (24.5)		58 (10.7)	55 (19.4)	113 (13.7)	
	(Missing)	0 (0.0)	0 (0.0)	0 (0.0)		0 (0.0)	0 (0.0)	0 (0.0)	
Procedure	ACL repair	61 (1.9)	17 (1.7)	78 (1.8)	<0.001				0.002
	Appendicectomy	574 (17.6)	187 (18.4)	761 (17.8)		138 (25.5)	67 (23.6)	205 (24.8)	
	Cholecystectomy	962 (29.6)	263 (25.9)	1225 (28.7)		169 (31.2)	125 (44.0)	294 (35.6)	
	Colorectal resection	299 (9.2)	94 (9.3)	393 (9.2)		72 (13.3)	28 (9.9)	100 (12.1)	
	Cystectomy	29 (0.9)	3 (0.3)	32 (0.7)					
	Hip arthroplasty	131 (4.0)	70 (6.9)	201 (4.7)					
	Hysterectomy	246 (7.6)	41 (4.0)	287 (6.7)					
	Inguinal hernia repair	474 (14.6)	102 (10.1)	576 (13.5)		107 (19.7)	38 (13.4)	145 (17.6)	
	Knee arthroplasty	125 (3.8)	131 (12.9)	256 (6.0)					
	Nephrectomy	72 (2.2)	28 (2.8)	100 (2.3)					
	Nissen fundoplication	26 (0.8)	2 (0.2)	28 (0.7)					
	Oophorectomy and Salpingectomy	29 (0.9)	12 (1.2)	41 (1.0)					
	Oophorectomy only	22 (0.7)	2 (0.2)	24 (0.6)					
	Prostatectomy	77 (2.4)	24 (2.4)	101 (2.4)					
	Rotator cuff repair	12 (0.4)	10 (1.0)	22 (0.5)					
	Salpingectomy only	35 (1.1)	6 (0.6)	41 (1.0)					
	Shoulder arthroplasty	10 (0.3)	9 (0.9)	19 (0.4)					
	Shoulder labral repair	13 (0.4)	0 (0.0)	13 (0.3)					
	Sleeve gastrectomy	56 (1.7)	13 (1.3)	69 (1.6)					
	Thyroidectomy	0 (0.0)	0 (0.0)	0 (0.0)		56 (10.3)	26 (9.2)	82 (9.9)	
	(Missing)	0 (0.0)	0 (0.0)	0 (0.0)					
ASA	I–II	2776 (85.3)	828 (81.7)	3604 (84.5)	0.007				
	III–V	474 (14.6)	184 (18.1)	658 (15.4)					
	(Missing)	3 (0.1)	2 (0.2)	5 (0.1)					
Indication	Benign	2848 (87.5)	892 (88.0)	3740 (87.6)	0.765				
	Malignancy	405 (12.5)	122 (12.0)	527 (12.4)					
	(Missing)	0 (0.0)	0 (0.0)	0 (0.0)					
Urgency	Elective	2280 (70.1)	633 (62.4)	2913 (68.3)	<0.001	284 (52.4)	146 (51.4)	430 (52.1)	0.9
	Emergency	973 (29.9)	381 (37.6)	1354 (31.7)		255 (47.0)	135 (47.5)	390 (47.2)	
	(Missing)	0 (0.0)	0 (0.0)	0 (0.0)		3 (0.6)	3 (1.1)	6 (0.7)	
Total OME consumed 24-h pre-discharge	Median (IQR)	0.0 (0.0 to 30.0)	40.0 (15.0 to 75.0)	9.0 (0.0 to 40.0)	<0.001	0.0 (0.0 to 15.0)	22.5 (15.0 to 45.0)	7.5 (0.0 to 22.5)	<0.001
Pre-admission opioid use	No	3186 (97.9)	897 (88.5)	4083 (95.7)	<0.001	537 (99.1)	269 (94.7)	806 (97.6)	<0.001
	Yes	67 (2.1)	117 (11.5)	184 (4.3)		5 (0.9)	15 (5.3)	20 (2.4)	
	(Missing)	0 (0.0)	0 (0.0)	0 (0.0)		0 (0.0)	0 (0.0)	0 (0.0)	
Country Income Level	HIC	1115 (34.3)	804 (79.3)	1919 (45.0)	<0.001	542 (65.6)	284 (34.4)	826	
	LMIC	2138 (65.7)	210 (20.7)	2348 (55.0)					
	(Missing)	0 (0.0)	0 (0.0)	0 (0.0)					

In a multivariable model of the whole OPERAS cohort, in reference to appendicectomy, we found ACL repair surgery (odds ratios (OR) 7.61, 95% confidence interval (CI) 3.00–19.3; *p* < 0.001), cholecystectomy (OR 1.54, 95% CI 1.04–2.28; *p* = 0.031), hip arthroplasty (OR 4.20, 95% CI 2.19–8.09; *p* < 0.001), inguinal hernia repair (OR 2.78, 95% CI 1.67–4.64; *p* < 0.001), knee arthroplasty (OR 13.74, 95% CI 7.26–26.0; *p* < 0.001), oophorectomy and salpingectomy (OR 3.14, 95% CI 1.11–8.87; *p* = 0.031), rotator cuff repair (OR 21.15, 95% CI 6.15–72.67; *p* < 0.001), and shoulder arthroplasty (OR 22.83, 95% CI 4.29–121.57; *p* < 0.001) were associated with higher risks of opioid consumption. Malignant indication, day of discharge opioid use quantity, and prior 6-month opioid use were also significant predictors (Supplementary Fig. 3).

### Model development and validation

**Fig. 1:**
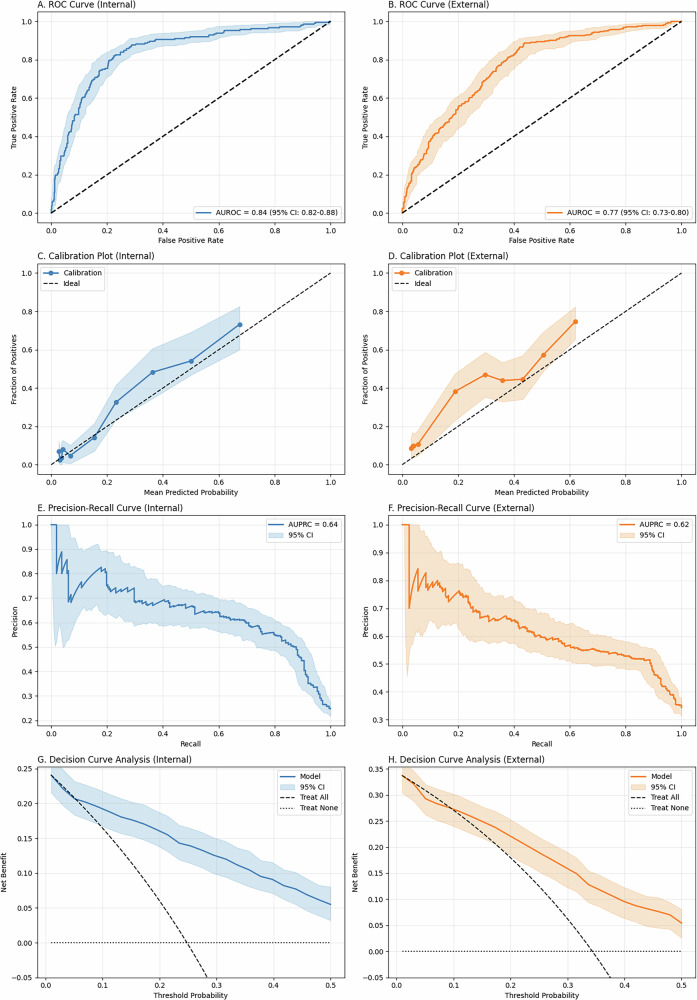
Model predictive performance plots. **A**, **B** Receiver operator curve (ROC), **C**, **D** calibration plot, **E**, **F** precision-recall (PR) curve, and **G**, **H** decision curve analysis of our model during internal (blue) and external (orange) validation. The shaded area represents 95% confidence intervals.

Use of a model with all candidate predictors and a refined model with the final set of predictors were compared in ROC and PR curves (with AUROC and AUPRC metrics), as reported in Supplementary Fig. 4 and Table 2. Results of internal validation in the OPERAS testing cohort and external validation in the Newcastle cohort are shown in Fig. 1 and Table 2. The internal validation AUROC was 0.84 (95% CI 0.81–0.88), and the external validation AUROC was 0.77 (95% CI 0.74–0.80), showing excellent and good discrimination, respectively. Brier scores were 0.13 (95% CI 0.12–0.14) and 0.19 (95% CI 0.17–0.20), respectively, indicating good calibration, with a tendency toward under-predicting the need for an opioid when visually assessing calibration plots. AUPRC was 0.64 (95% CI 0.57–0.72) and 0.62 (95% CI 0.56–0.68). Sensitivity, specificity, positive and negative predictive values across different risk thresholds are summarised in Table 3. Sensitivity cross-validation analysis, excluding individual procedures, is reported in Supplementary Results.

**Table 2:** Performance metrics of models in the derivation cohort, calculated using 10-fold cross-validation

Cohort	Model	Area under the receiver operator curve	Area under the precision-recall curve	Brier score
Internal	All predictors	0.86 95% CI: 0.84–0.89	0.64 95% CI: 0.57–0.71	0.13 95% CI: 0.11–0.14
Internal	Refined predictors	0.84 95% CI: 0.81–0.88	0.64 95% CI: 0.57–0.72	0.13 95% CI: 0.12–0.14
External	Refined predictors	0.77 95% CI: 0.74–0.80	0.62 95% CI: 0.56–0.68	0.19 95% CI: 0.17–0.20

**Table 3:** Proportion of patients that consumed an opioid stratified by model predictions across varying risk classification thresholds, tabulated with accompanying sensitivity, specificity, positive predictive value (PPV), and negative predictive value (NPV)

Cut-off value	Low risk rate (%)	High risk rate (%)	Sensitivity (%)	Specificity (%)	PPV (%)	NPV (%)
10	4.8 (*n* = 20/421)	44.3 (*n* = 192/433)	90.6	62.5	44.3	95.2
20	7.1 (*n* = 37/521)	52.6 (*n* = 175/333)	82.5	75.4	52.6	92.9
30	10.3 (*n* = 62/604)	60.0 (*n* = 150/250)	70.8	84.4	60	89.7
40	13.5 (*n* = 90/665)	64.6 (*n* = 122/189)	57.5	89.6	64.6	86.5
50	16.9 (*n* = 122/721)	67.7 (*n* = 90/133)	42.5	93.3	67.7	83.1
60	20.5 (*n* = 161/784)	72.9 (*n* = 51/70)	24.1	97	72.9	79.5
70	23.3 (*n* = 193/828)	73.1 (*n* = 19/26)	9	98.9	73.1	76.7

### Clinical implications

Taking the ground truth as a patient consuming opioids, in the OPERAS testing set, the sensitivity (99.2%) of clinician prescribing is expectedly high as very few patients consume an opioid when not prescribed it, but their specificity of 89.2% is associated with a false positive rate of 10.8%. In the external Newcastle cohort, the sensitivity of clinician prescribing was 100% and the specificity was 91.5% with a false positive rate of 8.5%. Using the model could improve specificity relative to clinicians by targeting opioid prescriptions to individuals with >50% risk of consuming them (see Table 3: specificity of 93.3 and false positive rate of 6.7%). Setting the classification threshold at 50%, 15.6% were predicted to require an opioid, compared to 31.9% that were prescribed opioids. In the external Newcastle cohort, 15.9% were predicted to require an opioid versus the 40.1% of the cohort that were prescribed opioids (see example cases in Table 4).

**Table 4:** Table of five example patients, their contributory predictor variables with their model-derived predicted risk of consuming an opioid in the first week after surgical discharge, alongside the prediction classification at a 50% threshold, the real-world clinician decision and real-world patient consumption outcome

Age	Gender	Smoking status	Procedure	BUPA category	Urgency	Discharge day OME used on the day of discharge	Prior 6-month opioid use	Total OME consumed after discharge	Predicted risk of consuming opioids	Binary prediction	Clinician decision	Patient consumption
62	Female	No	Knee arthroplasty	MAJOR	Elective	65	Yes	334.6	0.906	Yes	Yes	Yes
21	Male	No	Appendicectomy	INTER	Emergency	25	No	80	0.514	Yes	Yes	Yes
24	Male	No	Appendicectomy	INTER	Elective	0	No	0	0.024	No	No	No
77	Male	No	Inguinal hernia repair	MINOR	Elective	40	No	26	0.2	No	Yes	Yes
58	Female	No	Colorectal resection	MAJOR	Elective	0.4	No	0	0.078	No	No	No

**Fig. 2:**
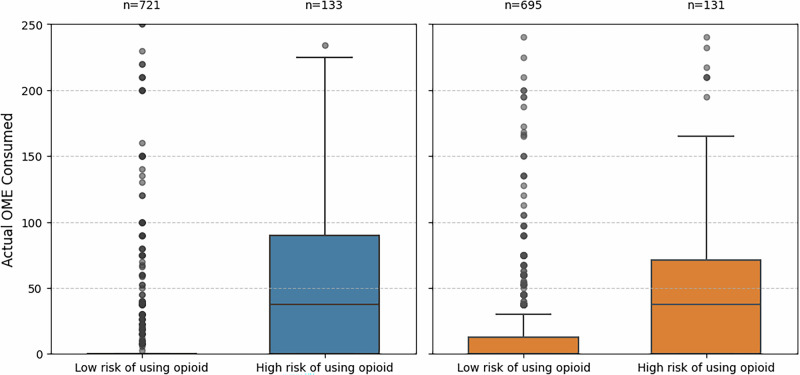
Box plots depicting total milligram morphine equivalents consumed stratified by patients predicted to be <50% and >50% risk of consuming an opioid in the first week after surgical discharge. Left: internal validation (blue); right: external validation (orange). The box represents the interquartile range (IQR), and the whiskers extend to 1.5× the IQR, with outlier dots plotted beyond the whisker extents.

To assess harm, we looked at the quantity of opioids consumed in the proportion of patients predicted not to require an opioid by the algorithm but who had consumed opioids after discharge (i.e. false negatives). Among those predicted to require an opioid in the OPERAS testing dataset, the median quantity of opioids consumed was 37.5 OME (IQR 0.0–90.0; mean 83.4 ± 172.0) compared to a median of 0.0 OME (IQR 0.0–0.0; mean 15.4 ± 52.3; *p* < 0.0001) among those predicted not to require an opioid (122 [16.9%] of whom consumed some opioid). Similarly, among those predicted to require an opioid in the Newcastle external cohort, the median quantity of opioids consumed was 37.5 OME (IQR 0.0–71.2; mean 48.8 ± 61.3) compared to a median of 0.0 OME (IQR 0.0–12.5; mean 15.4 ± 36.4; *p* < 0.0001) among those predicted not to require an opioid (196 [28.2%] of whom consumed some opioid; Fig. 2). Further, applying the TabPFN regression model to predict OME quantities consumed in the week after surgery; the median predicted quantity was 11.0 (IQR 1–25; range 0.7–56; mean 13.5 ± 13.3), which can be taken to mean that generally prescribing more than 10 OME per day in the first week after surgery is likely in excess to requirements (Supplementary Fig. 5).

We then calculated the observed effect of prescribing opioids on quality of life, proportion of time spent in severe pain, and mean satisfaction with analgesia, and then formulated a counterfactual, i.e. quality of life, pain severity, and satisfaction when the model prediction was discordant with the clinician’s prescribing decision. Taking the difference between the observed and counterfactual effects provides an estimate of the impact of these errors (i.e. discordant prescribing decisions) on these outcomes. There were no differences in patient satisfaction (+0.6, 95% CI −0.19 to 1.5; *p* = 0.08), proportion of time spent in severe pain (−4.3%, 95% CI −17.7 to 8.6; *p* = 0.7) or quality of life (−0.03, 95% CI −0.12 to 0.06; *p* = 0.8) with the use of our model compared to the real-world opioid prescribing in the OPERAS testing set.

### Global impact

**Fig. 3:**
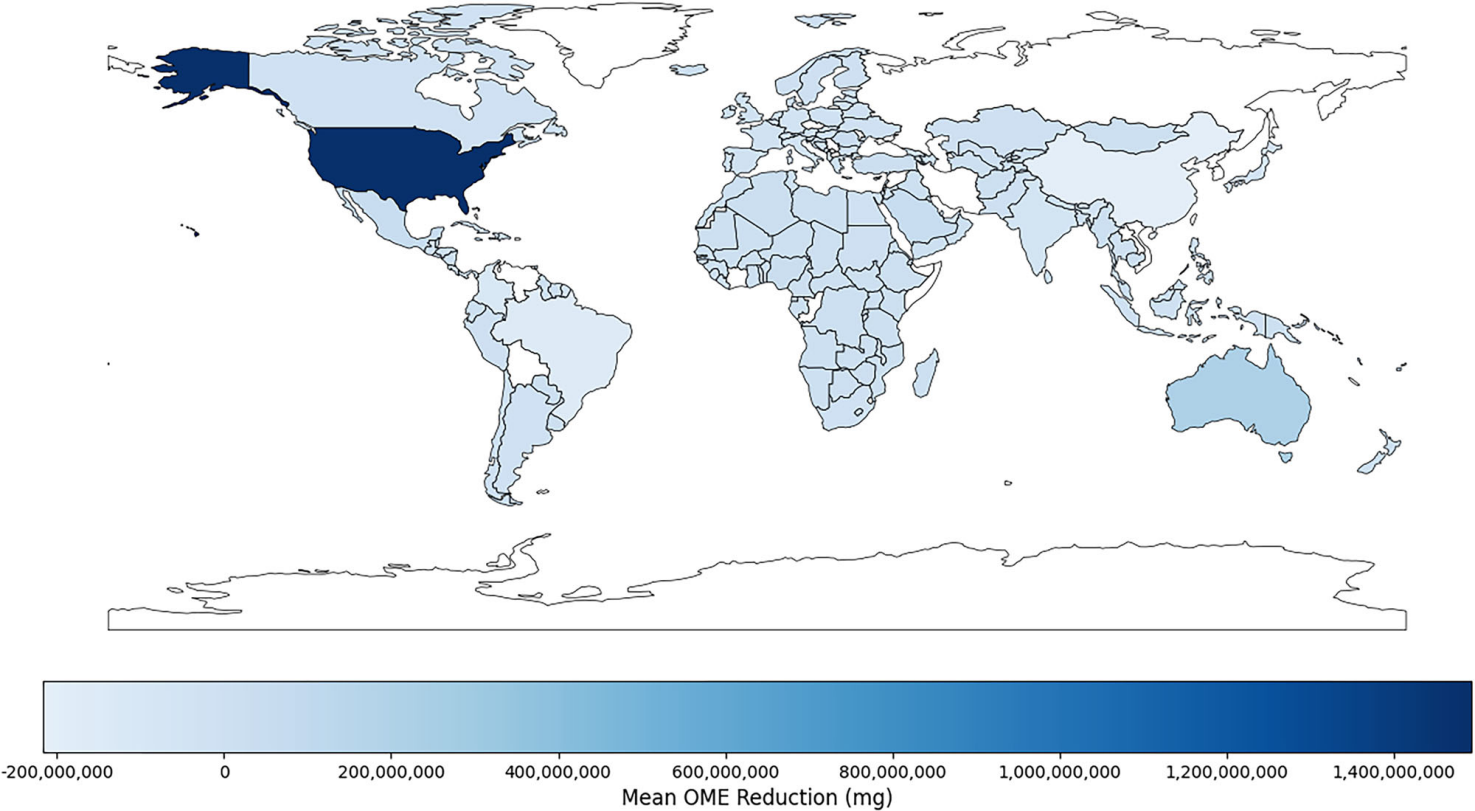
Global opioid reduction modelling. Modelled global reductions in average oral morphine equivalents (OMEs) prescribed based on country-level case volume and predicted reductions in OMEs. Darker shades of blue indicate greater reductions in OME prescribed, while countries with missing data are shown in white.

Applying our predictions from this model to surgical case volumes globally, this personalised prescribing approach would result in 12,129,908 fewer prescriptions (22.5% reduction in opioid prescribing rate), and a 4.5% decrease in overall quantities of oral morphine equivalents (OMEs) prescribed translating to an average 667,596,123 (SD 4,405,314,710) fewer OMEs globally (Fig. 3).

## Discussion

We have developed and validated an algorithm to predict the need for an opioid prescription at surgical discharge. Derived from a multispecialty, global dataset, this model generates generalisable and specific predictions across a diverse range of surgical patients. Our model offers guidance to support a personalised approach to analgesic prescribing that may facilitate reductions in overall opioid prescribing after surgery, particularly when they are unlikely to be used (reducing false positive prescriptions by >20%). Further counterfactual simulations suggest that this overall reduction in predicted opioid prescriptions could be achieved without negatively impacting patient-reported quality of life, satisfaction with analgesia, and pain severity. Additionally, when opioids were predicted not to be required where patients had actually consumed some in the real world, quantities consumed averaged less than 20 OME (approximately two 10 mg oral morphine tablets), such that opioid omission would have been safe.

Guidelines advocate for a personalised approach to opioid prescribing at surgical discharge^11,12^, yet little data is available to guide the decision to prescribe an opioid^13^. This work expands on recent work that has highlighted the large discrepancy between what is prescribed after surgery and what is actually consumed by patients^8^. Coupled with rising awareness of the harms of opioid overprescription, and possibly overstated analgesic benefits in the post-discharge setting^5,14^, there is an urgent need for a reliable method to curtail opioid overprescription. A web application that allows input of variables for the final predictors and reports the model’s predicted risk of patients consuming an opioid in the first week after surgical discharge can be accessed here: https://operas-model.shinyapps.io/OPERAS/.

Several rule-based, procedure-specific, or protocolised approaches to opioid prescribing exist. Some examples include specialty-specific standardisations^15–17^, while others stratify prescriptions based on inpatient opioid consumption^18–20^. Our data are congruent in finding inpatient opioid consumption to be an important predictor of analgesic needs based on patient-reported post-discharge opioid consumption. This work also advances on recent efforts using deep learning to predict opioid refills, offering improved discrimination in a broader surgical and geographic cohort^21^.

Importantly, broad rule-based approaches to opioid prescribing can inadvertently lead to protocolised overprescription^22^. This highlights the need for individualised prescribing, hence, the value of a predictive model in place of a one-size-fits-all approach. Additionally, administrative datasets and dispensing data may not accurately reflect patients’ opioid consumption. Hence, our patient-reported consumption data, robustly obtained through standardised telephone interviews, offers a more accurate parallel to patients’ need for an opioid after surgery^8,9^. Hence, this work is a substantial advance to previous single-centre and specialty-specific studies by offering personalised recommendations that incorporate patient-, procedure-, and pathology-specific predictors.

While this study does not explore long-term outcomes of implementing this algorithm in practice, our modelling is in agreement with previous literature, which shows that reducing opioid prescribing in a landscape where overprescription is so common can occur without negatively impacting patient recovery^23^. Our algorithm predicted a reduction in opioid prescribing by up to 73% without a significant predicted detriment in pain-satisfaction, quality of life, or pain-severity. Others have also shown that implementing protocols to reduce postsurgical opioid prescribing could significantly reduce chronic opioid use without negatively impacting patient recovery^24,25^. In further support, a recent meta-analysis of randomised evidence suggests the omission of opioids, particularly in the setting of minor surgery, was frequently associated with equivalent analgesia and fewer opioid-related adverse events^14^.

This work has significant implications for postoperative opioid stewardship globally. Our modelling estimates substantial reductions in the quantities of opioids that would be prescribed based on extrapolating the model-predicted versus observed differences in opioid prescribing. This is likely to have particular value in high-income countries where excess opioid prescribing is more common^26^, and this study supports that these personalised reductions can be achieved without detriment to patients’ quality of pain relief^5,14^.

A key limitation of the present study is that patients’ consumption patterns can be influenced by what they are prescribed by doctors^8^. Hence, a residual risk of overestimating opioid requirements persists in the absence of randomised evidence to offer counterfactual data. However, as it is well known that clinicians prescribe in excess of what patients consume^8^, and therefore, offering a predictive tool that is likely to result in overall reductions in opioid prescribing whilst better matching patients’ consumption needs within the first week after discharge remains attractive. We also did not include some variables that were found to be important prognostically (such as alcohol use and BMI), this was due to the high missing data rates that may introduce bias^10^, and also indicate challenges in using these parameters in routine use. A further limitation is that whilst the OPERAS study included a range of surgical specialties and procedures, this list was not comprehensive, and extrapolation of these findings to different surgeries and diverse cohorts should be cautioned. Additionally, our external validation cohort had a more restrictive set of included procedures that lacked orthopaedic operations. Further external validation of this model would therefore be of value.

We envision this tool being used on the day of discharge by the discharging clinician in collaboration with patients and the wider clinical team. Algorithmic clinical decision making should not occur in isolation. Our algorithm, which considers individual patient factors, offers advantages compared to ‘one-size-fits-all’ protocols, to enable both judicious and personalised opioid prescribing. Such an algorithm, however, does not obviate the need for clinical judgement, consideration of patient circumstances and preferences, safe prescription quantities, and appropriate disposal advice. Prospective evaluation is now required ahead of widespread clinical implementation.

In conclusion, we have developed and validated a novel foundation model for predicting the consumption of opioid analgesia after surgical discharge. This tool is derived from a global cohort and is underpinned by patient-reported opioid consumption data, offering a generalisable tool for surgeons globally. Future studies should prospectively investigate the clinical impacts of utilising our model in practice.

## Methods

### Study design

This was an international, prospective, multicentre, observational cohort study. Analyses were based on a prespecified, published protocol, and the study was registered in the Australian New Zealand Clinical Trials Registry in October 2021 (ANZCTR: ACTRN12621001451897p)^9^. This is a secondary analysis of the OPERAS dataset, which addresses the per protocol, a priori aim of addressing risk factors for opioid consumption at 7 days; we extend this work here through development and validation of a prognostic model using a pre-trained foundation model to aid clinical decision making regarding who should be prescribed opioids on the day of discharge following surgery. This study is reported in accordance with the TRIPOD+AI guidelines^27^.

### Ethical approval

The Hunter New England Human Research Ethics Committee approved both the OPERAS study (2021/ETH11508) and data collection for the external validation cohort from Newcastle, Australia (2021/ETH11508). For the OPERAS study, institutional approvals were also obtained according to the requirements at each participating centre and verified by the central steering committee.

### Development cohort

In the OPERAS dataset, all hospitals routinely performing general, orthopaedic, gynaecological and urological procedures were eligible to enrol. Data were collected prospectively whilst patients were in hospital from clinical records and discharge prescriptions, and post-discharge via standardised telephone interviews at 7 days^28^. Data collection took place over six predefined 14-day data collection periods between 4 April 2022 and 4 September 2022. Centres could choose to participate in any number of 14-day consecutive recruitment periods.

### External validation cohort

An independent prospective cohort of patients aged 18 years or older undergoing elective or emergency general surgical procedures across two metropolitan hospitals in Newcastle, Australia, between March 2019 and March 2020 was used for external validation^28^. This cohort included patients undergoing thyroidectomies, which were not included in the OPERAS study. Prescribing was per the discharging clinician’s discretion.

### Eligibility criteria

Centres participating in the OPERAS study prospectively screened and approached all consecutive patients who met eligibility criteria to obtain informed participant consent where this was a requirement of the site ethics approval (Supplementary Table 1). Participants could withdraw at any stage. Consecutive adult patients aged 18 years or older undergoing either elective (planned) or emergency (unplanned) common general surgical (cholecystectomy, appendicectomy, inguinal hernia repair, colon resection, fundoplication, or sleeve gastrectomy), orthopaedic (total or reverse shoulder arthroplasty, rotator cuff or labral repair, anterior cruciate ligament repair, or hip or knee arthroplasty), gynaecological (hysterectomy, oophorectomy, or salpingectomy and oophorectomy), and urological procedures (prostatectomy, cystectomy or nephrectomy) were eligible to be included^9^. Only patients discharged home or to a non-healthcare setting were included. Patients receiving medication-assisted treatment of opioid dependence with methadone, suboxone, or buprenorphine, discharged to rehabilitation, nursing-supported care services, another hospital, or discharged with palliative intent were excluded. Patients undergoing multivisceral resections or who required return to theatre were also excluded from the OPERAS study.

### Outcome variable

The dependent variable for model development was the binary variable of whether patients had consumed any OMEs after discharge from the hospital or not. We pragmatically chose this outcome for prognostic modelling to avoid undue harms possibly associated with predicting excess quantity of opioids, owing to the skewed distribution of both quantitative opioid prescribing and consumption patterns as overviewed in our sentinel analysis^7^. Individuals predicted not to consume an opioid within 7 days were considered a target group in which opioids could be safely omitted. An exploratory regression analysis using the same model architecture applied to our primary categorical outcome was also performed.

### Explanatory variables

Data were also collected on patient demographics (age, gender, tobacco use, vaping status, alcohol use, BMI), Society of Anesthesiologists (ASA) physical status classification, comorbidities, diagnosis and procedure-specific details (indication, surgical approach, and urgency), opioid use in the 6 months prior to admission (6-month opioid use), opioid quantity consumed in the 24 h prior to hospital discharge (discharge day opioid use), opioid prescription quantity at the time of discharge from hospital (including opioid type, dose, and quantity of pills). Procedures were mapped to BUPA severity classification codes (minor, intermediate, major)^29^. Data on opioid doses were converted to OMEs to account for the potencies of different medications and allow comparison. OME conversion ratios were calculated using conversion ratios defined by the Australian and New Zealand College of Anaesthetics (ANZCA) Faculty of Pain Medicine^30^. Where opioid conversion ratios were not defined by ANZCA, accepted conversion ratios were identified through a literature review^9^. Methods for the calculation of OMEs are further detailed in Supplementary Note 1 and Supplementary Table 2. Patient satisfaction with analgesia was measured on a numeric rating scale between 0 (lowest satisfaction) and 100 (highest satisfaction).

### Data analysis

All statistical analyses were performed with R version 4.2.0 (R Foundation for Statistical Computing, Vienna, Austria) and Python version 3.9.6 (Python Software Foundation, Amsterdam, Netherlands). An a priori sample size calculation was performed, necessitating a minimum sample size of 852 for a multivariate linear regression model^9^. For a binary classification model, targeting an AUC of 0.8, with 12 parameters and 30% prevalence of opioid prescribing, a minimum sample size of 413 (with 124 events, events per predictor 10.32)^31^.

Descriptive statistics were used to compare demographic and prescription-specific variables based on whether or not patients were prescribed opioids at discharge using the *χ*^2^ test for categorical variables and the Kruskal-Wallis test for continuous variables. Data are presented as median (IQR) or mean ± SD. A two-tailed α level was set at 5% for interpretation of significance. Missing data were explored via visual inspection. For descriptive purposes, a mixed effects binomial logistic regression model, including individual procedures and country as the random effect, was produced and presented with associated OR and 95% CIs.

For feature selection, we utilised the descriptive machine learning explanations (*DALEX*) package in R, enabling model agnostic variable importance quantification across various model types^32^. This framework was employed to assess variable importance via a permutation-based approach. This was supplemented by the use of Shapley Additive Explanations.

Models were trained with a state-of-the-art tabular foundation model (TabPFN)^33^, a new model that is well suited to smaller (<10,000 sample) datasets, capable of handling missing data, and outperforms best-in-class machine learning approaches such as gradient boosting (comparison to logistic regression, and hyperparameter optimised, random forest, gradient boosted and neural network models are reported in Supplementary Tables 3 and 4). TabPFN is a generative transformer-based foundation model tailored for prediction tasks trained on tabular data by itself, having been pre-trained on millions of synthetic datasets, including various patterns of missing data. Regarding data preprocessing, we first converted categorical variables to numeric, and missing data were retained. Two TabPFN models were subsequently trained to classify the likelihood of opioid consumption in the first week after surgical discharge, in an 80% subset of the original OPERAS cohort. The first model contained all candidate predictors, and the second model only contained the refined subset of predictors determined by the feature selection approaches described above. Cross-validation with a leave-one-out approach for each procedure was performed as a sensitivity analysis when using BUPA severity classifications in place of the procedure. Receiver operator curves (ROC) and precision-recall (PR) curves, with the respective area under the receiver operator curve (AUROC) and average under the precision-recall curve (AUPRC) metrics, were interrogated to compare models. Thereafter, the results of the refined model in the testing and external Newcastle cohorts were presented. ROC, PR curve, calibration plots, and decision curve analysis plots were plotted with bootstrapped 95% CIs from 100 draws, and presented alongside their respective AUROC, AUPRC, and Brier score metrics (initially, using the >0.5 risk threshold for positive classification). AUC values between 0.5–0.59 were considered ‘poor’, 0.6–0.69 ‘moderate’, 0.7-0.79 ‘good’ and ≥0.8 ‘excellent’ discrimination^34^.

To assess clinical utility and impact, we first compared the quantity of opioids consumed by patients in OMEs by prediction class in the OPERAS testing set and the external Newcastle cohort. A sample of example cases is presented with variables, predictions, the original clinician’s prescribing decision, and the patient’s actual consumption outcome. Further, the TabPFN regression model was applied to assess the range of quantities of opioids predicted to be consumed within 1 week post-discharge. In the OPERAS testing set, where patient-reported outcomes were available, we then developed counterfactual simulations to assess the impact on quality of life (EQ-5D score), proportion of time spent in severe pain (0–100; herein pain severity), and satisfaction with analgesia (0–10). First, the observed effect was calculated as the mean difference in EQ-5D score, pain severity, and satisfaction with analgesia between those originally discharged with an opioid and those not discharged with an opioid. The counterfactual effect was then defined as the mean difference in satisfaction between (a) the group that the model predicted requiring an opioid, but clinicians did not; and (b) the group where the model predicted not requiring an opioid, but clinicians did (i.e. when model predictions disagreed with clinician decisions). The impact of the model was determined by subtracting the effect size in the counterfactual model from the observed effect. We then assessed for statistical significance by bootstrapping 95% CIs with 1000 resamples.

The global impact of applying our predictions was modelled using country-level case volume data from Holmer et al.^35^. Opioid prescribing rate was taken for available countries from the OPERAS study, and supplemented by rates described by Kaafarani et al.^26^. First the difference in rates of observed and predicted opioid prescribing was calculated and described. Thereafter, the average quantity of OME prescribed per patient was multiplied by the average prescribing rate, and the absolute difference between observed and predicted rates and presented as mean and standard deviation (SD). These data are visualised with a choropleth.

## Supplementary information

Supplementary Information

Appendix

## Data Availability

Full data are not available to be shared due to data protection issues, however, the corresponding author is can be contacted for enquiries.

## Code availability

Analysis code is available at https://github.com/kriz98/operas.
