# Author Correction: Predicting opioid consumption after surgical discharge: a multinational derivation and validation study using a foundation model

**DOI:** 10.1038/s41746-026-02749-5

**Published:** 2026-05-13

**Authors:** Chris Varghese, Luke Peters, Lorane Gaborit, William Xu, Kaviya Kalyanasundaram, Aya Basam, Melissa Park, Cameron Wells, Kenneth A. McLean, Gabriel Schamberg, Greg O’Grady, Deborah Wright, Jennifer Martin, Ewen Harrison, Peter Pockney, TASMAN Collaborative, Chris Varghese, Luke Peters, Lorane Gaborit, Kaviya Kalyanasundaram, Aya Basam, Deborah Wright, Jennifer Martin, Melissa Park, Peter Pockney, William Xu, Cameron Wells, Gabriel Schamberg, Greg O’Grady, Kenneth A. McLean, Ewen Harrison, Billy Wu, Sarah Goh, Jiting Li, Jainil Shah, Abdullah Waraich, Upasana Pathak, Amie Hilder, Muhammed Elhadi, Aiden Jabur, Christina Ohis, Chui Foong Ong, Venesa Siribaddana, Kyle Raubenheimer, Jennifer Vu, Gordon Liu, Liam Ferguson, Kristy Atherton, Amanda Dawson, Arnab Banerjee, Nagendra Dudi-Venkata, Nicholas Lightfoot, Isabella Ludbrook, Luke Peters, Rachel Sara, David Watson, Ademola Adeyeye, Luis Adrian Alvarez-Lozada, Semra Demirli Atici, Milos Buhavac, Giacomo Calini, Orestis Ioannidis, Mustafa Deniz Tepe, Upanmanyu Nath, Ahmad Uzair, Wah Yang, Faseeh Zaidi, Surya Singh, Bahiyah Abdullah, Diana Sofia Garces Palacios, Ahmed Ragab, Ahmed Ahmed, Kyle Raubenheimer, Davina Daudu, Simran Vinod Benyani, Nandini Karthikeyan, Laure Taher Mansour, Warren Seow, Zoya Tasi, Dhia Errahmane Abdelmelek, Ikram Fatima Zohra Boussahel, Oumelaz Kaabache, Naoual Lemdaoui, Oualid Nebbar, Mounira Rais, Meriem Abdoun, Aya Tinhinane Kouicem, Souad Bouaoud, Kamel Bouchenak, Hind Saada, Amel Ouyahia, Wassila Messai, Zhi Shyuan Choong, Clarissa Ting, Michelle Larkin, Pei Jun Fong, Isabel Soh, Alyssia De Grandi, Hareem Iftikhar, Akansha Sinha, Dhruv Kapoor, Tara Chlebicka, David Singer, Kim Goddard, Lisa Matthews, Rosalina Lin, Jessica Chambers, Juliet Chan, Brooke Macnab, John Barker, Morgan Mckenzie, Neil Ferguson, Ghanisht Juwaheer, Vijayaragavan Muralidharan, Sonia Gill, Nakjun Sung, Rohan Patel, Chris Walters, Kevin Nguyen, David Liu, Carlos Cabalag, Jennifer Lee, San-Hui Anita Leow, Suat Li Ng, Hamza Ashraf, Fraizer Mulder, Jonathan Loo, David Proud, Samantha Wong, Yida Zhou, Qi Rui Soh, David Chye, Sean Stevens, Patrick Tang, Stephen Kritharides, Jason Dong, Oscar Morice, Dora Huang, Andrew Hardidge, Mishka Amarasekara, Aleah Kink, Damien Bolton, Alisha Rawal, Jasraaj Singh, Matthew Heard, Yusuf Hassan, Ahmed Naqeeb, Andrew Cobden, Duron Prinsloo, Dwain Quadros, Emma Gunn, Ha Jin Kim, Jennifer Ekwebelam, James Shanahan, Mustafa Alkazali, Mariyah Hoosenally, Naveen Nara, Peter Nguyen, Sally Barker, Zacchary Tamsett, Naomi Rigby, Hinal Patel, Eloise Ferguson, Lauren Byrnes, Alexander Bravo, Amie Hilder, Ally Hui, Antara Karmakar, Bill Wang, Janindu Goonawardena, King Tung Cheung, Nicholas Chan, Ragul Natarajan, Richard Cade, Rong Jin, Shomik Sengupta, Ruth Snider, Harsha Morisetty, Lewis Weeda, Phoebe Sun, Lalitya Chilaka, Jacinta Cover, Aashrinee De Silva Abeweera Gunasekara, Rahavi Senthilrajan, Anas Alwahaib, Alexandra Limmer, Bushra Zamanbandhon, Kumail Jaffry, Yijia Shen, Alan Chua, Saifulla Syed, Sushanth Saha, John Glynatsis, Lori Aitchison, Bernard Lagana, Mason Crossman, David Watson, Abby Dawson, Bryan Fong, Ella Harrison, Eleanor Horsburgh, Michael Khoo, Kritika Mishra, Lewis Hewton, Alex Mesecke, Hien Tu, Than Tun, Jason Wong, Elynn Ong, Tara-Nyssa Law, Ashlee Landy, Alyssa Leano, Andrea Li, Akshay Soni, Benjamin Dowdle, Charles Pilgrim, Dewmi Abeysirigunawardana, Deepak Rajan Jeyarajan, Diya Patel, Jason Chung, Kyle Mckinnon, Madeline Gould, Paul Gilmore, Ruxi Geng, Rachael Loughnan, Sarahjane Norton-Smith, Solomon Nyame, Sarah Tan, Sewni Samarawickrama, Si Woo Yoon, Yantong Wang, Yichi Zhang, Zixuan Wang, Hans Mare, Indrajith Withanage, Mitali Khattar, Alexandra Toft, Goutham Sivasuthan, Hailin Zhao, Jordan Addley, Lucinda O’brien, Muhammad Raza, Randipsingh Bindra, Sonakshi Sharma, Charlotte Cornwell, Aditya Patil, Aiden Cheung, Ashleigh Lown, Amanda Dawson, Aneel Blassey, Benjamin Ochigbo, Felicity Cheng, Aleeza Fatima, Edward Zhang, Henry Kocatekin, Charles Roth, Dani Brewster, Kelvin Kwok, Paul Chen, Sharon Laura, Dominic Tynan, Edward Latif, Elizabeth Lun, Elodie Honore, Felix Ziergiebel, Jessica Blake, Karan Chandiok, Katie Bird, Lynette Ngothanh, Melissa Lee, Mariam El-Masry, Peter Hamer, Ramanathan Rm Palaniappan, Richard Mcgee, Sarah Huang, Shane Zhang, Shubhang Hariharan, Yannick De Silva, Celeste Lee, Penelope Fotheringham, Ian Incoll, Timothy Cordingley, Matthew Brown, Leannedra Kang, Rivindu Wijayaratne, Parisse Moore, Gemma Qian, Yara Elgindy, Emma Carnuccio, Hamish Rae, Mena Shehata, Mingchun Liu, Brodee Lockwood, John Van Bockxmeer, Ali Alsoudani, Daniel Swan, Justin Hsieh, Francesca Orchard-Hall, Kai Yun Jodene Tay, Raagini Mehra, Alpha Gebeh, Ashley Bailey, Georgia Brown, Ashley Colaco, Hemashree Gopal, Jessica Boyley, Varun Changati, Joseph Fletcher, Tanishq Khandelwal, Colin House, Chris O’neil, Emily Jaarsma, Victor Ly, Zsolt Balogh, Amanda Shui, Vinogi Sathasivam, Hannah Legge-Wilkinson, King Ho Wong, Andrew Chen, Anthony Tran, Peter Rehfisch, Grace Wang, Jonathan Nguyen, Joshua Peker, Kayla Gallert, Mia Komesaroff, Manideep Namburi, Elisabeth Goldfinch, Ropafadzo Muchabaiwa, Aishwarya Jangam, Isobel Taylor, Iulian Nusem, Jin Hyuk (David) Park, Justin Gundara, Rachael Heigan, Tam Tran, Thomas Mackay, Yasmine Butterworth, Tomas Sadauskas, Melody Tung, Hasthika Ellepola, Christine Gan, Hakim Fong, Ankita Das, Leshya Naicker, Samantha Hauptman, Aditi Kamath, Anthea Yew, Anupam Parange, Katie Kim, Sahil Kharwadkar, Tharushi Gamage, Lucille Vance, Alexandra Seldon, Moheb Ghaly, Jainam Shah, Victoria Phan, Karanjeet Chauhan, Ahmad Bassam, Beverley Vollenhoven, Kumail Jaffry, Kajal Mandhan, Mithra Sritharan, Mahesh Sakthivel, Natalie Evans, Samuel Robinson, Seiyon Sivakumar, Liberty Marrison, David Jollow, Krishma Joshi, Steve Tao, Pallavi Shrestha, Sai Keerthana Nukala, Russell Hodgson, Anna Crotty, Adriana Esho, Alasdair Harris, Amy Surkitt, Laura Bland, Blake Mcleod, Chonghao Yin, Cambo Keng, Emily Greenwood, Grace Yuan, Emma Haege, Hongyi Wu, Haotian Xiao, Isabella Pozzi, Jeff Fu, Jessica Stott Ross, Juliette Gentle, Kathy Gan, Kelvin Chang, Kexin Sun, Madhavi Singh, Maria Xie, Nicholas Mccabe, Mark Slavec, Nick Clarnette, Behzad Niknami, Peishan Zou, Sean Flintoft, Shenuka Jayatilleke, Rumnea Sok, Suqi Tan, Sanya Wadhwa, Will Swansson, Daniel Abulafia, Jian Blundell, Amie Sweetapple, Caitlin Del Solar, Cameron Martin, David Bell, Isuru Fernando, Jared Chang, Katie Vanzuylekom, Katie Van Zuylekom, Kate Van Zuylekom, Katie Hobbs, Richard Liang, Aiden Jabur, Jazmina Tarmidi, Mahmoud Ugool, Nicholas Beatson, Sarah Bowman, Sophie Moin, Wen Po Jonathan Tan, Seevakan Chidambaram, Siang Wei Gan, Pengnan Wang, Leshya Naicker, Katie Kim, Nicole Qiwen Wang, Yi Xin Kwan, Chinmai Patil, Divyanshu Joshi, Aditi Kamath, Aishath Hanan, Arfaan Sheriff, Jaime Duffield, Leshya Naiker, Peter Smitham, Eu Ling Neo, Matthew Chua, Shalvin Prasad, Armitesh Nagaratnam, Tarik Sammour, Yuxin Lin, Christine Lee, Eve Hopping, Muskan Jangra, Ankita Das, Ken Lin, Zachary Bunjo, Kyle Raubenheimer, Mohamed Haseef Mohamed Yunos, Kar Long Yeung, Rachel Phu, Aisling Betts, Benjamin Just, Sahil Gera, Hilary Leeson, Jodie Jamieson, Katie Wang, Emily Luu, Michael Innes, Jennifer Vu, Jonathan Hong, Stephen Dzator, Aki Flame, Vincent Jiang, Jianing Kwok, Aaron Lawrence, Kate Meads, Liam Pearce, Pavatharane Sarangadasa, Haylee Shaw, Victor Yu, Elizabeth Crostella, James Wong, Sriya Bobba, Maddison Muller, Yin Chi Hebe Hau, Thomas Wilson, Aleksandra Markovic, Jemma Green, Clara Forbes, Emalee Burrows, Lachlan Hou, Clare O’sullivan, Jonathon Foo, Hannah Greig, A. -J Collins, Callum Chandler, Emily Heaney, Hannah Gross, Monica Morgan, Rebecca Loder, Krishnankutty Rajesh, Shravankrishna Ananthapadmanabhan, Akeedh Razmi, Crystal Vong, Prasanna Pothukuchi, Mary Theophilus, Roshni Sriranjan, Sharon Kaur, Marcelo Kanczuk, Julia De Groot, Angela Corrigan, Damon Li, Danniel Badri, Dominico Ciranni, Elangovan Thaya Needi, Matthew Clanfield, Nicolas Copertino, William Rumble, Maria Kristina Vanguardia, Chen Lew, Rami Dennaoui, Jainil Shah, Joseph Kong, Imogen Koh, Raymond Zeng, Kristian Baziotis-Kalfas, Hannah Denby, Andy Li, Will Tran, Abhinav Singh, Olivia Lin, Michelle Chau, Olivia Donaldson, Christina (Seojung) Min, Shirahn Ballah, Sonia Ching Ting Tsui, Nathania Yong, Lucy Standish, Sarah Tan, Asuka Fujihara, Lily Davies, Ramin Odisho, Anjana Ravi, Josh Collins, Pooja Chandra, Rana Abdelmeguid, Gopal Singh, Xireaili Feierdaiweisi, Dharani Seneviratne, Shambhavi Srivastava, Michelle Yao, Cherilyn Teng, Nebula Chowdhury, Sasini Vidanagama, Charles Lin, Tharushi Sampatha-Waduge, Erica Wang, Chatnapa Yodkitydomying, Julia Silverii, Aaron Lam, Krisha Solanki, Angus Franks, Liam Edwards, Ridvan Atilhan, Rohan Nandurkar, Oliver Wells, Kristina Vanguardia, Dennis King, Elton Edwards, Quang Tran, Seojung Min, Abdul Rauf, Yangzirui Fu, Hodo Haximolla, Mengge Shang, Sharrada Segaran, Shelley Wang, Gananadha Sivakumar, Jaspreet Kaur Sandhu, Neel Mishra, Samantha Hauptman, Alyssa Chua, Danielle Chene, Guy Maddern, Henry Shaw, Qiwen Wang, Siyuan Pang, Christine Lu, James Fung, Kathryn Cyr, Karen Lu, Ming Zhou How, Nelson Hu, Paul Anderson, Philip Jakanovski, Arkan Youssef, Howard Tang, Rory Keenan, Alex Chan, Mitch Canny, Farah Tahir, James Egerton, Justin Yeung, Justin Chan, Lea Tiffany, Michael Bei, Mariolyn Raj, Peter Williams, Sakshar Nagpal, Tim Outhred, Russel Krawitz, Colin Chan-Min Choi, Khadijah Younus, Mary Giurgius, Rosemary Kirk, Amanda Gonzalez Pegorer, Pattarapan Tang-Ieam, Jack Ward, Asanka Wijetunga, Caitlin Zhang, Chris Nahm, Christine Wang, Damian Golja, Gregory Jenkins, Helena Qian, Jason Luong, Kim Nguyen, Sean Suttor, Sherman Lai, Vanessa Ma, Yan Chen, Hoi Hang Yu, Amos Lee, Antonio Barbaro, Cameron Mcguinness, Guy Maddern, Stevie Young, Ye Fang Lim, Georgina Trotta, Phoebe Chao, George Ding, Carol Fang, Andi Lu, Prabhath Wagaarachchi, Charlotte Cornwell, Amy Gojnich, Peter Stewart, Isabella Dong, Kenneth Wong, Luca Burruso, Lucinda Hogan, Nathan Mcorist, Ramnik Singh, Ragavi Jeyamohan, Zhen Hou, William Lai, Emily Taylor, Maria Alejandra Nanez Pantoja, Daniel Mauricio Bolanos Nanez, Gilmer Omar Perez Hernandez, Lia Jasmin Jimenez Ramirez, Mohamed Mohamed, Ahmed Kamal El-Taher, Ahmed Elewa, Mahmoud Ayman Soliman, Menna Diab, Radwa Ali, Ahmed Ahmed, Adham Galal, Ahmed Elkhodary, Ali Alaa, Arwa Faisal, Asmaa Badawy, Donia Eldomiaty, Mohamed Al Sayed, Esraa Rasslan, Mohamed Ramadan, Gamal Elsayed Fares, Hashem Altabbaa, Humam Emad, Muneera Alboridy, Mahmoud Mongy, Osama Albarhomy, Osama Selim, Rawan Rafaei, Raneem Atta, Ahmad Altaweel, Yara Sherif, Youssef Elghoul, Yousef Tarek, Ahmed Abdelfatah Sabry, Ahmad Moustafa, Osama AbouHiekal, Osama Al Shaqran, Zeyad Haggag, Dina Atef, Ahmed Mahmoud, Mahmoud Saad, Mohamed Ragab, Aya Hussien, Mostafa Abdelbaky, Ismail Muhammad, Afnan Morad, Ahmed Ali, Ahmed Hussien, Ahmed Shipa, Ahmed Aboulfotouh, Ahmed Mohamed Hashem, Ahmed Morsi, Alshymaa Ebrahim, Ahmed Mohamed Sayed, Amira Abdelrahman, Aml Ali, Samah Abdelnaeam, Asmaa Emam, Aya Shaban, Fady Barsoum, Esraa Mostafa, Doaa Abdelbaset, Dina Othman, Safaa Othman, Nour Salah Khairallah, Salma Morsi, Armia Azer, Enas Abdelbaset Abdelsamed, Islam Ibrahim, Esraa Abdelbaset, Esraa Hamoda, Fatma Monib, Fatma Harb, Hager Maher, Haitham Mohammed, Kerollos Henes, Kerollos Shamshoon, Mahmoud Hassanein, Magdy Mahdy, Mahmoud Khalil, Manal Ali, Mansour Khalifa, Marwa Amary, Merna Ezz Suliman, Mohammed Saif Al Nasr, Michael Elia, Michael Adly, men Roshdy, Mohammed Al-Quossi, Mohammed Fargaly, Mona Saber, Mostafa Abbas, Ola Haroon, Omima Khalil, Omnia Talaat, Rahma Elnagar, Randa Soliman, Reham Aboelela, Salem Salah, Samia Abdelgawad, Tarek Hussien, George Sobhy, Yasmeen Sayed, Yousra Othman, Reham Silem, Ali Dawood, Tarek Hemaida, Reem Ahmed, Aya Kamal, Mohamed Salah, Ahmed Zaharia, Ebrahim Salem, Osama Fathy Ali Ali Rashed, Mohamed Halawa, Hossam Elfeki, Abdelrahman Mosaad, Abdelrahman Shaaban, Hebatalla Abdelsalam, Ahmed Sakr, Aly Sanad, Amr Elsawy, Bassant Maged Maged, Dana Hegazy, Mohamed Abdelmaksoud, Mahmoud Laymon, Mohamed Taman, Esraa R. Moawad, Hadeer Elsaeed Aboelfarh, Karim Elkenawi, Manar Osama, Mirna Sadek, Mohamed Abdelaziz Elghazy, Mohammed Attiah, Mohamed Nader, Mostafa Shalaby, Omar Attiya, Osama Samir Gaarour, Ahmed Zaghloul, Pola Mikhail, Karim Badr, Hatem Soltan, Mohamed Donia, Mohammed Gaafar, Khaled Abdelwahab, Abdelaziz Sallam, Ahmed Eid, Mohamed Yousri, Omar Hamdy, Aiman Al-Touny, Abdelrhman Alshawadfy, Ahmed Hamdy, Ahmed Ellilly, Ahmed Mahdy, Ahmed El-Sakka, Hamdy Hendawy, Asmaa Salah, Bassma Raslan, Eman Teema, Eslam Albayadi, Esraa Nasser, Hanaa Mohamed, Mohamed Mahmoud, Mostafa Elsaied, Omima Taha, Shaimaa Dahshan, Shimaa Al-Touny, Ahmed Karrar, Ahmed Khairy, Abdelrahman Farag, Asmaa Deafallah, Alaa Mohamed Ads, Rabiaa Alomar, Issa AbuShawareb, Abdallah Saeed, Abdelhafeez Mashaal, Adel Mohamed Ads, Sohila Ghanem, Ahmed Elghamry, Eman Ayman Nada, Youssef Ali Noureldin, Mohamed Fayez Fouda, Nourhan Shaheen, Shereen Allam, Ibrahim Mazrou, Ali Fahmy Shehab, Wesam Kussaili, Dimitrios Korkolis, Evangelos Fradelos, Aikaterini Sarafi, Nikolaos Machairas, Konstantinos S. Giannakopoulos, Fotios Stavratis, Georgios Korovesis, Gerasimos Tsourouflis, Myrto D. Keramida, Nikolaos Kydonakis, Stylianos Kykalos, Athanasios Syllaios, Panagiotis Dorovinis, Dimitrios Schizas, Orestis Ioannidis, Anastasia Malliora, Elissavet Anestiadou, Konstantinos Zapsalis, Fotios Kontidis, Lydia Loutzidou, Nikolaos Ouzounidis, Stefanos Bitsianis, Savvas Symeonidis, Smaragda Skalidou, Olga Maria Valaroutsou, Themistoklis Dagklis, Alexandra Arvanitaki, Apostolos Mamopoulos, Apostolos Athanasiadis, Stergios Kopatsaris, Ioannis Kalogiannidis, Ioannis Tsakiridis, Georgios Kapetanios, Evangelos Papanikolaou, Nikolaos Tsakiridis, Fotios Zachomitros, Andreas Larentzakis, Argyrios Gyftopoulos, Konstantinos Albanopoulos, Apostolos Champipis, Christos Yiannakopoulos, Gavriella Zoi Vrakopoulou, Konstantinos Saliaris, Konstantinos Lathouras, Spyridon Skoufias, Georgia Doulami, Metaxia Bareka, Eleni Arnaoutoglou, Fragkiskos Angelis, Fragkiskos Angeslis, Michael Hantes, Maria Ntalouka, Maytham A. Al-Juaifari, Mohammed Alwash, Rasool Maala, Yasir Adnan Zwain, Sara Ahmed Saleh, Mohammed Khorsheed, Antonio Pesce, Carlo V. Feo, Massimiliano Bernabei, Francesca Petrarulo, Nicolò Fabbri, Raffaele Labriola, Silvia Jasmine Barbara, Simone Bosi, Angela Romano, Anna Canavese, Caterina Catalioto, Claudio Isopi, Cristina Larotonda, Gerti Dajti, Matteo Rottoli, Iris Shari Russo, Stefano Cardelli, Francesco Castagnini, Francesco Traina, Giulia Guizzardi, Giulia Giuzzardi, Mara Gorgone, Marco Maestri, Pasquale Cianci, Ivana Conversano, Enrico Restini, Domenico Gattulli, Giorgia Grillea, Marco Varesano, Giacomo Calini, Adelaide Andriani, Davide Gattesco, Giovanni Terrosu, Mattia Zambon, Pietro Matucci Cerinic, Luisa Moretti, Davide Muschitiello, Samantha Polo, Vittorio Bresadola, Salah Abu Wardeh, Mahmoud Al-Baw, Saif Alhaleeq, Subhi Al-Issawi, Abdalqader Al Smadi, Esmat Alsaify, Farah Banihani, Noor Massadeh, Nada Massadeh, Dima Al-issawi, Basel Elyan, Qotadah Al-Shami, Yazan Alomari, Abed Alazeez Alkhatib, Bader Alzghoul, Ahmad Saleh, Jamal Yaghmour, Mahmoud Shahin, Mohammed Maali, Dawood Alatefi, Heba Al-Smirat, Abdulhakim Hezam, Nassar Alathameen, Amr Al Hammoud, Abdulrahim Al Kaddah, Salem Ayasrah, Hamza Abuuqteish, Tesneem Al-Mwajeh, Reena Makableh, Saad Bataineh, Amin Shabaneh, Wesam Alnatsheh, Marwan Aldeges, Huda Hamad, Sireen Shehahda, Dima Khassawneh, Osama Alzyoud, Risan Alrosan, Hasan Awad, Tariq Khaldoon, Rabab Shannaq, Mohammad Al hamoud, Bader Abo fadalah, ath Al-Hazaimeh, Wail Khraise, Lara Alnajjar, Majjd Alnajjar, Sohaib Al-Omary, Adnan Ababneh, Alaa Albashaireh, Mohammad Khadrawi, Mohammad Aljamal, Tayseer Athamneh, Ro-a Muqbel, Maryam Al-jammal, Ahmad Masarrat, Alia Al-zawaydeh, Ibrahim Taha, Taima’ Qattawi, Rayyan Smadi, Ayah Alhaleem, Mosab Alboon, Omar Hazaymeh, Leen Karasneh, Safa’ Al-Haek, Marin Almahroush, Tamam Alfrijat, Aya Elporgay, Hadeel Shanag, Hamza Agilla, Hind Alameen, Marya Bensalem, Mawadda Altair, Malak Ghemmied, Rehab Alarabi, Sara Alhudhairy, Rima Gweder, Amal Alzarroug, Ebtihal Alabed, Fadwa Elreaid, Omar A. Elkharaz, Fatma Fathi Elreaid, Safa Sasi Albatni, Haitham Elmehdawi, Milad Gahwagi, Ayman Mohamed, Tariq Alfrjani, Khaled Khafifi, Ayat Rasheed, Ayoub Akwaisah, Hassan Bushaala, Mustafa Elfadli, Mohamed Moftah, Salima Algabbasi, Salma Esaiti, Sara Elfallah, Abtisam Alharam, Fatima Alariby, Mohamed Isweesi, Tarik Ahmed Eldarat, Ayman Arhuma Dabas, Akram Alkaseek, Ahmed Mohammed Abodina, Aya Alqaarh, Hibah Bileid Bakeer, Hoda Salem Alhaddad, Husein Aboudlal, Sawsan Alsaih, Noora Abubaker, Najwa Abdelrahim, Ali Alzarga, Basma Omar, Farah Faris, Qamrah Alhadad, Asma Abufanas, Hussameddin Badi, Israa Benismai, Hawa Obeid, Abdulwahab Abdalei, Ahmed Abdulrahman, Aisha Swalem, Ebtisam Alzarouq, Amna Safar, Esra Shagroun, Boshra Hashem, Fatheia Elrishi, Fatima Abdulali, Habeeba Ahmed, Ibrahim Eltaib, Joma Elzoubia, Aisha Albarki, Hoda El Mugassabi, Fatima Abushaala, Amany Abuzaho, Nida Juha, Raneem Egzait, Sundes Shetwan, Alzahra Lemhaishi, Faisel Matoug, Eman Abdulwahed, Aamal Askar, Abir Ben Ashur, Adel Bezweek, Bushra Altughar, David Emhimmed, Donia Elferis, Laila Elgherwi, Enas Soula, Doaa Gidiem, Maren Grada, Khawla Derwish, Maram Alameen, Nassib Algatanesh, Ahlam Elkheshebi, Reem Ghmagh, Sharf Barka, Sultan Ahmeed, Sarah Aljamal, Zahra Alragig, Mohamed Addalla, Ahmed Atia, Atab Kharim, Fathia Mahmoud, Muhannud Binnawara, Entisar Alshareea, Mohamed Alsori, Aisha Alshawesh, Ghaliya Mohamed H. Alrifae, Amira Ashour, Anwaar Abozid, Asil Omar Saleh Alflite, Anwar Mohamed, Jaber Arebi, Fatma Alagelli, Hana Yousef Gineeb, Rawia Ghmagh, Rihab Mohammed Bin Omar, Retaj Alaqoubi, Sara Mohammed, Serien Hossain Bensalem, Tahani Elgadi, Wesam Sami, Yara Bariun, Abdulhadi Mohammed Alhadi Alhashimi, Dheba Almukhtar Abdulla, Heba Rhuma, Husam Enaami, Asraa Ali Alboueishi, Hayat Ben Hasan, Mohamd A. A. Alkchr, Bashir Albakosh, Najah Alsari, Mahammed Aldreawi, Najat Ben Hasan, Khaled Abushanab, Rawad Yahya, Narimantas Samalavicius, Vitalijus Eismontas, Jonas Jurgaitis, Oleg Aliosin, Vitalija Nutautiene, Andee Dzulkarnaen Zakaria, Anil Kumar Sree Kumar Pillai, Dinesh Kumar Vadioaloo, Mohamed Ashraf Mohamed Daud, Jien Yen Soh, Mohd Zaim Zakaria, Siti Mayuha Rusli, Nur Ayuni Khirul Ashar, Zatul Akmar Ahmad, Afiq Aizat Ramlee, Sharifah Nor Amirah Syed Abdul Latiff Alsagoff, Ahmad Anuar Sofian, Muhammad Badrul Hisyam Mohamad Jamil, Bahiyah Abdullah, Mohamad Faiz Noorman, Muhammad Fihmi Zainal Abidin, Mohamed Izzad Isahak, Siti Nasyirah Nisya Adnan, Zaidatul Husna Mohamad Noor, Luis Adrian Alvarez-Lozada, Alejandro Quiroga Garza, Andrea Aguilar Leal, Bernardo Alfonso Fernández Reyes, Ethel Valeria Orta Guerra, Francisco Javier Arrambide Garza, Héctor Erasmo Alcocer Mey, Jorge Arath Rosales Isais, Juventino Tadeo Guerrero Zertuche, Patricia Ludivina González García, Luis Antonio Heredia Sánchez, Marcela Patricia Flores Mercado, Oscar Alonso Verduzco Sierra, Pedro Emiliano Ramos Morales, Stephie Oyervides Fuentes, Víctor Manuel Peña Martínez, Yesika Alejandra Guerra-Juárez, Ana Karina Flores-González, Surya Singh, Arwa Hadi, Christian Woodbridge, David Thornton-Hume, Jack Forsythe, Isini Dharmaratne, Vivian Pai, John Windsor, Kamran Zargar, Lucy Waldin, Lily Winthrop, Matias Alvarez, Meileen Huang, Matt Kumove, Marta Simonetti, Namisha Chand, Oliver Goldsmith, Oscar Guo, Paul Monk, Karen Zhou, Sai Harshitha Penneru, Shaamnil Prasad, Seifei Ren, Terrence Hill, Vyoma Mistry, Selena Sun, Ashley Pereira, Scott Mclaughlin, Andrew Stokes, Avinash Sathiyaseelan, Jeremy Rossaak, Janice Lim, Kenya Brooke, Liam Quinlan, Mark Pottier, Nayanika Podder, Puja Jinu, Shanay Ramphal, Wikus Vermeulen, Flavio Ordones, Fraser Jeffery, Ibrahim S. Al Busaidi, Janelle Divinagracia, William Ju, Yizhuo Liu, Tamara Glyn, Nasya Thompson, Vivien Graziadei, Joshua Canton, Joseph Furey, Horim Choi, Grace Coomber, Tanya Divekar, Tessa English, Erin Gernhoefer, Tom Healy, Justin Chou, Dikshya Parajuli, Catherine Reed, Rod Studd, Anthony Lin, Cameron Wells, Cindy Xu, Arwa Hadi, Andrew Maccormick, Heejun Park, Athulya Rathnayake, Brittany Williams, Ashley Chan, Corinne Smith, Francesca Casciola, Jainey Bhikha, Jonathan Luo, Kevin Yi, Megan Singhal, Ria George, Rosie Luo, Taylor Frost, Fatima Hakak, Akhita George, Angela Carlos, Annie Ho, Connor Mcrae, Jonathan Lescheid, Jenny Soek, Andrew Pham, Sophie St Clair, Su-Ann Yee, Jennifer Lim, Chun-Yen Wu, Taehoon Kim, Anne Qi Chua, Christopher Harmston, Hamish Boyes, Holly Cook, Jamie Struthers, Jess Radovanovich, Nicholas Quek, Chekodi Fearnley-Fitzgerald, Deborah Wright, Kushan Ghandi, Natalie Matheson, Matthew James McGuinness, Brian Chen, Rebecca Indiana Douglas, Konrad Richter, Nisha Bianca Soliman, Scott Matthew Bolam, Vineeth Vimalan, William Currie, Mitchell Cuthbert, Poppy Ross, Amy Nicholson, Briar Garton, Emilie Agnew, Niamh Conlon, Nicholas Waaka, Ritwik Kejriwal, Sean Nguyen, Edmund Leung, Milidu Ratnayake, Quintin Smith, Nejo Joseph, Bosco Yue, Calvin Fraser, Charles Lam, Ethan Figgitt, Gordon Liu, Kevin Tan, Ha Seong You, Helen Zheng, Jenny Luo, James Sharp, Kabir Khanna, Levi Simiona, Michel Luo, Patrick Wong, Rebecca Luu, Rohit Paul, Shiva Nair, Shadie Asadyari-Lupo, Wing Hung, Geoffrey Ying, Jess Ho, Alan Wu, Eamon Walsh, Jouyee Lee, Jessie Liu, Sunny Yao, Omar Nosseir, Jennifer Dang, Simon Young, Sof’ya Zyul’korneeva, Theresa Boyd, Jess Ho, Alan Wu, Sunny Yao, Abdullahi Musa Kirfi, Adamu Bala Ningi, Mohammad Albuhari Garba, Makama Baje Salihu, Ohia Ernest Ukwuoma, Abdullahi Ibrahim, Isa Mienda Sajo, Muhammad Baffah Aminu, Liman Haruna Usman, Oloko Nasirudeen Lanre, Ibrahim Shaphat Shuaibu, Stephen Yusuf, Tiamiyu Ismail, Gabi Ibrahim Umar, Ademola Adeyeye, Ehis Afeikhena, Favour Chinenye Nnaji, Joy Onyekachi Agu, Temiloluwa Peace Maxwell, Oluwatosin Olakunle Motajo, Oghenekaro Ifoto, Seubong-Abasi Imoh Okon, Jerry Godfrey Makama, Amina Abosede Mohammed-Durosinlorun, Bashiru Aminu, Polite Iwedike Onwuhafua, Caleb Mohammed, Lubabatu Abdulrasheed, Joel Amwe Adze, Khadijah Richifa Suleiman, Lydia Regina Airede, Mathew Chum Taingson, Stephen Bodam Bature, Stephen Akau Kache, Uchechukwu Ohijie Ogbonna, Mohammed Bello Fufore, Abdulkarim Iya, Adeshina A. Ajulo, Ahmad Mahmud, Bilal Shuaibu Yahya, Farida Onimisi-Yusuf, Hope Isaac, Timothy Jawa, Fashe Joseph, Bemi Kala, Maisaratu A. Bakari, David Wujika Ngwan, Abubakar umar, Abraham L. Filikus, Daniel Wycliff, Abiodun Okunlola, Olukayode Abiola, Adebayo Adeniyi, Olabisi Adeyemo, Babatunde Awoyinka, Olakunle Babalola, Adewumi Bakare, Taiwo Buari, Cecilia Okunlola, Gbadebo Adeleye, Adedayo Salawu, Henry Abiyere, Adetolu Ogidi, Tesleem Orewole, Habiba Ibrahim Abdullahi, Godwin Akaba, Arome Achem, Asi-oqua Bassey, Emeka Ayogu, Bilal Sulaiman, Dennis Anthony Isah, Chukwunonso Nnamdi Akpamgbo, Felicia Asudo, Nathaniel Adewole, Omachoko Oguche, Peter Ejembi, Samuel Ali Sani, Paul Chimezie Andrew, AliyuYabagi Isah, Bolarinwa Eniola, Zumnan Songden, Teddy Agida, Terkaa Atim, Taofiq Olayinka Mohammed, Hadijat Olaide Raji, Femi Ibiyemi, Hafeez Salawu, Olushola Fasiku, Remi Sanyaolu Solagbade, Mariam Motunrayo Shiru, Gbadebo Hakeem Ibraheem, Justina Oruade, Grace Ezeoke, Tabish Chawla, Aliya Begum Aziz, Anoosha Marium, Ayesha Akbar Waheed, Faiqa Binte Aamir, Faiza Qureshi, M. Hammad Ather, Iqra Fatima Munawar Ali, Izza Tahir, Maha Ghulam Akbar, Ronika Devi Ukrani, Sajjan Raja, Sehar Salim Virani, Shahryar Noordin, Saif Ur Rehman, Shalni Golani, Syed Roohan Aamir, Syed Musa Mufarrih, Usama Waqar, Maliha Taufiq, Ahmed Siddique Ammar, Adya Ejaz, Albash Sarwar, Ahmed Usman Khalid, Shehrbano Khattak, Aliza Imran, Omer Bin Khalid, Urauba Kaleem, Urwah Muneer, Yumna Kashaf, Fatima Zafar, Adil Zaheer, Muhammad Ali, Amna Shafaat, Arisha Qazi, Asjad Imran, Mahnoor Tariq, Muhammad Nadeem Aslam, Shehroz Ali, Tabish Atiq, Tayyiba Wasim, Daniyal Babar, Ahmad Zain, Muhammad Ibtisam, Uzair Ahmed, Syed Talha Bin Aqeel, Muhammad Muhib, Muhammad Anas Abbal, Nasar Ahmad Khan, Imran Javed, Layth Alkaraja, Dana Amro, Ghaida Manasrah, Ibraheem Hammouri, Ihab Abu Hilail, Jihad Zalloum, Laith Alamlih, Mahmoud Nasereddin, Munia Rajabi, Sa’ed Shalalfeh, Zeinab Natsheh, Khamis Elessi, Mustafa Abu Jayyab, Mohammed Astal, Mosheer Al-Dahdouh, Alaa Eddin Salameh, Alaa Ayyad, Nimatee Dawod, Hamza Alsaid, Iyas Matar, Majd Hassan, Mohammed Bakeer, Mohammad Malasah, Shehab Abuhashem, Mohammed Salem, Sorinel Lunca, Mihail Gabriel Dimofte, Stefan Morarasu, Ana Maria Musina, Cristian Ene Roata, Natalia Velenciuc, Aleksandr Butyrskii, Maxim Bozhko, Amet Ametov, Sharfuddin Chowdhury, Doaa Bagazi, Julio Domenech, Alejandro Rosello-Añon, Ana Monis, Caterina Chiappe, Beatriz Cuneo, Pablo Clemente-Navarro, Jorge Febre, Jorge Sanz-Romera, Marcos Lopez-Vega, Ignacio Miranda, Rocio Valverde-Vazquez, Sara Garcia, Maria Jose Sanguesa, Zutoia Balciscueta, Enrique Ruiz, Eduardo Marco, Elena Talavera, Joan Farre, Loreto Bacariza, Mireia Duart, Violeta Ureña, Xenia Carre, Hytham K. S. Hamid, Montasir A. Abd-Albain, Sami Galal-Eldin, Monira Sarih, Eithar Adam, Samir Ismail, Malaz Azhari, Tawfieg Hassan, Mohamed Salaheldein, Zainab Abdalla, Wahiba Ahmed, Monzer Abdulatif Mohamed Alhassan, Hozifa Mohamed Abdalla Suliman, Hozifa Mohamed Bdalla Suliman, Rogia Ahmed Abdalla Ahmed, Enas Mohammedtom Abdulhameed Babekir, Munya Ali Talab Khairy, Maha Mukhtar Ahmed Mukhtar, Rzan Ali Hamedelneel Ali, Yasir Babkir Ali Al-Shambaty, Fatima Imad Yousif, Hawa Mohammed Hassan Mohammed, Lana Osher, Menhag Abdelbast, Mohamed Yassin, Noon Moawia, Rowa Abdalsadeg, Abrar Husein, Baraa Elhassan, Alnazeer Y. Abdelbagi, Mohammed A. Adam, Eithar M. Ali, Ibrahim A. B. Mohammed, Maab Mohamed, Mohamed Abdulaziz, Mazin Akasha, Muaz Hassan, Nadir Hilal, Noon Abdalla Abdelrahman Mohamed, Noora Abubaker, Omeralfarouk Mohammed, Shakir Mohamed, Walaa Osman, Fatima Mustafa, Alaa A. Salih, Doua Ali, Doha Mohammed Ahmed Almakki, Hanan Elnour Mohamed, Abdelhadi Elmubark, Mohamed Hassan, Ammar Alnour, Amna Elaagib, Ayman Abdelrahman, Mubarak Abdelkhalig, Khalid Nour Eldaim, Afra Babiker, Entisar Ahmed, Maab Ali, Eman Hussain, Mansour Wedatalla, Alaaaldeen Ahmed, Alla Aldeen Hamza, Mohab Mohammed, Omer Osman, Reham Ibrahim, Rihab Ahmed, Ruaa Ahmed, Ruaa Yasir, Safaa Awadallah, Sara Mohmmed, Suhaib Hassan, Walid Shaban, Aisha Hussein, Reem Rafea, Ahmed Abdalla, Abdalla Ahmed, Khalid Mohamed, Mansour Mohammed, Mohamed Altahir, Mohammed Adam, Omer Mohamed, Walaa Abdullah, Hammad Fadlalmola, Ahmed Yassir Abdalla, Ahmed Ali Omer, Ahmed Alfatih Mustafa, Rawan Elnoman Elhadi, Essam Eldien Abuobaida Banaga, Fatima Osman, Mohamed Galal Ali Abdalla, Hala Abdelhalim Mohamed Taha, Noon Ezzeldien Abdalmahmoud, Rofuida Hussien Nafie, Sami Jamal, Sharwany Ahmed, Doha Amir AtaAlmanan, Rawan Alsheikh Ali, Abdallah Aladna, Abdullah Aljoumaa, Hamdi Nawfal, Salma Jamali, Fatima Khouja, Ammar Niazi, Al Rawashdeh, Nahla Kechiche, Mouna Gara, Mouna Nasr, Marwen Baccar, Oumayma Benamor, Sawssen Chakroun, Ahmet Necati Sanli, Ahmet Yildiz, Mehmet Ali Demirkiran, Yildiz Buyukdereli Atadag, Yusuf Iskender Tandogan, Esin Ozkan, Yıldırım Ozer, Muhammed Miran Oncel, Senad Kalkan, Tolga Gover, Berke Manoglu, Ilayda Oksak, Ipek Kurt, Kerem Rifaioglu, Selman Sokmen, Tayfun Bisgin, Yasemin Yildirim, Abdil Yetkin Keskin, Tugce Dogan, Berfin İlgaz Sahin, Cemil Aydin, Duygu Ece Benek, Hale Nur Tiras, Mert Arslangilay, Mert Aslangilay, Muhammet Yaytokgil, Mehmet Ali Capar, Yasemin Yazgan, Sebnem Bektas, Ahmet Can Alagoz, Alara Ece Dagsali, Aylin Izgis, Kadir Uzel, Mustafa Soytas, Niyazi Cakir, Abdullah Emre Askin, Ibrahim Azboy, Kubilay Sabuncu, Merve Aslan, Melek Sahin, Mustafa Oncel, Nuri Okkabaz, Ramazan Kemal Sivrikaya, Alparslan Saylar, Dr. Alparslan Saylar, Meltem Yasar, Ergin Erginoz, Haktan Ovul Bozkir, Kagan Zengin, Mehmet Faik Ozcelik, Server Sezgin Uludag, Zeynep Ozdemir, Osman Sibic, Hatice Telci, Mehmet Abdussamet Bozkurt, Yasin Kara, Mustafa Deniz Tepe, Adnan Gündoğdu, Bilge Akın, Dilan Pehlivan, Ali Guner, Duygu Baysallar, Berkay Yıldız, Hale Cepe, Murat Emre Reis, Ayse Nilufer Yuzgec, Nurtac Kıralı, Taha Anıl Kodalak, Mehmet Ulusahin, Kamar Selim, Ahmet Kale, Mehmet Emre Gecici, Melis Ozbilen, Zeynep Düzyol, Aylin Gemici, Elzem Korkmaz, Eminenur Şen, Muhammed Enes Taşcı, Elifsu Camkıran, Güşta Elieyioğlu, İkbal Kayabaş, Tevfik Kıvılcım Uprak, Canan Aral, Ayten Saraçoğlu, Mustafa Ümit Uğurlu, Zeynep Hazal Baltacı, Ege Nur Akkaya, Cem Fergar, Elif Zeynep Tabak, Guldane Zehra Kocyigit, Ilgaz Kayilioglu, Süleyman Polat, Elif Çolak, Mehmet Emin Kara, Mert Candan, Mustafa Safa Uyanık, Ahmet Can Sarı, Attila Ulkucu, Alperen Taha Certel, Arzu Dindar, Beyza Durdu, Cigdem Bayram, Eslem Kaya, Hakan Akdere, Ibrahim Ethem Cakcak, Ikranur Yavuz, Mert Omur, Mirac Ajredini, Erhan Onur Aydoğdu, Eylül Şenödeyici, Ulku Ceren Koksoy, Baturay Kansu Kazbek, Deniz Serim Korkmaz, Dogancan Yavuz, Hakan Yilmaz, Zeynep Sahan Cetınkaya, Elif Durmus, Filiz Tuzuner, Furkan Hokelekli, Mucahid Mutlu, Seyma Orcan Akbuz, Ziya Can Kus, Michael Farrell, Alayna Craig-Lucas, Matthew Painter, Ashley Titan, Aditya Narayan, Bunmi Fariyike, Lisa Knowlton, Tiffany Yue, Emily Benham, Abdelrahman Nimeri, Hope Werenski, Nicole Kaiser, Caroline Reinke

**Affiliations:** 1 Department of Surgery, University of Auckland, Auckland, New Zealand; 2 Department of Surgery, Mayo Clinic, Rochester, MN USA; 3 Department of Surgery, University of Newcastle, Newcastle, NSW Australia; 4 Australian National University, Canberra, ACT Australia; 5 University of Adelaide, Adelaide, SA Australia; 6 Monash University, Melbourne, VIC Australia; 7 Northern Sydney Local Health District, Sydney, NSW Australia; 8 Department of Clinical Surgery, University of Edinburgh, Edinburgh, Scotland; 9 Centre for Medical Informatics, Usher Institute, University of Edinburgh, Edinburgh, Scotland; 10 Department of Surgical Sciences, University of Otago, Dunedin, New Zealand; 11 Dokuz Eylul University Hospital, Izmir, Turkey; 12 Istanbul University Cerrahpasa—Cerrahpasa School of Medicine, Istanbul, Turkey; 13 Karadeniz Technical University, Trabzon, Turkey; 14 Faculty of Medicine, Bezmialem Vakif University, Istanbul, Turkey; 15 King Abdullah University Hospital, Irbid, Jordan; 16 Kanuni Sultan Suleyman Training and Research Hospital, Istanbul, Turkey; 17 Faculty of Medicine, Çorum Research and Training Hospital, Hitit University, Çorum, Turkey; 18 Bunbury Hospital, Bunbury, WA Australia; 19 Kartal Dr. Lutfi Kirdar Training and Research Hospital, Istanbul, Turkey; 20 Northern Beaches Hospital, Sydney, NSW Australia; 21 Samsun Training and Research Hospital, Samsun, Turkey; 22 Hospital Lorenzo Bonomo, Andria, Italy; 23 New Plymouth Hospital, New Plymouth, New Zealand; 24 Palestine Medical Complex, Ramallah, Palestine; 25 Afe Babalola University Multisystem Hospital, Ado Ekiti, Nigeria; 26 The Alfred Hospital, Melbourne, VIC Australia; 27 Barau Dikko Teaching Hospital, Kaduna, Nigeria; 28 Ballarat Base Hospital, Ballarat, VIC Australia; 29 Gosford Hospital, Gosford, NSW Australia; 30 Nilratan Sircar Medical College and Hospital, Kolkata, India; 31 Istanbul Medipol University Hospital, Istanbul, Turkey; 32 Emergency Municipal Hospital No. 6, Moscow, Russia; 33 Aleppo University Hospital, Aleppo, Syria; 34 Latrobe Regional Hospital, Melbourne, VIC Australia; 35 El Tadamon Specialised Hospital, Port Said, Egypt; 36 Wellington Hospital, Wellington, New Zealand; 37 Suez Canal University Hospital, Ismailia, Egypt; 38 Hospital Sant Joan Reus, Tarragona, Spain; 39 St John of God Midland Public and Private Hospital, Perth, WA Australia; 40 New South Wales Local Health District Site Bega Hospital, Bega, NSW Australia; 41 Royal Australasian College of Surgeons, Sydney, NSW Australia; 42 Federal Teaching Hospital, Ido Ekiti, Nigeria; 43 School of Medicine, Marmara University, Istanbul, Turkey; 44 Karadeniz Technical University Farabi Hospital, Trabzon, Turkey; 45 Armidale Rural Referral Hospital, Armidale, NSW Australia; 46 CHU Saadna Abdennour Hospital, Sétif, Algeria; 47 Sydney University, Sydney, NSW Australia; 48 Afe Babalola University Multisystem Hospital, Ado-Ekiti, Nigeria; 49 Autonomous University of Nuevo León, Monterrey, Mexico; 50 George Papanikolaou Hospital, Thessaloniki, Greece; 51 Hospital Universiti Teknologi MARA (HUiTM), Puncak Alam, Selangor Malaysia; 52 University of Tasmania, Hobart, TAS Australia; 53 Center Anti Cancer, Sétif, Algeria; 54 Albury Wodonga Health, Albury, NSW Australia; 55 Armadale Health Service, Perth, WA Australia; 56 Austin Hospital, Melbourne, VIC Australia; 57 Gold Coast University Hospital, Gold Coast, QLD Australia; 58 Goulburn Base Hospital, Goulburn, NSW Australia; 59 Logan Hospital, Logan, QLD Australia; 60 Manning Base Hospital, Taree, NSW Australia; 61 Maroondah Hospital, Melbourne, VIC Australia; 62 Monash Medical Centre, Melbourne, VIC Australia; 63 Orange Base Hospital, Orange, NSW Australia; 64 Women’s and Children’s Hospital, Adelaide, SA Australia; 65 Alexandria Main University Hospital, Alexandria, Egypt; 66 General Hospital of Thessaloniki “George Papanikolaou”, Thessaloniki, Greece; 67 Iaso, Athens, Greece; 68 Larissa University Hospital, Larissa, Greece; 69 Al-Najaf Al-Ashraf Teaching Hospital, Najaf, Iraq; 70 IRCCS Istituto Ortopedico Rizzoli, Bologna, Italy; 71 Alkarak Governmental Hospital, Al-Karak, Jordan; 72 Middlemore Hospital, Auckland, New Zealand; 73 Rotorua Hospital, Rotorua, New Zealand; 74 Dunedin Hospital, Dunedin, New Zealand; 75 North Shore Hospital, Auckland, New Zealand; 76 Abubakar Tafawa Balewa University Teaching Hospital Bauchi, Bauchi, Nigeria; 77 Modibbo Adama University Teaching Hospital, Yola, Nigeria; 78 University of Abuja Teaching Hospital, Gwagwalada, Nigeria; 79 Creek General Hospital, Karachi, Pakistan; 80 Christchurch Hospital, Christchurch, New Zealand; 81 Services Hospital Lahore, Lahore, Pakistan; 82 Nasser Medical Complex, Gaza, Palestine; 83 King Saud Medical City, Riyadh, Saudi Arabia; 84 Hospital Arnau De Vilanova, Valencia, Spain; 85 Al-Moalem Medical City, Khartoum, Sudan; 86 Alandalus Clinic, Elduiem, Sudan; 87 Mugla Training and Research Hospital, Mugla, Turkey; 88 Faculty of Medicine, Trakya University, Edirne, Turkey; 89 Ufuk Üni̇versi̇tesi̇ Tip Fakültesi̇ Dr.Ri̇dvan Ege Sağlik Araştirma Uygulama Merkezi̇ Hastanesi̇, Ankara, Turkey; 90 Lehigh Valley Health Network, Allentown, PA USA; 91 Stanford Health Care, Palo Alto, CA USA; 92 Griffith University, Gold Coast, QLD Australia; 93 Hunter New England Network, Newcastle, NSW Australia; 94 University of Health Sciences, Tepecik Training and Research Hospital, Izmir, Turkey; 95 Texas Tech University Health Sciences Center, Lubbock, TX USA; 96 Central Gippsland Health, Sale, VIC Australia; 97 Azienda Unità Sanitaria Locale di Ferrara-University of Ferrara, Ferrara, Italy; 98 University Hospital of Udine, Udine, Italy; 99 Whangarei Base Hospital, Whangarei, New Zealand; 100 Hebron Government Hospital, Hebron, Palestine; 101 Regional Institute of Oncology Iasi, Iasi, Romania; 102 University of Edinburgh, Edinburgh, UK; 103 Ipswich Hospital, Ipswich, QLD Australia; 104 John Hunter Hospital, Newcastle, NSW Australia; 105 Queen Elizabeth Ii Jubilee Hospital, Brisbane, QLD Australia; 106 The Royal Melbourne Hospital, Melbourne, VIC Australia; 107 General Hospital of Athens “Laiko”, Athens, Greece; 108 Melbourne Training Circuit, Melbourne, VIC Australia; 109 King Edward Medical University Hospital, Lahore, Pakistan; 110 The Canberra Hospital, Canberra, ACT Australia; 111 The Queen Elizabeth Hospital, Adelaide, SA Australia; 112 Royal Prince Alfred Hospital, Sydney, NSW Australia; 113 National Ribat University Hospital, Khartoum, Sudan; 114 Deakin University, Melbourne, VIC Australia; 115 University of Tripoli, Tripoli, Libya; 116 Counties Manukau Health, Auckland, New Zealand; 117 Oncology Center Mansoura University, Mansoura, Egypt; 118 Waikato Hospital, Hamilton, New Zealand; 119 University of Newcastle, Newcastle, NSW Australia; 120 Flinders University, Adelaide, SA Australia; 121 Royal Perth Hospital, Perth, WA Australia; 122 Lyell Mcewin Hospital, Adelaide, SA Australia; 123 Western Health, Melbourne, VIC Australia; 124 Al Basheer Hospital, Amman, Jordan; 125 Education Karima Hospital, Merowe, Sudan; 126 Elduiem Teaching Hospital, Elduiem, Sudan; 127 Atrium Health, Charlotte, NC USA; 128 Agios Savvas Anticancer Hospital of Athens, Athens, Greece; 129 Aga Khan University Hospital, Karachi, Pakistan; 130 Sunshine Coast University Hospital, Birtinya, QLD Australia; 131 Ibrahim Malik Teaching Hospital, Khartoum, Sudan; 132 Abu Saleem Trauma Hospital, Tripoli, Libya; 133 Alkhadra Hospital, Tripoli, Libya; 134 Benghazi Medical Center, Benghazi, Libya; 135 Gharyan Central Hospital, Gharyan, Libya; 136 Ibn Sina Teaching Hospital, Sirte, Libya; 137 Misurata Central Hospital, Misurata, Libya; 138 Tripoli Central Hospital, Tripoli, Libya; 139 Tripoli Medical Center/Tripoli University Hospital, Tripoli, Libya; 140 Zliten Medical Centre, Zliten, Libya; 141 Klaipeda University Hospital, Klaipeda City Municipality, Lithuania; 142 School of Medical Sciences & Hospital USM, Universiti Sains Malaysia, Kota Bharu, Malaysia; 143 University Hospital Dr. Jose Eleuterio Gonzalez, Monterrey, Nuevo Leon Mexico; 144 Hospital Susana Lopez De Velencia, Popayán, Cauca Colombia; 145 Al Istiklal Hospital, Amman, Jordan; 146 Geraldton Regional Hospital, Geraldton, WA Australia; 147 University Hospital Fattouma Bourguiba, University of Monastir, Monastir, Tunisia; 148 Abdulkadir Yuksel State Hospital, Gaziantep, Turkey; 149 University of Otago, Dunedin, New Zealand; 150 The First Affiliated Hospital of Jinan University, Guangzhou, China; 151 Box Hill Hospital, Melbourne, VIC Australia; 152 Bahria International Hospital Lahore, Lahore, Pakistan; 153 Ibn-Sina Hospital, Khartoum, Sudan; 154 Northern Hospital, Melbourne, VIC Australia; 155 Auckland City Hospital, Auckland, New Zealand; 156 Invercargill (Kew) Hospital, Invercargill, New Zealand; 157 Waitakere Hospital, Auckland, New Zealand; 158 Frankston Hospital, Melbourne, VIC Australia; 159 Tanta University Hospital, Tanta, Egypt; 160 Sir Charles Gairdner Hospital, Perth, WA Australia; 161 Western Sydney University, Sydney, NSW Australia; 162 Perth Metro Training Circuit, Perth, WA Australia; 163 Alexandria University, Alexandria, Egypt; 164 Casey Hospital, Melbourne, VIC Australia; 165 Flinders Medical Centre, Adelaide, SA Australia; 166 Hedland Health Campus, Port Hedland, WA Australia; 167 Westmead Hospital, Sydney, NSW Australia; 168 Whyalla Hospital & Health Services, Whyalla, SA Australia; 169 Menofia University Hospital, Shibin el Kom, Egypt; 170 Tauranga Hospital, Tauranga, New Zealand; 171 Royal Australasian College of Surgeons, Adelaide, SA Australia; 172 Khartoum North Teaching Hospital (Bahri Hospital), Khartoum, Sudan; 173 University of Auckland, Auckland, New Zealand; 174 University of Western Australia, Perth, WA Australia; 175 Campbelltown Hospital, Sydney, NSW Australia; 176 Royal Adelaide Hospital, Adelaide, SA Australia; 177 Wyong Public Hospital, Wyong, NSW Australia; 178 Al Tayseer Hospital, Zagazig, Egypt; 179 Assiut University Hospital, Assiut, Egypt; 180 Aswan University Hospital, Aswan, Egypt; 181 Mansoura University Hospital, Mansoura, Egypt; 182 IRCCS Azienda Ospedaliero-Universitaria di Bologna, Bologna, Italy; 183 Princess Basma Teaching Hospital, Irbid, Jordan; 184 University of Ilorin Teaching Hospital, Ilorin, Nigeria; 185 Bashair Teaching Hospital, Khartoum, Sudan; 186 Gadarif Teaching Hospital, Gadarif City, Sudan; 187 Hippokratio General Hospital of Thessaloniki, Thessaloniki, Greece; 188 United Hospital, Karachi, Pakistan

**Keywords:** Pain management, Predictive markers

Correction to: *npj Digital Medicine* 10.1038/s41746-025-01798-6, published online 26 August 2025

In this article, the author list for TASMAN Collaborative consortium was incomplete. The full list of TASMAN Collaborative consortium members and their corrected affiliations have now been provided in the Supplementary Information.

## Supplementary information

Appendix

