# Supplementary material for: Predicting opioid consumption after surgical discharge: a multinational derivation and validation study using a foundation model
Source: NPJ Digit Med. 2025 Aug 26;8:547. doi: 10.1038/s41746-025-01798-6 (PMC12381370; doi:10.1038/s41746-025-01798-6)
Supplement: Supplementary file 2 — Appendix [file 41746_2025_1798_MOESM2_ESM.docx]

**Appendix S1: TASMAN Collaborative**

**Writing group**: *Chris Varghese [University of Auckland, Auckland, New Zealand], †Luke Peters [University of Newcastle, Newcastle, Australia], Lorane Gaborit [Australian National University, Canberra, Australia]; Kaviya Kalyanasundaram [University of Adelaide, Adelaide, Australia]; Aya Basam [Monash University, Melbourne, Australia]; Deborah Wright [University of Otago, Otago, New Zealand]; Jennifer Martin, Melissa Park, **Peter Pockney [University of Newcastle, Newcastle, Australia]; William Xu, Cameron Wells, Gabriel Schamberg, Greg O’Grady [University of Auckland, Auckland, New Zealand], Kenneth A McLean, **Ewen Harrison [University of Edinburgh, UK].

*First author

**Senior author (overall guarantor)

**Statistical analysis:** Chris Varghese*, Gabriel Schamberg, Billy Wu [University of Auckland, Auckland, New Zealand], **Ewen Harrison [University of Edinburgh, UK]

**OPERAS Steering committee**: Aya Basam, Sarah Goh, Jiting Li, Jainil Shah, Abdullah Waraich [Monash University, Melbourne, Australia]; Lorane Gaborit, Upasana Pathak [Australian National University, Canberra, Australia]; Amie Hilder [Deakin University, Melbourne, Australia]; Muhammed Elhadi [University of Tripoli, Tripoli, Libya]; Aiden Jabur [Griffith University, Gold Coast, Australia]; Kaviya Kalyanasundaram [University of Adelaide, Adelaide, Australia]; Christina Ohis [Western Sydney University, Sydney, Australia]; Chui Foong Ong [Melbourne Training Circuit, Melbourne, Australia]; Melissa Park, Venesa Siribaddana [University of Newcastle, Newcastle, Australia]; Kyle Raubenheimer [Perth Metro Training Circuit, Perth, Australia]; Jennifer Vu [Sydney University, Sydney, Australia]; Cameron Wells, Gordon Liu, Liam Ferguson, William Xu, Chris Varghese [University of Auckland, Auckland, New Zealand]

**OPERAS Scientific advisory group:** Peter Pockney, Kristy Atherton, Amanda Dawson, Jennifer Martin [University of Newcastle, Newcastle, Australia]; Arnab Banerjee [Australian National University, Canberra, Australia]; Nagendra Dudi-Venkata [Royal Australasian College of Surgeons, Adelaide, Australia]; Nicholas Lightfoot [University of Auckland, Auckland, New Zealand]; Isabella Ludbrook [Hunter New England Network, Newcastle, Australia]; Luke Peters [Royal Australasian College of Surgeons, Sydney, Australia]; Rachel Sara [Counties Manukau Health, Manukau City, New Zealand]; David Watson [Flinders University, Adelaide, Australia]; Deborah Wright [University of Otago, Otago, New Zealand]

**OPERAS National Leads:** Ademola Adeyeye [Afe Babalola University Multisystem Hospital, Ado-Ekiti, Nigeria]; Luis Adrian Alvarez-Lozada [Autonomous University of Nuevo León, Monterrey, Mexico]; Semra Demirli Atici [University of Health Sciences Tepecik Training and Research Hospital, Izmir, Turkey]; Milos Buhavac [Texas Tech University Health Sciences Center, Lubbock, United States of America]; Giacomo Calini [University Hospital of Udine, Udine, Italy]; Muhammed Elhadi [University of Tripoli, Tripoli, Libya]; Orestis Ioannidis [George Papanikolaou Hospital, Thessaloniki, Greece]; Mustafa Deniz Tepe [Karadeniz Technical University, Trabzon, Turkey]; Upanmanyu Nath [Nilratan Sircar Medical College and Hospital, Kolkata, India]; Ahmad Uzair [King Edward Medical University Hospital, Lahore, Pakistan]; Wah Yang [The First Affiliated Hospital of Jinan University, Guangzhou, China]; Faseeh Zaidi, Surya Singh [University of Auckland, Auckland, New Zealand]; Bahiyah Abdullah [Hospital Universiti Teknologi MARA, Malaysia (HUiTM)], Diana Sofia Garces Palacios* [Hospital Susana Lopez De Velencia], Ahmed Ragab, Ahmed Ahmed [Alexandria University, Alexandria, Egypt]

**OPERAS Australian State Leads:** Kyle Raubenheimer [Royal Perth Hospital, Perth, Australia]; Davina Daudu [University of Western Australia, Perth, Australia]; Sarah Goh, Simran Vinod Benyani, Nandini Karthikeyan [Monash University, Melbourne, Australia]; Laure Taher Mansour [University of Adelaide, Adelaide, Australia]; Warren Seow [University of Adelaide, Adelaide, Australia]; Zoya Tasi [University of Tasmania, Hobart, Australia]; Aiden Jabur [Griffith University, Gold Coast, Australia]; Upasana Pathak [Australian National University, Canberra, Australia]; Melissa Park [University of Newcastle, Newcastle, Australia]

**Algeria**: Dhia Errahmane Abdelmelek*, Ikram Fatima Zohra Boussahel, Oumelaz Kaabache, Naoual Lemdaoui, Oualid Nebbar [Center Anti Cancer, Sétif]; Mounira Rais*, Meriem Abdoun*, Aya Tinhinane Kouicem, Souad Bouaoud, Kamel Bouchenak, Hind Saada, Amel Ouyahia, Wassila Messai [CHU Saadna Abdennour Hospital, Sétif]

**Australia**: Zhi Shyuan Choong*, Clarissa Ting, Michelle Larkin, Pei Jun Fong, Isabel Soh, Alyssia De Grandi, Hareem Iftikhar, Akansha Sinha, Dhruv Kapoor, Tara Chlebicka [Albury Wodonga Health]; David Singer*, Kim Goddard, Lisa Matthews [Armadale Health Service, Mount Nasura]; Rosalina Lin*, Jessica Chambers, Juliet Chan, Brooke Macnab, John Barker, Morgan Mckenzie, Neil Ferguson [Armidale Rural Referral Hospital, Armidale]; Ghanisht Juwaheer*, Vijayaragavan Muralidharan, Sonia Gill, Nakjun Sung, Rohan Patel, Chris Walters, Kevin Nguyen, David Liu, Carlos Cabalag, Jennifer Lee, San-Hui Anita Leow, Suat Li Ng, Hamza Ashraf, Fraizer Mulder, Jonathan Loo, David Proud, Samantha Wong, Yida Zhou, Qi Rui Soh, David Chye, Sean Stevens, Patrick Tang, Stephen Kritharides, Jason Dong, Oscar Morice, Dora Huang, Andrew Hardidge, Mishka Amarasekara, Aleah Kink, Damien Bolton [Austin Hospital, Melbourne]; Alisha Rawal*, Jasraaj Singh*, Matthew Heard*, Yusuf Hassan*, Ahmed Naqeeb, Andrew Cobden, Duron Prinsloo, Dwain Quadros, Emma Gunn, Ha Jin Kim, Jennifer Ekwebelam, James Shanahan, Mustafa Alkazali, Mariyah Hoosenally, Naveen Nara, Peter Nguyen, Sally Barker, Zacchary Tamsett, Naomi Rigby, Hinal Patel, Eloise Ferguson, Lauren Byrnes, Alexander Bravo [Ballarat Base Hospital, Ballarat]; Amie Hilder*, Ally Hui, Antara Karmakar, Bill Wang, Janindu Goonawardena, King Tung Cheung, Nicholas Chan, Ragul Natarajan, Richard Cade, Rong Jin, Shomik Sengupta, Ruth Snider [Box Hill Hospital, Melbourne]; Harsha Morisetty*, Lewis Weeda, Phoebe Sun, Lalitya Chilaka, Jacinta Cover [Bunbury Hospital]; Aashrinee De Silva Abeweera Gunasekara*, Rahavi Senthilrajan, Anas Alwahaib, Alexandra Limmer, Bushra Zamanbandhon [Campbelltown Hospital]; Kumail Jaffry [Casey Hospital, Melbourne]; Yijia Shen*, Alan Chua, Saifulla Syed [Central Gippsland Health]; Sushanth Saha*, John Glynatsis*, Lori Aitchison, Bernard Lagana, Mason Crossman, David Watson, Abby Dawson, Bryan Fong, Ella Harrison, Eleanor Horsburgh, John Glynatsis, Michael Khoo, Kritika Mishra, Lewis Hewton, Alex Mesecke, Hien Tu, Than Tun, Jason Wong [Flinders Medical Centre, Adelaide]; Elynn Ong*, Tara-Nyssa Law*, Ashlee Landy, Alyssa Leano, Andrea Li, Akshay Soni, Benjamin Dowdle, Charles Pilgrim, Dewmi Abeysirigunawardana, Deepak Rajan Jeyarajan, Diya Patel, Jason Chung, Kyle Mckinnon, Madeline Gould, Paul Gilmore, Ruxi Geng, Rachael Loughnan, Sarahjane Norton-Smith, Solomon Nyame, Sarah Tan, Sewni Samarawickrama, Si Woo Yoon, Yantong Wang, Yichi Zhang, Zixuan Wang [Frankston Hospital]; Hans Mare*, Indrajith Withanage [Geraldton Regional Hospital]; Mitali Khattar*, Alexandra Toft, Goutham Sivasuthan, Hailin Zhao, Jordan Addley, Lucinda O’brien, Muhammad Raza, Randipsingh Bindra, Sonakshi Sharma [Gold Coast University Hospital, Southport]; Charlotte Cornwell*, Aditya Patil, Aiden Cheung, Ashleigh Lown, Amanda Dawson, Aneel Blassey, Benjamin Ochigbo, Felicity Cheng, Aleeza Fatima, Edward Zhang, Henry Kocatekin, Charles Roth, Dani Brewster, Kelvin Kwok, Paul Chen, Sharon Laura, Dominic Tynan, Edward Latif, Elizabeth Lun, Elodie Honore, Felix Ziergiebel, Jessica Blake, Karan Chandiok, Katie Bird, Lynette Ngothanh, Melissa Lee, Mariam El-Masry, Peter Hamer, Ramanathan Rm Palaniappan, Richard Mcgee, Sarah Huang, Shane Zhang, Shubhang Hariharan, Yannick De Silva, Celeste Lee, Penelope Fotheringham, Ian Incoll, Timothy Cordingley, Felicity Cheng, Matthew Brown, Leannedra Kang, Rivindu Wijayaratne, Parisse Moore, Gemma Qian, Yara Elgindy [Gosford Hospital, Gosford]; Emma Carnuccio*, Hamish Rae, Mena Shehata [Goulburn Base Hospital]; Mingchun Liu*, Brodee Lockwood, John Van Bockxmeer [Hedland Health Campus]; Ali Alsoudani*, Daniel Swan, Justin Hsieh [Ipswich Hospital]; Francesca Orchard-Hall*, Kai Yun Jodene Tay*, Raagini Mehra*, Alpha Gebeh, Ashley Bailey, Georgia Brown, Ashley Colaco, Hemashree Gopal, Jessica Boyley, Varun Changati, Joseph Fletcher, Tanishq Khandelwal, Colin House, Chris O’neil, Emily Jaarsma, Victor Ly, Zsolt Balogh, Amanda Shui, Vinogi Sathasivam, Hannah Legge-Wilkinson [John Hunter Hospital, Newcastle]; King Ho Wong*, Andrew Chen, Anthony Tran, Peter Rehfisch, Grace Wang, Jonathan Nguyen, Joshua Peker, Kayla Gallert, Mia Komesaroff, Manideep Namburi [Latrobe Regional Hospital]; Elisabeth Goldfinch*, Ropafadzo Muchabaiwa*, Aishwarya Jangam, Isobel Taylor, Iulian Nusem, Jin Hyuk (David) Park, Justin Gundara, Rachael Heigan, Tam Tran, Thomas Mackay, Yasmine Butterworth, Tomas Sadauskas, Melody Tung, Hasthika Ellepola* [Logan Hospital, Meadowbrook]; Christine Gan*, Hakim Fong*, Ankita Das, Leshya Naicker, Samantha Hauptman, Aditi Kamath, Anthea Yew, Anupam Parange, Katie Kim, Sahil Kharwadkar, Tharushi Gamage [Lyell Mcewin Hospital]; Lucille Vance*, Alexandra Seldon, Moheb Ghaly [Manning Base Hospital]; Jainam Shah*, Victoria Phan [Maroondah Hospital, Melbourne]; Karanjeet Chauhan*, Ahmad Bassam, Beverley Vollenhoven, Kumail Jaffry, Kajal Mandhan, Mithra Sritharan, Mahesh Sakthivel, Natalie Evans, Samuel Robinson, Seiyon Sivakumar [Monash Medical Centre, Clayton]; Liberty Marrison*, David Jollow, Krishma Joshi, Steve Tao, Pallavi Shrestha, Sai Keerthana Nukala [Northern Beaches Hospital]; Russell Hodgson*, Anna Crotty, Adriana Esho, Alasdair Harris, Amy Surkitt, Laura Bland, Blake Mcleod, Chonghao Yin, Cambo Keng, Emily Greenwood, Grace Yuan, Emma Haege, Hongyi Wu, Haotian Xiao, Isabella Pozzi, Jeff Fu, Jessica Stott Ross, Juliette Gentle, Kathy Gan, Kelvin Chang, Kexin Sun, Madhavi Singh, Maria Xie, Nicholas Mccabe, Mark Slavec, Nick Clarnette, Behzad Niknami, Peishan Zou, Sean Flintoft, Shenuka Jayatilleke, Rumnea Sok, Suqi Tan, Sanya Wadhwa, Will Swansson [Northern Hospital, Melbourne]; Daniel Abulafia*, Jian Blundell*, Amie Sweetapple, Caitlin Del Solar, Cameron Martin, David Bell, Isuru Fernando, Jared Chang, Katie Vanzuylekom, Katie Van Zuylekom, Kate Van Zuylekom, Katie Hobbs, Richard Liang [Orange Base Hospital]; Aiden Jabur*, Jazmina Tarmidi, Mahmoud Ugool, Nicholas Beatson, Sarah Bowman, Sophie Moin [Queen Elizabeth Ii Jubilee Hospital, Coopers Plains]; Wen Po Jonathan Tan*, Seevakan Chidambaram*, Siang Wei Gan, Pengnan Wang, Leshya Naicker, Katie Kim, Nicole Qiwen Wang, Yi Xin Kwan, Chinmai Patil, Divyanshu Joshi, Aditi Kamath, Aishath Hanan, Arfaan Sheriff, Jaime Duffield, Leshya Naiker, Peter Smitham, Eu Ling Neo, Matthew Chua, Shalvin Prasad, Armitesh Nagaratnam, Tarik Sammour, Yuxin Lin, Christine Lee, Eve Hopping, Muskan Jangra, Ankita Das, Ken Lin, Zachary Bunjo [Royal Adelaide Hospital, Adelaide]; Kyle Raubenheimer*, Mohamed Haseef Mohamed Yunos, Kar Long Yeung, Rachel Phu, Aisling Betts, Benjamin Just, Sahil Gera, Hilary Leeson, Jodie Jamieson, Katie Wang, Emily Luu, Michael Innes [Royal Perth Hospital]; Jennifer Vu*, Jonathan Hong, Stephen Dzator, Aki Flame, Vincent Jiang, Jianing Kwok, Aaron Lawrence, Kate Meads, Liam Pearce, Pavatharane Sarangadasa, Haylee Shaw, Victor Yu, [Royal Prince Alfred Hospital]; Elizabeth Crostella*, James Wong, Sriya Bobba, Maddison Muller, Yin Chi Hebe Hau, Thomas Wilson, Aleksandra Markovic, Jemma Green, Clara Forbes, Emalee Burrows, Lachlan Hou, Clare O’sullivan, Jonathon Foo [Sir Charles Gairdner Hospital, Perth]; Hannah Greig*, A-J Collins, Callum Chandler, Emily Heaney, Hannah Gross, Monica Morgan, Rebecca Loder, Krishnankutty Rajesh [New South Wales Local Health District Site Bega Hospital]; Shravankrishna Ananthapadmanabhan*, Akeedh Razmi, Crystal Vong, Prasanna Pothukuchi, Mary Theophilus, Roshni Sriranjan, Sharon Kaur, Marcelo Kanczuk [St John Of God Midland Public And Private Hospital, Perth]; Julia De Groot*, Angela Corrigan, Damon Li, Danniel Badri, Dominico Ciranni, Elangovan Thaya Needi, Matthew Clanfield, Nicolas Copertino, William Rumble [Sunshine Coast University Hospital]; Maria Kristina Vanguardia*, Chen Lew*, Rami Dennaoui*, Jainil Shah*, Joseph Kong, Imogen Koh, Raymond Zeng, Kristian Baziotis-Kalfas, Hannah Denby, Andy Li, Will Tran, Abhinav Singh, Olivia Lin, Michelle Chau, Olivia Donaldson, Christina (Seojung) Min, Shirahn Ballah, Sonia Ching Ting Tsui, Nathania Yong, Lucy Standish, Sarah Tan, AsukaFujihara, Lily Davies, Ramin Odisho, Anjana Ravi, Josh Collins, Pooja Chandra, Rana Abdelmeguid, GopalSingh, Xireaili Feierdaiweisi, Dharani Seneviratne, Shambhavi Srivastava, Michelle Yao, Cherilyn Teng, Nebula Chowdhury, Sasini Vidanagama, Charles Lin, Tharushi Sampatha-Waduge, Erica Wang, Chatnapa Yodkitydomying, Imogen Koh, Julia Silverii, AaronLam, Raymond Zeng, Krisha Solanki, Angus Franks, Liam Edwards, Ridvan Atilhan, Rohan Nandurkar, Oliver Wells, Kristina Vanguardia, Dennis King, Elton Edwards, Liam Edwards, Quang Tran, Michelle Chau, Seojung Min [The Alfred Hospital, Melbourne]; Abdul Rauf*, Yangzirui Fu*, Hodo Haximolla, Mengge Shang, Sharrada Segaran, Shelley Wang, Gananadha Sivakumar [The Canberra Hospital]; Jaspreet Kaur Sandhu*, Neel Mishra, Samantha Hauptman, Alyssa Chua, Danielle Chene, Guy Maddern, Henry Shaw, Qiwen Wang [The Queen Elizabeth Hospital, Adelaide]; Siyuan Pang*, Christine Lu, James Fung, Kathryn Cyr, Karen Lu, Ming Zhou How, Nelson Hu, Paul Anderson, Philip Jakanovski [The Royal Melbourne Hospital, Melbourne]; Arkan Youssef*, Howard Tang*, Rory Keenan*, Alex Chan, Mitch Canny, Farah Tahir, James Egerton, Justin Yeung, Justin Chan, Lea Tiffany, Michael Bei, Mariolyn Raj, Peter Williams, Sakshar Nagpal, Tim Outhred, Russel Krawitz, Colin Chan-Min Choi [Western Health, Melbourne]; Khadijah Younus*, Mary Giurgius*, Rosemary Kirk, Amanda Gonzalez Pegorer, Pattarapan Tang-Ieam, Jack Ward, Asanka Wijetunga, Caitlin Zhang, Chris Nahm, Christine Wang, Damian Golja, Gregory Jenkins, Helena Qian, Jason Luong, Kim Nguyen, Sean Suttor, Sherman Lai, Vanessa Ma, Yan Chen [Westmead Hospital, Westmead]; Hoi Hang Yu*, Amos Lee, Antonio Barbaro, Cameron Mcguinness, Guy Maddern, Stevie Young [Whyalla Hospital & Health Services, Whyalla]; Ye Fang Lim*, Georgina Trotta, Phoebe Chao, George Ding, Carol Fang, Andi Lu, Prabhath Wagaarachchi [Women's And Children's Hospital]; Charlotte Cornwell*, Amy Gojnich, Peter Stewart, Isabella Dong, Kenneth Wong, Luca Burruso, Lucinda Hogan, Nathan Mcorist, Ramnik Singh, Ragavi Jeyamohan, Zhen Hou, William Lai, Emily Taylor [Wyong Public Hospital, Wyong]

**Colombia:** Diana Sofia Garces Palacios*, Maria Alejandra Nanez Pantoja, Daniel Mauricio Bolanos Nanez, Gilmer Omar Perez Hernandez, Lia Jasmin Jimenez Ramirez [Hospital Susana Lopez De Velencia]

**Egypt**: Mohamed S. Mohamed*, Ahmed Kamal El-Taher, Ahmed Elewa, Mahmoud Ayman Soliman, Menna Diab, Radwa Ali [Al Tayseer Hospital, Zagazig]; Ahmed Ahmed*, Adham Galal, Ahmed Elkhodary, Ali Alaa, Arwa Faisal, Asmaa Badawy, Donia Eldomiaty, Mohamed Al Sayed, Esraa Rasslan, Mohamed Ramadan, Gamal Elsayed Fares, Hashem Altabbaa, Humam Emad, Muneera Alboridy, Mahmoud Mongy, Osama Albarhomy, Osama Selim, Rawan Rafaei, Raneem Atta, Ahmad Altaweel, Yara Sherif, Youssef Elghoul, Yousef Tarek, Ahmed Abdelfatah Sabry, Ahmad Moustafa, Osama AbouHiekal, Osama Al Shaqran, Zeyad Haggag [Alexandria Main University Hospital, Alexandria]; Ahmed M. Abbas*, Abdallah Rashad Temerik, Dina Atef*, Ahmed F. Abdelkawi, Ahmed A. Youssef, Mohamed M. Abdallah, Ahmed Mahmoud, Mahmoud M. Saad*, Mohamed Ragab, Aya Hussien, Mostafa Abdelbaky, Ismail Muhammad, Afnan Morad, Ahmed Ali, Ahmed Hussien, Ahmed Shipa, Ahmed Aboulfotouh, Ahmed M. Kamel, Ahmed Mohamed Hashem, Ahmed M. Abdelaal. Ahmed Morsi, Alshymaa Ebrahim, Amira Gad el-mola, Ahmed Mohamed Sayed, Amira Abdelrahman, Ali Momen Kamel, Abdallah Elmaghrabey, Aml Ali, Samah Abdelnaeam, Asmaa Emam, Aya Shaban, Asmaa S. Shaltout, Bashayer Nabil, Fady Barsoum, Esraa Mostafa, Doaa Salah, Doaa Abdelbaset, Rehab Ahmed, Dina Othman, Safaa Othman, Nour Salah Khairallah, Salma Morsi, Shimaa Abbas Hassan, Ahmed Abuelsoud Abden, Armia Azer, Eman S. Shaltout, Enas Abdelbaset Abdelsamed, Islam Ibrahim, Esraa Abdelbaset, Esraa Hamoda, Fatma Monib, Fatma Harb, Hatem Ahmed, Hager Maher, Haitham Mohammed, Kerolous Hana, Kerillos Ayoub, Kerollos Henes, Keroles Soliman, Kerollos Shamshoon, Mahmoud Hassanein, Magdy Mahdy, Mohamed M. Abdelhamid, Mahmoud Khalil, Manal Ali, Mostafa M. Elsonbaty, Mansour Khalifa, Marwa Amary, Merna Ezz Suliman, Mohammed Saif Al Nasr, Mohammed Nagy Elammary, Michael Elia, Michael Adly, Moahmed Zaed, Mohamed M. Hares, Mo'men Roshdy, Mohammed Al-Quossi, Mohammed Fargaly, Mohamed F. Ramadan, Mohammed A. Shahat, Mona Saber, Mohammad K. Abdelnasser, Mohammed Abdelnasser Abdelfatah, Mahmoud Abughanima, Mahmoud Abdelgaber, Mostafa Abbas, Mona Saber, Mostafa K. Amin, Ola Haroon, Omima Khalil, Omnia Talaat, Rahma Elnagar, Randa Soliman, Reham Aboelela, Salem Salah, Samia Abdelgawad, Tasneem Mohammed, Tarek Hussien, George Sobhy, Yasmeen Sayed, Yousra Othman [Assiut University Hospital, Assiut]; Reham Mahmoud Silem *, Ali Dawood, Tarek Hemaida, Reem Ahmed, Aya Kamal, Mohamed Salah, Ahmed Zaharia [Aswan University Hospital, Aswan]; Ebrahim Salem, Osama Fathy Ali Ali Rashed, Mohamed Halawa [El Tadamon Specialised Hospital, Portsaid]; Hossam Elfeki*, Abdelrahman Mosaad, Abdelrahman Shaaban, Hebatalla Abdelsalam, Ahmed Sakr, Aly Sanad, Amr Elsawy, Bassant Maged Maged, Dana Hegazy, Mohamed Abdelmaksoud, Mahmoud Laymon, Mohamed Taman, Esraa R Moawad, Hadeer Elsaeed AboE lfarh, Karim Elkenawi, Manar Osama, Mirna Sadek, Mohamed Abdelaziz Elghazy, Mohamed Attia Elfadali , Mohamed Nader, Mostafa Shalaby, Omar Attiya, Osama Samir Gaarour, Ahmed Zaghloul [Mansoura University Hospital, Mansoura]; Pola Mikhail*, Karim Badr, Hatem Soltan, Mohamed Donia, Mohammed Gaafar [Menofia University Hospital, Menofia]; Khaled Abdelwahab*, Abdelaziz Sallam, Ahmed Eid, Mohamed Yousri, Omar Hamdy [Oncology Center Mansoura University, Mansoura]; Aiman Al-Touny*, Abdelrhman Alshawadfy, Ahmed Hamdy, Ahmed Ellilly, Ahmed Mahdy, Ahmed El-Sakka, Hamdy Hendawy, Asmaa Salah, Bassma Raslan, Eman Teema, Eslam Albayadi, Esraa Nasser, Hanaa Mohamed, Mohamed Mahmoud, Mostafa Elsaied, Omima Taha, Shaimaa Dahshan, Shimaa Al-Touny, Ahmed Karrar, Ahmed Khairy, Abdelrahman Farag, Asmaa Deafallah [Suez Canal University Hospital, Ismailia]; Alaa Mohamed Ads*, Rabiaa Alomar, Issa AbuShawareb, Abdallah Saeed, Abdelhafeez Mashaal, Adel Mohamed Ads, Sohila Ghanem, Ahmed Elghamry, Eman Ayman Nada, Youssef Ali Noureldin, Mohamed Fayez Fouda, Nourhan Shaheen, Shereen Allam, Ibrahim Mazrou, Ali Fahmy Shehab, Wesam Kussaili [Tanta University Hospital, Tanta]

**Greece**: Dimitrios Korkolis*, Evangelos Fradelos, Aikaterini Sarafi [Agios Savvas Anticancer Hospital Of Athens]; Nikolaos Machairas*, Konstantinos S. Giannakopoulos, Fotios Stavratis, Georgios Korovesis, Gerasimos Tsourouflis, Myrto D. Keramida, Nikolaos Kydonakis, Stylianos Kykalos, Athanasios Syllaios, Panagiotis Dorovinis, Dimitrios Schizas [General Hospital Of Athens "Laiko"]; Orestis Ioannidis*, Anastasia Malliora, Elissavet Anestiadou, Konstantinos Zapsalis, Fotios Kontidis, Lydia Loutzidou, Nikolaos Ouzounidis, Stefanos Bitsianis, Savvas Symeonidis, Smaragda Skalidou, Orestis Ioannidis, Olga Maria Valaroutsou [General Hospital Of Thessaloniki "George Papanikolaou"]; Themistoklis Dagklis*, Alexandra Arvanitaki, Apostolos Mamopoulos, Apostolos Athanasiadis, Stergios Kopatsaris, Ioannis Kalogiannidis, Ioannis Tsakiridis, Georgios Kapetanios, Evangelos Papanikolaou, Nikolaos Tsakiridis, Fotios Zachomitros [Hippokratio General Hospital Of Thessaloniki]; Andreas Larentzakis*, Argyrios Gyftopoulos, Konstantinos Albanopoulos, Apostolos Champipis, Christos Yiannakopoulos, Gavriella Zoi Vrakopoulou, Konstantinos Saliaris, Konstantinos Lathouras, Spyridon Skoufias, Georgia Doulami [Iaso]; Metaxia Bareka*, Eleni Arnaoutoglou, Fragkiskos Angelis, Fragkiskos Angeslis, Michael Hantes, Maria Ntalouka [Larissa University Hospital]

**Iraq**: Maytham A. Al-Juaifari*, Mohammed Alwash, Rasool Maala, Yasir Adnan Zwain, Sara Ahmed Saleh, Mohammed Khorsheed [Al-Najaf Al-Ashraf Teaching Hospital, Najaf]

**Italy**: Antonio Pesce, Carlo V. Feo*, Massimiliano Bernabei*, Francesca Petrarulo, Nicolò Fabbri, Raffaele Labriola, Silvia Jasmine Barbara [Azienda Unità Sanitaria Locale di Ferrara-University of Ferrara, Ferrara]; Simone Bosi*, Angela Romano, Anna Canavese, Caterina Catalioto, Claudio Isopi, Cristina Larotonda, Gerti Dajti, Matteo Rottoli, Iris Shari Russo, Stefano Cardelli [IRCCS Azienda Ospedaliero-Universitaria di Bologna, Bologna]; Francesco Castagnini*, Francesco Traina, Giulia Guizzardi, Giulia Giuzzardi, Mara Gorgone, Marco Maestri [IRCCS Istituto Ortopedico Rizzoli, Bologna]; Pasquale Cianci*, Ivana Conversano, Enrico Restini, Domenico Gattulli, Giorgia Grillea, Marco Varesano [Hospital Lorenzo Bonomo, Andria]; Giacomo Calini*, Adelaide Andriani, Davide Gattesco, Giovanni Terrosu, Mattia Zambon, Pietro Matucci Cerinic, Luisa Moretti, Davide Muschitiello, Samantha Polo, Vittorio Bresadola [University Hospital Of Udine, Udine]

**Jordan**: Salah Abu Wardeh*****, Mahmoud Al-Baw*, Saif Alhaleeq*, Subhi Al-Issawi*, Abdalqader Al Smadi, Esmat Alsaify, Farah Banihani, Noor Massadeh, Nada Massadeh, Dima Al-issawi, Basel Elyan, Qotadah Al-Shami, Yazan Alomari, Almu’atasim Khamees, Sief-Addeen Al-Tahayneh, Ahmad Malek Alsheikh, Khaled Ahmad Sawaftah, Osama Abdul kareem Sarhan [Al Basheer Hospital, Amman];; Abed Alazeez Alkhatib*, Bader Alzghoul, Ahmad Saleh, Jamal Yaghmour, Mahmoud Shahin, Mohammed Maali [Al Istiklal Hospital, Amman]; Dawood Alatefi*, Heba Al-Smirat, Abdulhakim Hezam, Nassar Alathameen [Alkarak Governmental Hospital, Alkarak]; Amr Al Hammoud*,Abdulrahim Al Kaddah*, Salem Ayasrah, Hamza Abuuqteish, Tesneem Al-Mwajeh, Reena Makableh, Saad Bataineh, Amin Shabaneh, Wesam Alnatsheh, Marwan Aldeges, Huda Hamad, Sireen Shehahda, Dima Khassawneh, Osama Alzyoud, Risan Alrosan, Hasan Awad, Tariq Khaldoon, Rabab Shannaq, Mohammad Al hamoud, Bader Abo fadalah, Mo'ath Al-Hazaimeh, Wail Khraise [King Abdullah University Hospital, Irbid]; Lara Alnajjar*, Majjd Alnajjar*, Sohaib Al-Omary*, Adnan Ababneh, Alaa Albashaireh, Mohammad Khadrawi, Mohammad Aljamal*, Tayseer Athamneh, Ro-a Muqbel, Maryam Al-jammal, Ahmad Masarrat, Alia Al-zawaydeh, Ibrahim Taha, Taima’ Qattawi, Rayyan Smadi, Ayah Alhaleem, Mosab Alboon, Omar Hazaymeh, Leen Karasneh, Safa’ Al-Haek [Princess Basma Teaching Hospital, Irbid]

**Libya**: Marin Almahroush*, Tamam Alfrijat, Aya Elporgay, Hadeel Shanag, Hamza Agilla, Hind Alameen, Marya Bensalem, Mawadda Altair, Malak Ghemmeid, Rehab Alarabi, Sara Alhudhairy [Abu Saleem Trauma Hospital, Tripoli]; Rima Gweder*, Amal Alzarroug, Ebtihal Alabed, Fadwa Elreaid, Omar A Elkharaz, Fatma Fathi Elreaid, Safa Sasi Albatni [Alkhadra Hospital, Tripoli]; Haitham Elmehdawi*, Milad Gahwagi*, Ayman Mohamed, Tariq Alfrjani, Khaled Khafifi, Ayat H Rasheed, Ayoub Akwaisah , Hassan Bushaala, Mustafa Elfadli, Mohamed Moftah, Salima Algabbasi, Salma Esaiti, Sara Elfallah, Abtisam Alharam, Fatima Alariby, Mohamed Isweesi, Tarik Ahmed Eldarat, Ayman Arhuma Dabas [Benghazi Medical Center, Benghazi]; Akram Alkaseek*, Ahmed Mohammed Abodina, Aya Alqaarh, Hibah Bileid Bakeer, Hoda Salem Alhaddad, Husein Aboudlal, Sawsan Alsaih [Gharyan Central Hospital, Gharyan]; Noora Abubaker, Najwa Abdelrahim*, Ali Alzarga, Basma Omar, Farah Faris, Qamrah Alhadad [Ibn Sina Teaching Hospital, Sirt]; Asma Abufanas*, Hussameddin Badi*, Israa Benismai*, Hawa Obeid*, Abdulwahab Abdalei, Ahmed Abdulrahman, Aisha Swalem, Ebtisam Alzarouq, Amna Safar, Esra Shagroun, Boshra Hashem, Fatheia Elrishi, Fatima Abdulali, Habeeba Ahmed, Ibrahim Eltaib, Joma Elzoubia, Aisha Albarki, Hoda El Mugassabi, Fatima Abushaala, Amany Abuzaho, Nida Juha, Raneem Egzait, Sundes Shetwan, Alzahra Lemhaishi, Faisel Matoug [Misurata Central Hospital, Misurata]; Eman Abdulwahed*, Aamal Askar, Abir Ben Ashur, Adel Bezweek, Bushra Altughar, David Emhimmed, Donia Elferis, Laila Elgherwi, Enas Soula, Doaa Gidiem, Maren Grada, Khawla Derwish, Maram Alameen, Nassib Algatanesh, Ahlam Elkheshebi, Reem Ghmagh, Sharf Barka, Sultan Ahmeed, Sarah Aljamal, Zahra Alragig, Mohamed Addalla, Ahmed Atia, Atab Kharim, Fathia Mahmoud, Muhannud Binnawara, Entisar Alshareea [Tripoli Central Hospital, Tripoli]; Mohamed Alsori*, Aisha Alshawesh, Ghaliya Mohamed H Alrifae, Amira Ashour, Anwaar Abozid, Asil Omar Saleh Alflite, Anwar Mohamed, Jaber Arebi, Fatma Alagelli, Hana Yousef Gineeb, Rawia Ghmagh, Rihab Mohammed Bin Omar, Retaj Alaqoubi, Sara Mohammed, Serien Hossain Bensalem, Tahani Elgadi, Wesam Sami, Yara Bariun, Abdulhadi Mohammed Alhadi Alhashimi, Dheba Almukhtar Abdulla, Heba Rhuma, Husam Enaami, Asraa Ali Alboueishi [Tripoli Medical Center/ Tripoli University Hospital, Tripoli]; Hayat Ben Hasan*, Mohamd A A Alkchr, Bashir Albakosh, Najah Alsari, Mahammed Aldreawi, Najat Ben Hasan, Khaled Abushanab, Rawad Yahya [Zliten Medical Centre, Zliten]

**Lithuania:** Narimantas Samalavicius*, Vitalijus Eismontas, Jonas Jurgaitis, Oleg Aliosin, Vitalija Nutautiene [Klaipeda University Hospital]

**Malaysia:** Andee Dzulkarnaen Zakaria*, Anil Kumar Sree Kumar Pillai, Dinesh Kumar Vadioaloo, Mohamed Ashraf Mohamed Daud, Jien Yen Soh, Mohd Zaim Zakaria [School of Medical Sciences & Hospital USM, Universiti Sains Malaysia]; Siti Mayuha Rusli*, Nur Ayuni Khirul Ashar*, Zatul Akmar Ahmad*, Afiq Aizat Ramlee, Sharifah Nor Amirah Syed Abdul Latiff Alsagoff, Ahmad Anuar Sofian, Muhammad Badrul Hisyam Mohamad Jamil, Bahiyah Abdullah, Mohamad Faiz Noorman, Muhammad Fihmi Zainal Abidin, Mohamed Izzad Isahak, Siti Nasyirah Nisya Adnan, Zaidatul Husna Mohamad Noor, [Hospital Universiti Teknologi Mara (HUiTM)]

**Mexico**: Luis Adrian Alvarez-Lozada*, Alejandro Quiroga Garza, Andrea Aguilar Leal, Bernardo Alfonso Fernández Reyes, Ethel Valeria Orta Guerra, Francisco Javier Arrambide Garza, Héctor Erasmo Alcocer Mey, Jorge Arath Rosales Isais, Juventino Tadeo Guerrero Zertuche, Patricia Ludivina González García, Luis Antonio Heredia Sánchez, Marcela Patricia Flores Mercado, Oscar Alonso Verduzco Sierra, Pedro Emiliano Ramos Morales, Stephie Oyervides Fuentes, Víctor Manuel Peña Martínez, Yesika Alejandra Guerra-Juárez, Ana Karina Flores-González [University Hospital Dr. Jose Eleuterio Gonzalez, Monterrey, Nuevo Leon]

**New Zealand**: Surya Singh*, Arwa Hadi, Christian Woodbridge, David Thornton-Hume, Jack Forsythe, Isini Dharmaratne, Vivian Pai, John Windsor, Kamran Zargar, Lucy Waldin, Lily Winthrop, Matias Alvarez, Meileen Huang, Matt Kumove, Marta Simonetti, Namisha Chand, Oliver Goldsmith, Oscar Guo, Paul Monk, Karen Zhou, Sai Harshitha Penneru, Shaamnil Prasad, Seifei Ren, Terrence Hill, Vyoma Mistry, Selena Sun [NZ, Auckland, Auckland City Hospital]; Ashley Pereira*, Scott Mclaughlin*, Andrew Stokes, Avinash Sathiyaseelan, Jeremy Rossaak, Janice Lim, Kenya Brooke, Liam Quinlan, Mark Pottier, Nayanika Podder, Puja Jinu, Shanay Ramphal, Wikus Vermeulen, Flavio Ordones [NZ, Bay Of Plenty, Tauranga Hospital]; Fraser Jeffery, Ibrahim S. Al Busaidi, Janelle Divinagracia, William Ju, Yizhuo Liu, Tamara Glyn, Nasya Thompson* [NZ, Canterbury, Christchurch Hospital]; Vivien Graziadei*, Joshua Canton*, Joseph Furey*, Horim Choi, Grace Coomber, Tanya Divekar, Tessa English, Erin Gernhoefer, Tom Healy, Justin Chou, Dikshya Parajuli, Catherine Reed, Rod Studd, Anthony Lin [NZ, Capital And Coast, Wellington Hospital]; Cameron Wells*, Cindy Xu*, Arwa Hadi, Andrew Maccormick, Heejun Park, Athulya Rathnayake, Brittany Williams, Ashley Chan, Corinne Smith, Francesca Casciola, Jainey Bhikha, Jonathan Luo, Kevin Yi, Megan Singhal, Ria George, Rosie Luo, Taylor Frost [NZ, Counties Manukau, Middlemore]; Fatima Hakak*, Akhita George, Angela Carlos, Annie Ho, Connor Mcrae, Jonathan Lescheid, Jenny Soek, Andrew Pham, Sophie St Clair, Su-Ann Yee, Jennifer Lim, Chun-Yen Wu [NZ, Lakes, Rotorua Hospital]; Taehoon Kim*, Anne Qi Chua, Christopher Harmston, Hamish Boyes, Holly Cook, Jamie Struthers, Jess Radovanovich, Nicholas Quek [NZ, Northland, Whangarei Base Hospital]; Chekodi Fearnley-Fitzgerald*, Deborah Wright, Kushan Ghandi, Natalie Matheson [NZ, Southern, Dunedin Hospital]; Matthew James McGuinness*, Brian Chen, Rebecca Indiana Douglas, Konrad Richter, Nisha Bianca Soliman, Scott Matthew Bolam, Vineeth Vimalan, William Currie [NZ, Southern, Invercargill (Kew) Hospital]; Mitchell Cuthbert*, Poppy Ross*, Amy Nicholson, Briar Garton, Emilie Agnew, Niamh Conlon, Nicholas Waaka, Ritwik Kejriwal, Sean Nguyen, Edmund Leung [NZ, Taranaki, New Plymouth Hospital]; Milidu Ratnayake*, Quintin Smith*, Nejo Joseph*, Bosco Yue, Calvin Fraser, Charles Lam, Ethan Figgitt, Gordon Liu, Kevin Tan, Ha Seong You, Helen Zheng, Jenny Luo, James Sharp, Kabir Khanna, Levi Simiona, Michel Luo, Milidu Ratnayake, Patrick Wong, Rebecca Luu, Rohit Paul, Shiva Nair, Shadie Asadyari-Lupo, Wing Hung, Geoffrey Ying [NZ, Waikato, Waikato Hospital]; Jess Ho*, Alan Wu, Eamon Walsh, Jouyee Lee, Jessie Liu, Sunny Yao, Omar Nosseir, Jennifer Dang, Simon Young, Sof'ya Zyul'korneeva, Theresa Boyd [NZ, Waitemata, North Shore Hospital]; Jess Ho*, Alan Wu, Sunny Yao, [NZ, Waitemata, Waitakere Hospital]

**Nigeria**: Abdullahi Musa Kirfi*, Adamu Bala Ningi, Mohammad Albuhari Garba, Makama Baje Salihu, Ohia Ernest Ukwuoma, Abdullahi Ibrahim, Isa Mienda Sajo, Muhammad Baffah Aminu, Liman Haruna Usman, Oloko Nasirudeen Lanre, Ibrahim Shaphat Shuaibu, Stephen Yusuf, Tiamiyu Ismail, Gabi Ibrahim Umar [Abubakar Tafawa Balewa University Teaching Hospital Bauchi, Bauchi]; Ademola Adeyeye*, Ehis Afeikhena, Favour Chinenye Nnaji, Joy Onyekachi Agu, Temiloluwa Peace Maxwell, Oluwatosin Olakunle Motajo, Oghenekaro Ifoto, Seubong-Abasi Imoh Okon [Afe Babalola University Multisystem Hospital, Ado Ekiti]; Jerry Godfrey Makama*, Amina Abosede Mohammed-Durosinlorun, Bashiru Aminu, Polite Iwedike Onwuhafua, Caleb Mohammed, Lubabatu Abdulrasheed, Joel Amwe Adze, Khadijah Richifa Suleiman, Lydia Regina Airede, Mathew Chum Taingson, Stephen Bodam Bature, Stephen Akau Kache, Uchechukwu Ohijie Ogbonna [Barau Dikko Teaching Hospital, Kaduna]; Mohammed Bello Fufore*, Abdulkarim Iya, Adeshina A Ajulo, Ahmad Mahmud, Bilal Shuaibu Yahya, Farida Onimisi-Yusuf, Hope Isaac, Timothy Jawa, Fashe Joseph, Bemi Kala, Maisaratu A Bakari, David Wujika Ngwan, Abubakar umar, Abraham L Filikus, Daniel Wycliff [Modibbo Adama University Teaching Hospital, Yola]; Abiodun Okunlola*, Olukayode Abiola, Adebayo Adeniyi, Olabisi Adeyemo, Babatunde Awoyinka, Olakunle Babalola, Adewumi Bakare, Taiwo Buari, Cecilia Okunlola, Gbadebo Adeleye, Adedayo Salawu, Henry Abiyere, Adetolu Ogidi, Tesleem Orewole [Federal Teaching Hospital, Ido Ekiti]; Habiba Ibrahim Abdullahi*, Godwin Akaba, Arome Achem, Asi-oqua Bassey, Emeka Ayogu, Bilal Sulaiman, Dennis Anthony Isah, Chukwunonso Nnamdi Akpamgbo, Felicia Asudo, Nathaniel Adewole, Omachoko Oguche, Peter Ejembi, Samuel Ali Sani, Paul Chimezie Andrew, AliyuYabagi Isah, Bolarinwa Eniola, Zumnan Songden, Teddy Agida, Terkaa Atim [University Of Abuja Teaching Hospital, Gwagwalada]; Taofiq Olayinka Mohammed*, Hadijat Olaide Raji*, Femi Ibiyemi, Hafeez Salawu, Olushola Fasiku, Remi Sanyaolu Solagbade, Mariam Motunrayo Shiru, Gbadebo Hakeem Ibraheem, Justina Oruade, Grace Ezeoke [University Of Ilorin Teaching Hospital, Ilorin]

**Pakistan**: Tabish Chawla*, Aliya Begum Aziz, Anoosha Marium, Ayesha Akbar Waheed, Faiqa Binte Aamir, Faiza Qureshi, M Hammad Ather, Iqra Fatima Munawar Ali, Izza Tahir, Maha Ghulam Akbar, Ronika Devi Ukrani, Sajjan Raja, Sehar Salim Virani, Shahryar Noordin, Saif Ur Rehman, Shalni Golani, Syed Roohan Aamir, Syed Musa Mufarrih, Usama Waqar, Maliha Taufiq [Aga Khan University Hospital]; Ahmed Siddique Ammar*, Adya Ejaz*, Albash Sarwar, Ahmed Usman Khalid, Shehrbano Khattak [Bahria International Hospital Lahore]; Aliza Imran, Omer Bin Khalid, Urauba Kaleem, Urwah Muneer, Yumna Kashaf [Creek General Hospital]; Fatima Zafar*, Adil Zaheer, Muhammad Ali, Amna Shafaat, Arisha Qazi, Asjad Imran,Mahnoor Tariq, Muhammad Nadeem Aslam, Shehroz Ali, Tabish Atiq, Tayyiba Wasim, Daniyal Babar, Ahmad Zain, Muhammad Ibtisam [Services Hospital Lahore]; Uzair Ahmed, Syed Talha Bin Aqeel, Muhammad Muhib, Muhammad Anas Abbal, Nasar Ahmad Khan, Imran Javed [United Hospital]

**Palestine**: Layth Al-Karaja*, Dana Amro, Ghaida Manasrah, Ibraheem Hammouri, Ihab Abu Hilail, Jihad Zalloum, Laith Alamlih, Mahmoud Nasereddin, Munia Rajabi, Sa’ed Shalalfeh, Zeinab Natsheh [Hebron Government Hospital, Hebron]; Khamis Elessi*, Mustafa Abu Jayyab*, Mohammed Astal, Mosheer Al-Dahdouh [Nasser Medical Complex, Gaza]; Alaa Eddin Salameh*, Alaa Ayyad, Nimatee Dawod, Hamza Alsaid, Iyas Matar, Majd Hassan, Mohammed Bakeer, Mohammad Malasah, Shehab Abuhashem, Mohammed Salem, [Palestine Medical Complex, Ramallah]

**Romania**: Sorinel Lunca*, Mihail Gabriel Dimofte, Stefan Morarasu, Ana Maria Musina, Cristian Ene Roata, Natalia Velenciuc [Regional Institute Of Oncology Iasi, Iasi]

**Russia:** Aleksandr Butyrskii*, Maxim Bozhko, Amet Ametov [Emergency Municipal Hospital No.6]

**Saudi Arabia**: Sharfuddin Chowdhury*, Doaa Bagazi [King Saud Medical City, Riyadh]

**Spain:** Julio Domenech*, Alejandro Rosello-Añon, Ana Monis, Caterina Chiappe, Beatriz Cuneo, Pablo Clemente-Navarro, Jorge Febre, Jorge Sanz-Romera, Marcos Lopez-Vega, Ignacio Miranda, Rocio Valverde-Vazquez, Sara Garcia, Maria Jose Sanguesa, Zutoia Balciscueta [Hospital Arnau De Vilanova]; Enrique Ruiz*, Eduardo Marco, Elena Talavera, Joan Farre, Loreto Bacariza, Mireia Duart, Violeta Ureña, Xenia Carre [Hospital Sant Joan Reus]

**Sudan**: Hytham K. S. Hamid*, Montasir A. Abd-Albain, Sami Galal-Eldin [Al-Moalem Medical City]; Monira Sarih*, Eithar Adam, Samir Ismail, Malaz Azhari, Tawfieg Hassan [Alandalus Clinic, Elduiem]; Mohamed Salaheldein*, Zainab Abdalla, Wahiba Ahmed [Bashair Teaching Hospital, Khartoum]; Monzer Abdulatif MohamedAlhassan Mohamed *, Hozifa Mohamed Abdalla Suliman, Mohammed Omer Mohammed Eltayeb [ Karima Teaching Hospital, Merowe]; Rogia Ahmed Abdalla Ahmed*, Enas Mohammedtom Abdulhameed Babekir, Munya Ali Talab Khairy, Maha Mukhtar Ahmed Mukhtar, Rzan Ali Hamedelneel Ali, Yasir Babkir Ali Al-Shambaty [Elduiem Teaching Hospital, Elduiem]; Fatima Imad Yousif*, Hawa Mohammed Hassan Mohammed, Lana Osher, Lana Osher , Menhag Abdelbast, Mohamed Yassin, Noon Moawia, Rowa Abdalsadeg [Gadarif Teaching Hospital, Gadarif City]; Abrar Husein, Baraa Elhassan, Alnazeer Y. Abdelbagi, Mohammed A. Adam, Eithar M. Ali, Ibrahim A.b. Mohammed, Maab Mohamed, Mohamed Abdulaziz, Mazin Akasha, Muaz Hassan, Nadir Hilal, Noon Abdalla Abdelrahman Mohamed, Noora Abubaker, Omeralfarouk Mohammed, Shakir Mohamed, Walaa Osman, Fatima Mustafa, Alaa A Salih [Ibn-Sina Hospital, Khartoum]; Doua Ali*, Doha Mohammed Ahmed Almakki, Hanan Elnour Mohamed, Abdelhadi Elmubark, Mohamed Hassan, Ammar Alnour, Amna Elaagib, Ayman Abdelrahman, Mubarak Abdelkhalig, Khalid Nour Eldaim, Afra Babiker, Entisar Ahmed, Maab Ali, Eman Hussain, Mansour Wedatalla, Alaaaldeen Ahmed, Alla Aldeen Hamza, Mohab Mohammed, Omer Osman, Reham Ibrahim, Rihab Ahmed, Ruaa Ahmed, Ruaa Yasir, Safaa Awadallah, Sara Mohmmed, Suhaib Hassan [Ibrahim Malik Teaching Hospital, Khartoum]; Walid Shaban*, Aisha Hussein, Reem Rafea, Ahmed Abdalla, Abdalla Ahmed, Khalid Mohamed, Mansour Mohammed, Mohamed Altahir, Mohammed Adam, Omer Mohamed, Walaa Abdullah [Khartoum North Teaching Hospital (Bahri Hospital)]; Hammad Fadlalmola*, Ahmed Yassir Abdalla, Ahmed Ali Omer, Ahmed Alfatih Mustafa, Rawan Elnoman Elhadi, Essam Eldien Abuobaida Banaga, Fatima Osman, Mohamed Galal Ali Abdalla, Hala Abdelhalim Mohamed Taha, Noon Ezzeldien Abdalmahmoud, Rofuida Hussien Nafie, Sami Jamal, Sharwany Ahmed, Doha Amir AtaAlmanan [National Ribat University Hospital, Khartoum]

**Syria**: Rawan Alsheikh Ali*, ‏Abdallah ‏Aladna, ‏Abdullah ‏Aljoumaa, ‏Hamdi ‏Nawfal, Salma Jamali, ‏ Fatima Khouja, ‏Ammar ‏Niazi, Toka ‏ ‏Al Rawashdeh [Aleppo University Hospital, Aleppo]

**Tunisia:** Nahla Kechiche*, Mouna Gara, Mouna Nasr, Marwen Baccar, Oumayma Benamor, Sawssen Chakroun [University Hospital Fattouma Bourguiba, University Of Monastir]

**Turkey**: Ahmet Necati Sanli*, Ahmet Yildiz, Mehmet Ali Demirkiran, Yildiz Buyukdereli Atadag, Yusuf Iskender Tandogan [Abdulkadir Yuksel State Hospital]; Esin Ozkan*, Yıldırım Ozer, Esin Ozkan, Muhammed Miran Oncel, Senad Kalkan [Bezmialem Vakif University, Faculty Of Medicine, Istanbul]; Tolga Gover*, Berke Manoglu, Ilayda Oksak, Ipek Kurt, Kerem Rifaioglu, Selman Sokmen, Tayfun Bisgin, Yasemin Yildirim, Abdil Yetkin Keskin [Dokuz Eylul Univ. Hospital, Izmir]; Tugce Dogan*, Berfin İlgaz Sahin, Cemil Aydin, Duygu Ece Benek, Hale Nur Tiras, Mert Arslangilay, Mert Aslangilay, Muhammet Yaytokgil, Mehmet Ali Capar, Yasemin Yazgan [Hitit University Faculty Of Medicine Çorum Research And Training Hospital]; Sebnem Bektas*, Ahmet Can Alagoz, Alara Ece Dagsali, Aylin Izgis, Kadir Uzel, Mustafa Soytas, Niyazi Cakir, Abdullah Emre Askin, Ibrahim Azboy, Kubilay Sabuncu, Merve Aslan, Melek Sahin, Mustafa Oncel, Nuri Okkabaz, Ramazan Kemal Sivrikaya, Alparslan Saylar, Dr. Alparslan Saylar, Meltem Yasar [Istanbul Medipol University Hospital, Istanbul]; Ergin Erginoz*, Haktan Ovul Bozkir, Kagan Zengin, Mehmet Faik Ozcelik, Server Sezgin Uludag, Zeynep Ozdemir [Istanbul University Cerrahpasa - Cerrahpasa School Of Medicine]; Osman Sibic*, Hatice Telci, Mehmet Abdussamet Bozkurt, Yasin Kara [Kanuni Sultan Suleyman Training And Research Hospital, Istanbul]; Mustafa Deniz Tepe*, Adnan Gündoğdu, Bilge Akın, Dilan Pehlivan, Ali Guner, Duygu Baysallar, Berkay Yıldız, Hale Cepe, Murat Emre Reis, Ayse Nilufer Yuzgec, Nurtac Kıralı, Taha Anıl Kodalak, Mehmet Ulusahin [Karadeniz Technical University Farabi Hospital, Trabzon]; Kamar Selim*, Ahmet Kale, Mehmet Emre Gecici, Melis Ozbilen [Kartal Dr. Lutfi Kirdar Training And Research Hospital, Istanbul]; Zeynep Düzyol*, Aylin Gemici, Elzem Korkmaz, Eminenur Şen, Muhammed Enes Taşcı, Elifsu Camkıran, Güşta Elieyioğlu, İkbal Kayabaş, Tevfik Kıvılcım Uprak, Canan Aral, Ayten Saraçoğlu, Mustafa Ümit Uğurlu, Zeynep Hazal Baltacı [Marmara University, School Of Medicine, Istanbul]; Ege Nur Akkaya*, Cem Fergar, Elif Zeynep Tabak, Guldane Zehra Kocyigit, Ilgaz Kayilioglu [Mugla Training And Research Hospital, Mugla]; Süleyman Polat*, Eli̇f Çolak, Mehmet Emin Kara, Mert Candan, Mustafa Safa Uyanık, Ahmet Can Sarı [Samsun Training And Research Hospital, Samsun]; Attila Ulkucu*, Alperen Taha Certel, Arzu Dindar, Beyza Durdu, Cigdem Bayram, Eslem Kaya, Hakan Akdere, Ibrahim Ethem Cakcak, Ikranur Yavuz, Mert Omur, Mirac Ajredini, Erhan Onur Aydoğdu, Eylül Şenödeyici [Trakya University Faculty Of Medicine]; Ulku Ceren Koksoy*, Baturay Kansu Kazbek, Deniz Serim Korkmaz, Dogancan Yavuz, Hakan Yilmaz, Zeynep Sahan Cetınkaya, Elif Durmus, Filiz Tuzuner, Furkan Hokelekli, Mucahid Mutlu, Seyma Orcan Akbuz, Ziya Can Kus, Ziya Can Kus [Ufuk Üni̇versi̇tesi̇ Tip Fakültesi̇ Dr.Ri̇dvan Ege Sağlik Araştirma Uygulama Merkezi̇ Hastanesi̇, Ankara]

**United States of America**: Michael Farrell*, Alayna Craig-Lucas, Matthew Painter, [Lehigh Valley Health Network]; Ashley Titan*, Aditya Narayan, Bunmi Fariyike, Lisa Knowlton, Tiffany Yue [Stanford Health Care, Palo Alto, California]; Emily Benham*, Abdelrahman Nimeri, Hope Werenski, Nicole Kaiser, Caroline Reinke [Atrium Health]

*Local lead
